# Solvent Effects on the Conformational Preferences of 2,6‐Pyridylcarboxamide Foldamers

**DOI:** 10.1002/chem.71097

**Published:** 2026-05-08

**Authors:** Alexander R. Davis, Sena Öztürk, Kushal Samanta, Louise Male, Adam A. L. Michalchuk, Sarah J. Pike

**Affiliations:** ^1^ School of Chemistry University of Birmingham Birmingham UK

**Keywords:** chirality, circular dichroism, helical structures, solvent effects, supramolecular chemistry

## Abstract

The marked influence of organic solvents and water on the conformational preferences of a chiral, soluble 2,6‐pyridylcarboxamide foldamer has been established in an array of seventeen distinct solvents/solvent mixtures. Circular dichroism (CD) spectroscopy has been used to determine the conformational behavior of the soluble PEGylated foldamer as a function of the solvent for aromatic solvents (benzene, toluene and pyridine), chlorinated solvents (chloroform, dichloromethane and tetrachloroethane), polar aprotic solvents (tetrahydrofuran, acetone, acetonitrile, nitromethane, dimethylformamide and dimethylsulfoxide) and polar protic solvents (methanol, methanol/water mixtures). In aromatic and chlorinated solvents, the foldamer displays pronounced CD signals indicating the adoption of a stable helical conformation in these environments. Notably, even in the presence of polar protic solvents containing high levels of water (of up to 35% water in methanol mixtures), a helical conformation is still adopted by the foldamer, albeit with a less well‐defined helical conformation than for other less competitive solvents. These investigations provide crucial insights into the conformational preferences of this foldamer class in a wide range of organic solvents and water mixtures. It is anticipated that this solvent study will prove to be highly important for aiding the future design and operation of functional foldamers, including water compatible systems, in diverse environments.

## Graphical abstract

The marked effect of organic solvents and water/methanol mixtures on the conformational preferences of a soluble chiral foldamer has been established in a diverse array of seventeen distinct environments.

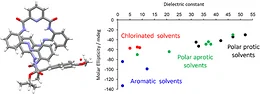

## 1 Introduction

Solvent effects play an integral role in governing the conformational behavior of biological systems, including regulating protein folding [1]. Solvation also has a significant influence on controlling the behavior of synthetic supramolecular systems, for example, in synthetic helical oligomers (foldamers) [2] through regulation of their self‐assembly and aggregation [3, 4, 5, 6]. Understanding the effect of solvent on the conformational behavior of foldamers is crucial to ensuring their effective design and operation as functional systems with applications in sensing [7], materials chemistry [8], synthesis [9], and biological chemistry [10]. Consequently, the solvation properties of foldamers and the dependency of the helix stability on the nature of the solvent, has been studied for a wide range of foldamer classes including aromatic oligoamides [11], *o*‐phenylene [12], *m*‐phenylene ethynylene [13], aryl trizole [14], *α*/sulfono‐*γ*‐AA peptidic [15] and aminoisobutyric acid (Aib) foldamers [16]. Solvents have also been shown to influence the ability of foldamers to bind guests [7, 17].

Huc and co‐workers have shown that in aromatic oligoamide foldamers, the helix stability displays a high dependency on the solvent [18]. Moreover, despite the widespread use of chloroform to study this foldamer class, surprisingly, of the solvents investigated in this study, it was identified as one of the least favorable solvents to stabilize these scaffolds. Notably, protic solvents were found to generate stable conformations in some aromatic oligoamide foldamers which is likely due to the solvent's ability to induce solvophobic effects and, thus, promote favorable intramolecular π—π stacking interactions within the foldamer [18]. The same group showed the importance of the primary sequence of these foldamers in dictating their secondary structure, by identifying solvent‐driven conformational transitions between extended linear/unfolded states and helically folded states upon the addition of water to dimethylsulfoxide solutions in more flexible foldamers of this class [11]. Gong and co‐workers have shown that the nature of the solvent can have an influence on the strength of self‐association of aromatic oligoamide foldamers which display crescent conformations [4]. For these relatively flat systems, which typically exhibit intermolecular π–π stacking interactions, strong self‐aggregation was observed in low polarity solvents (e.g., chloroform) but weak aggregation was seen in highly polar solvents (e.g., methanol). No significant changes to the crescent conformation in the studied aromatic oligoamide foldamers was reported [4].

Oligourea foldamers adopt stable helical conformations in a wide range of solvents including pyridine, acetonitrile, DMSO, and water [19, 20, 21, 22]. However, in the presence of a polar protic solvent, methanol, whilst an oligomer in this foldamer class was shown to adopt 2.5 helices, it was also seen to coexist with partially unfolded conformations in this polar protic solvent [22]. Overall, the ability of solvent to stabilize the helical folding of the oligourea foldamers was shown to follow the order pyridine >> acetonitrile > methanol > DMSO >> water [21].

Solvophobic foldamers based on *m*‐phenylene ethynylene (*m*PE) monomers, adopt solvent‐dependent conformations which range from compact helical states to disordered ones [23, 24, 25, 26]. Moore and co‐workers studied the influence of thirty solvents on the conformational behavior of *m*PE foldamers and discovered that chlorohydrocarbon solvents (e.g., chloroform, 1,2‐dichloroethane) completely denatured the system, significant defolding behavior was observed in cyclic ethers (e.g., tetrahydrofuran and 1,4‐dioxane) whilst high polarity solvents including DMSO, methanol and water induced highly stable folded conformations [23].

However, despite the importance of solvent effects on governing the conformational preferences of foldamers, systematic studies are still surprisingly rare. In particular, the influence of water on the folding behavior of these supramolecular scaffolds is vastly underexplored. Understanding their behavior in aqueous and biocompatible environments is highly important given the growing environmental and biological applications of foldamers. Accordingly, systematic investigations into the influence of solvent, in particular water, on the conformational behavior of foldamers are required for the development of these emergent supramolecular platforms but are surprisingly rare.

Herein, we report on a 2,6‐pyridylcarboxamide foldamer **1** whose incorporation of a chiral (*S*‐*+*)‐naproxen group at one terminus of the system and the solubilizing PEG group at the other termini of the scaffold enables the conformational preferences of the system to be established in a diverse array of solvents including methanol/water mixtures (Figure 1) [27]. Circular dichroism (CD) spectroscopy has been used to assess the influence of the nature of the solvent on the conformational preferences of the system including in apolar aprotic solvents (e.g., aromatic solvents benzene, toluene and pyridine), chlorinated solvents (chloroform, dichloromethane, tetrachloroethane), polar aprotic solvents (tetrahydrofuran, acetone, nitromethane, acetonitrile, dimethylformamide and dimethylsulfoxide) and polar protic solvents (methanol and methanol/water mixtures).

**FIGURE 1:**
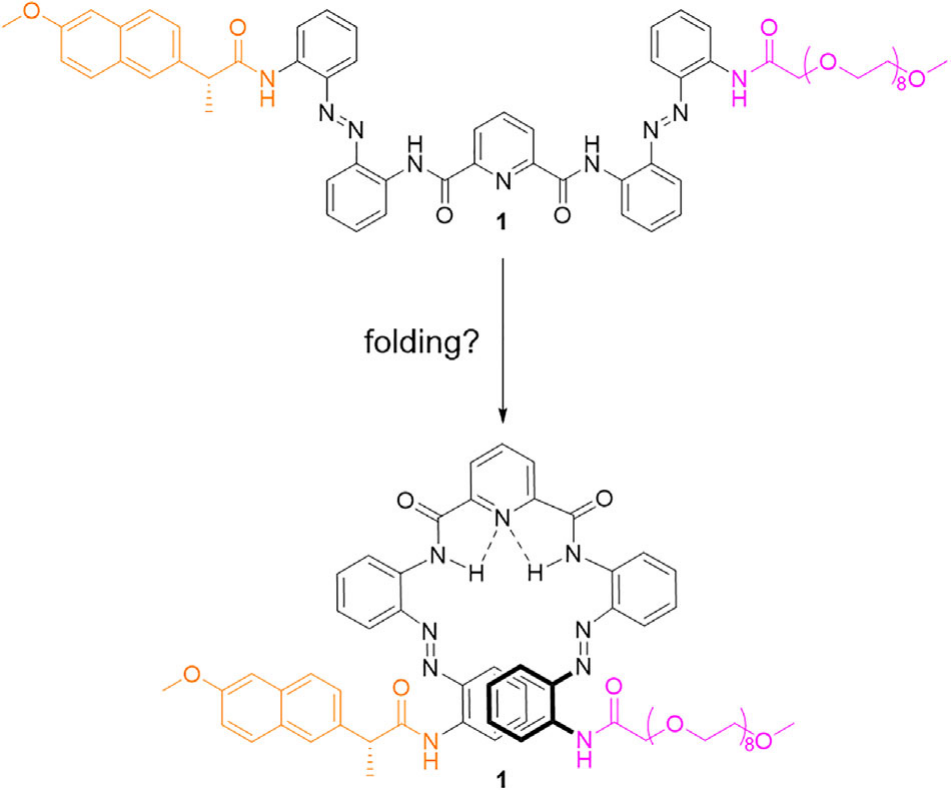
Soluble chiral foldamer **1** incorporating an enantiopure (S‐+)‐naproxen group at one terminus (shown in orange) and a solubilizing PEG chain at the other terminus (shown in purple). The conformational behavior of **1** was investigated in a diverse array of seventeen solvents/solvent mixtures including aromatic solvents (e.g., benzene), chlorinated solvents (e.g., chloroform), polar aprotic solvents (e.g., dimethylsulfoxide) and polar protic solvents (e.g., methanol and methanol/water mixtures).

## 2 Results and Discussion

### 2.1 Design and Synthesis of PEGylated Foldamer **1**

**1** has been designed to adopt a stable helical conformation in solution through the presence of two integral structural features embedded within the foldamer scaffold. First, the central pyridinecarboxamide unit enables the adoption of a *syn*‐*syn* conformation within **1** through the formation of a N─H⋯N⋯H─N hydrogen‐bonding interaction [28]. Second, the adoption of *E*‐configurations by the azobenzene units in **1** serves to further extend and support the helical structure of the scaffold (Figure 1) [29, 30]. Additionally, **1** incorporates a (*S*‐*+*)‐naproxen unit at one terminus in order to bias the helical screw‐sense preference of the system to pre‐dominantly populate one enantiomer over the other (i.e., *P*‐ over *M*‐helicity) [31]. Finally, the introduction of the PEG chain at the second terminus of asymmetrical **1** improves the solubility of the system [32] so that the foldamer's solution‐based behaviour can be studied in a diverse array of organic solvents and water/methanol mixtures.

**1** was synthesised in a linear multi‐step procedure in accordance with known or modified literature procedures (Scheme 1) [33, 34, 35, 36]. The asymmetric foldamer **3**, bearing amine and di‐*tert*‐butyl carbonate (Boc) capping groups, was accessed from *o*‐phenylenediamine **2** in five steps according to reported literature procedures [33, 34, 35, 36]. Treatment of **3** with an excess of (*S*‐*+*)‐naproxen chloride in the presence of pyridine and stirring at ambient temperature for 16 h generated **4** in 77%. The terminal Boc group in **4** was selectively deprotected through the addition of an excess of trifluoroacetic acid at ambient temperature to give the asymmetric amine and (*S*‐*+*)‐naproxen terminated **5** as a red solid in 93%. The PEG group was introduced to the foldamer scaffold through treatment of **5** with an excess of 3,6,9,12,15,18,21,24,27‐nonaoxaoctacosanoic acid chloride in the presence of pyridine to give **1** as an orange solid in 79%.

**SCHEME 1:**
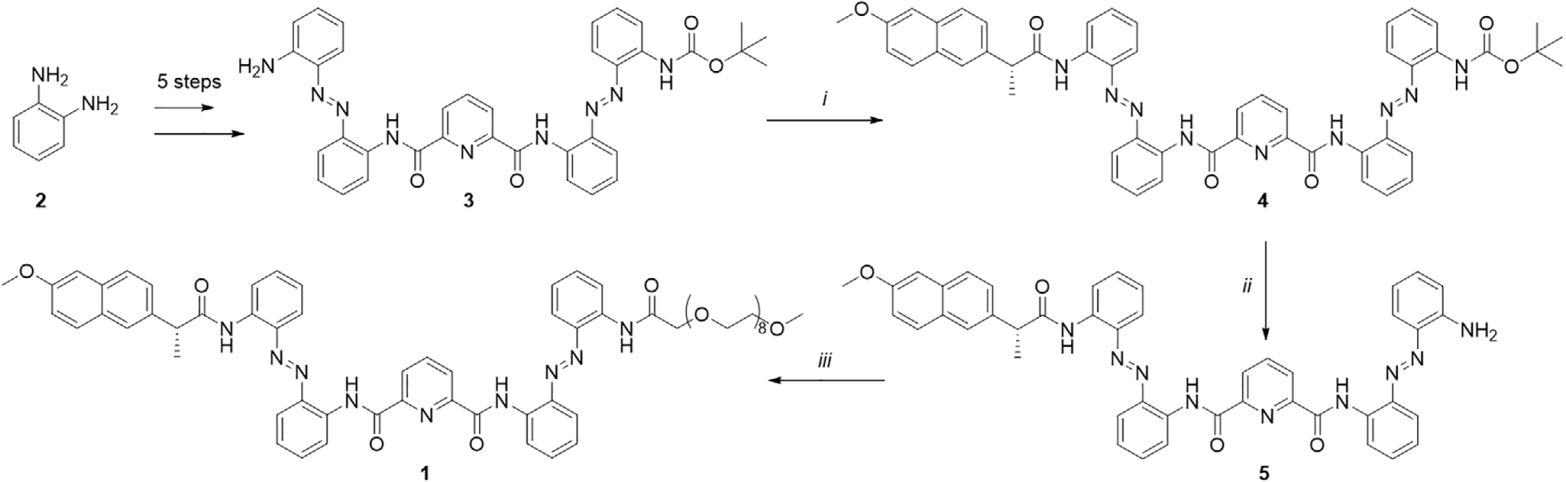
(i) (*S*‐*+*)‐naproxen, pyridine, CH_2_Cl_2_, 4 Å molecular sieves, rt, 16 h, 77%; (ii) trifluoroacetic acid, CH_2_Cl_2_, rt, 16 h, 93%; (iii) 3,6,9,12,15,18,21,24,27‐nonaoxaoctacosanoic acid chloride, pyridine, CH_2_Cl_2_, 4 Å molecular sieves, rt, 16 h, 79%.

**1** was fully characterised using ^1^H and ^13^C NMR spectroscopy, high resolution mass spectrometry, IR, circular dichroism and UV/Vis spectroscopy (Supporting Information). Unfortunately, attempts to grow single crystals of **1** suitable for x‐ray diffraction analysis were unsuccessful, presumably due to the conformational flexibility of the PEG chain. However, suitable crystals of the (*S*‐*+*)‐naproxen/Boc capped precursor foldamer **4** were grown through the slow evaporation of a di‐ethyl ether solution (Figure 2). In the unit cell of **4**, there is one foldamer molecule and one di‐ethyl ether solvent molecule which displays disorder across all atoms in the solvent molecule. There is also disorder about the (*S*‐*+*)‐naproxen group in **4**. Solid state analysis of **4** shows the adoption of a stable secondary structure displaying *P*‐helicity (Figure 2), due to the presence of the terminal (*S*‐*+*)‐naproxen group, which is in line with the solid‐state behaviour of related foldamers [33, 34, 35, 36]. **4** displays a one‐turn helical structure with a pitch of 4.6 Å [37]. Solid state analysis of **4** also shows that it is likely that the dominant factor governing helix stabilisation is the series of intramolecular N─H···N hydrogen bonds involving the pyridine and azo groups that support the *syn*‐*syn* conformation as well as the short N─H···N contacts present between the N atoms of the azo bonds and the H atom of the adjacent amide group (Supporting Information). Similar intramolecular N‐H···N hydrogen bonds and short N─H···N contacts have been reported by ourselves in foldamers belonging to the same class [33, 34, 35, 36] as well as by Parquette and co‐workers in their related pyridinedicarboxamide/*m*‐(phenylazo)azobenzene foldamers [29]. Whilst these series of intramolecular N─H···N hydrogen bonds and short N─H···N contacts involving the pyridine, azo and amide groups of the foldamer are likely to be the dominant factor governing helix stabilisation in this scaffold, there could well be other less prominent factors that could also help to stabilise the adoption of the helical conformation by the system. These likely include the intramolecular π···π stacking interactions which are observed between two aromatic rings in the foldamer core in the solid state of **4** (Supporting Information). Further solid state analysis of **4** shows that there are also intermolecular π···π and C–H···π interactions present in this system. Such intermolecular π···π and C–H···π interactions have been observed in the solid state structure of other foldamers in the same class [35, 36]. However, these intermolecular interactions are unlikely to play a significant role in governing helix stability of the system compared to the intramolecular N─H···N hydrogen bonds and short N─H···N contacts which support the adoption of the helix‐promoting *syn*‐*syn* conformation about the central pyridylcarboxamide unit of the foldamer core.

**FIGURE 2:**
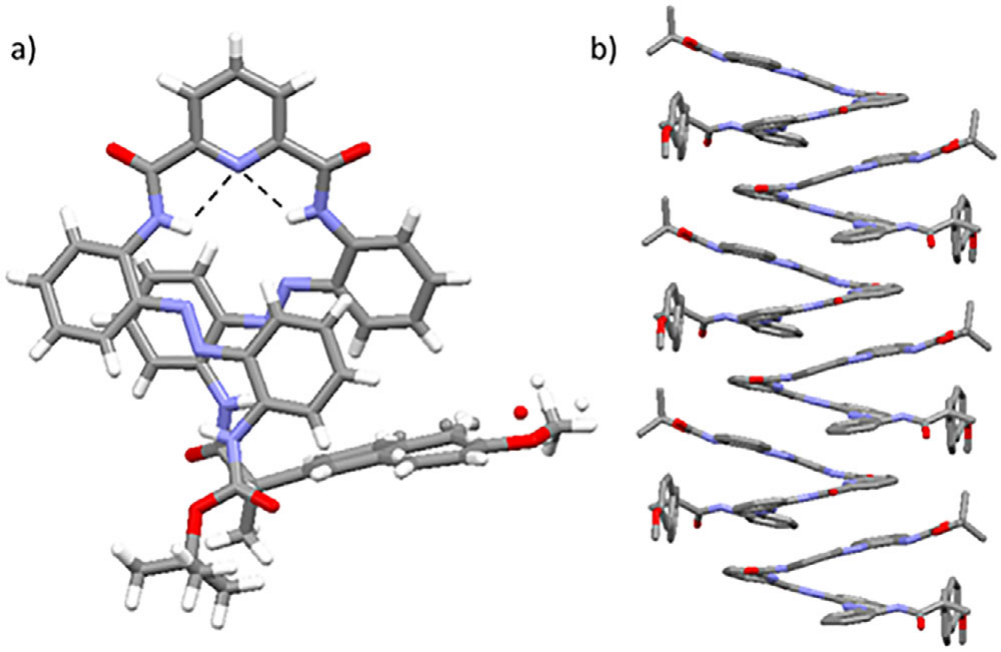
Crystal structure of the (*S*‐*+*)‐naproxen/Boc terminated foldamer precursor **4**; (a) highlighting the presence of a stable helical conformation and adoption of *P*‐helicity by the foldamer scaffold in the solid state and (b) showing the columnar stacking of helices in the crystal packing arrangement. C atoms are shown in grey, O in red, N in light blue. One molecule of di‐ethyl ether has been removed from (a) and (b) for clarity. Additionally, the disorder about the (*S*‐*+*)‐naproxen group and H atoms have been removed in (b) for clarity.

Furthermore, IR spectroscopic studies undertaken on **1** in three representative solvents of dimethylsulfoxide, tetrahydrofuran and toluene displayed no significant variation in the broad absorption band at ∼3345 cm^−1^ corresponding to the N─H stretches in the foldamer (Supporting Information). Hence, despite the vastly different polarities of the three tested solvents (ε = 2–47), it appears that solvent does not play a principal role in perturbing the intermolecular N─H···N hydrogen bonds that likely support helix stabilization.

### 2.2 Influence of Organic Solvents on the Conformational Preferences of the Foldamer

To obtain information about the global conformational behavior of the scaffold, CD spectroscopy was used to study the solution‐ based behavior of **1** in an array of different organic solvents. Accordingly, CD spectra of **1** were undertaken in benzene, pyridine, toluene, dichloromethane, chloroform, tetrachloroethane, tetrahydrofuran, acetonitrile, nitromethane, dimethylformamide, dimethylsulfoxide and methanol. The CD spectrum of a 1.5 × 10^−4 ^M toluene solution of **1** displays a large minimum centered around 475 nm which corresponds to the azobenzene moieties in the scaffold (Figure 3, blue line). A maximum centered at 398 nm, which is the absorption region associated with the azobenzene and aromatic oligoamide units of the foldamer, is also seen. The observed CD signal of **1** indicates the presence of a stable helical conformation in solution and the signs of the Cotton effects for the peaks at 475 nm (−84 mdeg) and 398 nm (58 mdeg) (negative and positive, respectively) suggests the presence a bias towards *P*‐helicity in this scaffold. This aligns with the solid‐state behaviour of precursor **4** and the solution behaviour of other related (*S*‐*+*)‐naproxen capped foldamers [31]. Similarly, CD spectra taken at 1.5 × 10^−4^ M in benzene, pyridine, dichloromethane, chloroform, tetrachloroethane, tetrahydrofuran, acetonitrile, nitromethane, dimethylformamide, dimethylsulfoxide and methanol solutions of **1**, all display a large minimum with negative Cotton effects between 466–475 nm and maximum with positive Cotton effects between 392–404 nm (Figure 3 and Supporting Information). These observed similarities in the CD spectra of **1** indicates that a stable helical conformation with a bias towards right‐ handedness is observed in all the studied solvents (Supporting Information) [31, 36]. Notably, however, there is a marked difference in the magnitude of the Cotton effects for the maxima between 466–475 nm (−133 to – 30 mdeg) and minima between 392–402 nm (16 to 58 mdeg) when **1** is studied in a variety of solvents (Figure 3, Table 1, and Supporting Information). For the large minimum with positive Cotton effects at 466–475 nm, the magnitude varies from −133 mdeg (benzene), −99 mdeg (pyridine), –84 mdeg (toluene), −57 mdeg (dichloromethane), −56 mdeg (chloroform), −55 (tetrachloroethane), −70 mdeg (tetrahydrofuran), −49 mdeg (acetonitrile), −64 mdeg (acetone), −49 mdeg (nitromethane), −44 mdeg (dimethylformamide), −30 mdeg (dimethylsulfoxide) and methanol (−55 mdeg) (Figure 3, Table 1, and Supporting Information) [38]. These CD studies indicate that stable helical conformations are adopted by **1** in all the tested organic solvents but the variations observed in the magnitude of the Cotton effects of minima of **1** could be due the varying levels of helical bias in the system and/or aggregation effects [39].

**FIGURE 3:**
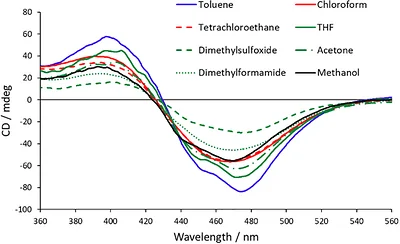
CD spectra of **1** in a selected range of studied organic solvents.

**TABLE 1:** Summary of solvent conditions, dielectric constant of the solvent and the molar ellipticity of the large minima centered between 466–475 nm in **1** as identified by CD spectroscopy.

Entry	Solvent	Dielectric Constant (ε)	Molar Ellipicity (mdeg)^a^
**1**	Benzene	2	−133
**2**	Pyridine	12	−99
**3**	Toluene	2	−84
**4**	Tetrahydrofuran	8	−70
**5**	Acetone	21	−64
**6**	Chloroform	5	−57
**7**	Dichloromethane	9	−56
**8**	Tetrachloroethane	8	−55
**9**	Methanol	33	−53
**10**	Acetonitrile	38	−49
**11**	Nitromethane	36	−49
**12**	Dimethylformamide	37	−44
**13**	Methanol/Water 95/5	32	−45
**14**	Methanol/Water 80/20	42	−42
**15**	Methanol/Water 70/30	47	−34
**16**	Dimethylsulfoxide	47	−30
**17**	Methanol/Water 65/35	52	−30

^a^ When **1 i**s studied in the outlined solvents, the centering of the large minima with the negative Cotton effect varies between 466–475 nm.

However, to assess the influence of the nature of the solvent on the self‐association behaviour of foldamer **1**, a series of ^1^H NMR spectroscopic dilution titration experiments were conducted in toluene‐d_8_, benzene‐d_6_, pyridine‐d_5_, dichloromethane‐d_2_, tetrachloroethane‐d_2_, chloroform‐d_1_, acetonitrile‐d_3_, nitromethane‐d_3_, tetrahydrofuran‐d_8_, dimethylsulfoxide‐d_6_ and dimethylformamide‐d_7_. Unfortunately, **1** was not soluble in methanol or methanol/water solvent mixtures at the NMR concentration range and, hence, could not be studied but the investigated solvents, do span a large solvent polarity range with dielectric constants ranging between 2 and 47. In each of the studied twelve solvents, the self‐association behaviour of **1** was studied over a 20‐fold range, typically between 0.8 – 14 mM (Supporting Information). ^1^H NMR spectroscopic analysis of the proton signals in **1** displayed no pronounced shifting behaviour in any of the studied solvents (Supporting Information). This indicates that notable self‐association does not occur for this foldamer in any of the studied systems. Consequently, it appears that aggregation does not play a role in governing the conformational behaviour of this system in the diverse range of solvents studied including those of widely varying polarities (i.e., with dielectric constants, ε = 2–47).

Analysis of the UV/Vis spectra of **1** taken at 4.4 × 10^−5^ M in toluene displays a band centred at 414 nm (Supporting Information). However, in more polar solvents (e.g., DMSO, methanol) hyprochromicity is observed (i.e., a reduction in the intensity of the peak) as well as a slight hypsochromic shifting of this band which could be indicative of the increased aryl ring stacking of the system in **1** induced by solvatophobic effects in more polar solvents (Supporting Information) [11, 23, 40].

Inspection of the dielectric constants, which is a measure of the solvent polarity [41], of the studied organic solvents and the magnitude of the Cotton effect observed for **1** at the minimum between 466–475 nm, shows that there is a general correlation with more polar solvents typically leading to more negative values for these minima (Figure 4). For example, the apolar aprotic solvent, toluene with ε = 2 exhibits minimum of −84 mdeg whilst the more polar solvents of dichloromethane and nitromethane with ε = 9 and 36 respectively display minima of −56 and −49 mdeg, respectively (Table 1). There also is some ordering of the conformational preferences of **1** based on the nature of the solvent class. For the aromatic solvents of benzene, pyridine, toluene, which possess a range of low to moderate dielectric constants (ε = 2–12), spectra with large magnitude Cotton effects −133 to −84 mdeg for the 401 nm centred minimum are observed. For the chlorinated solvents of chloroform, dichloromethane and tetrachloroethane, possessing dielectric constants of ε = 5 – 9, Cotton effects of −55 to −57 mdeg are seen, which are significantly less positive than that observed for the aromatic solvent of pyridine with ε = 12 of −133 mdeg. This data shows that it is the nature of the solvent (e.g., aromatic, chlorinated) and as well as the polarity of the solvent which plays a significant role in influencing the secondary structure of this foldamer class. This could be due to the presence of distinct solvent‐solute interactions which occur when changing between the different types of solvents. For example, unlike the other tested solvents, the aromatic solvents (i.e. benzene, pyridine and toluene) can engage in π–π stacking interactions with the foldamer whilst the chlorinated solvents could potentially form different solvent‐foldamer interactions (e.g., N─H⋯Cl hydrogen‐bonding interactions involving the chlorinated solvent and the foldamer) [33, 34]. For the polar aprotic solvents of acetonitrile, nitromethane, dimethylformamide and dimethylsulfoxide (ε = 38 – 47) smaller magnitude Cotton effects between −49 to −30 mdeg were seen for the minimum centred at 401 nm. In a similar manner, the polar protic solvent methanol, with a high dielectric constant of 33 displays a negative Cotton effect of −30 mdeg. However, despite this broad trend of more polar solvents generating larger magnitude Cotton effects in **1**, there are two outliers in the studied solvents; of tetrahydrofuran (ε = 8 and −70 mdeg for peak at 473 nm) and acetone (ε = 21 and −64 mdeg for peak at 472 nm). These results highlight that both the class of the solvent and its’ polarity can play an important role in influencing the foldamer's conformational preferences. However, the balance between these two factors is presumably influenced by the nature of the solvent‐foldamer interactions formed within the system which is dependent on the individual nature of the solvent, for example, unlike other solvents, the tested aromatics can form π–π stacking interactions with the foldamer meanwhile, in polar protic solvents can form O─H⋯O hydrogen‐bonding interactions with the foldamer which the other tested solvents are not able to.

**FIGURE 4:**
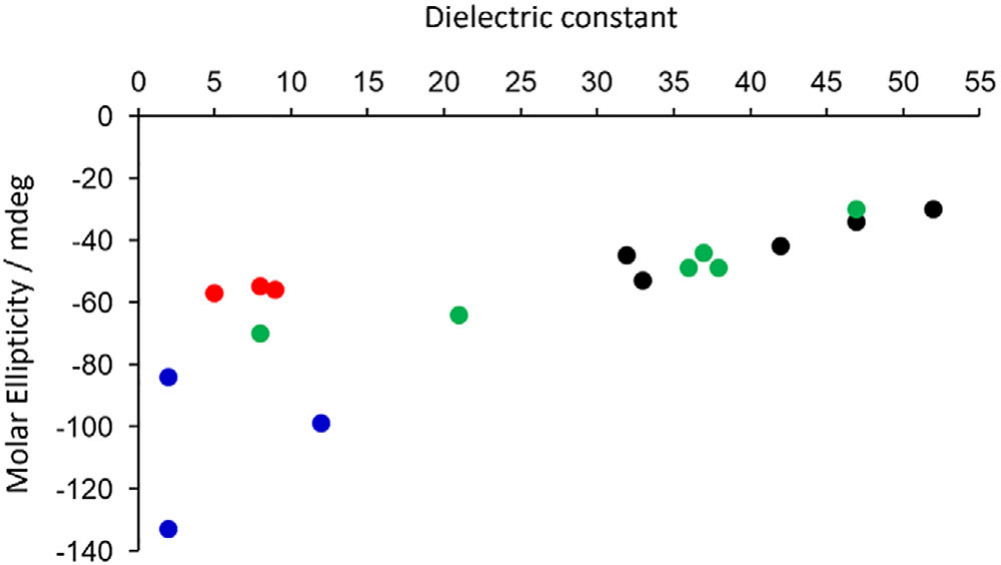
Graph showing the molar ellipticity of the minima of **1** centered between 466–475 nm as observed by CD spectroscopy in the seventeen studied solvents against their dielectric constants. Aromatic solvents shown in blue, chlorinated solvents shown in red, polar aprotic solvents shown in green and polar protic solvents shown in black.

### 2.3 Influence of Water on the Conformational Preferences of the Foldamer

Given the wider relevance of the future applications of foldamers in biology [42] and water‐compatible environments [43, 44, 45], the effect of water on the conformational preferences of **1** was investigated. Accordingly, CD spectra of **1** in methanol/water mixtures of 95/5, 80/20, 70/30 and 65/35 were undertaken (Table 1 and Figure 5). Attempts to record spectra of **1** in the presence of higher quantities of water in methanol/water mixtures were hampered by the limited solubility of **1** when the water content exceeded 35% in methanol. The spectrum of 1.5 × 10^−4^ M of **1** in a 95/5 methanol/water mixture displays a minimum at 468 nm and a maximum at 400 nm indicating the presence of a stable helical conformation in solution of *P*‐helicity as for the other studied organic solvents (Figure 3). The minimum at 468 nm exhibits a negative Cotton effect with a magnitude of −45 mdeg which is reduced from the value observed in pure methanol of −53 mdeg (Table 1 and Figure 5). Hence, the addition of water, even only 5% to a methanolic solution, leads to an observed change in the CD spectrum of **1** with a small shifting of the maximum and minimum and a reduction in the magnitude of their Cotton effects. These observed changes in the CD signal of **1**, upon the addition of water, are indicative of a lower level of helical bias in the system, a slight change to the conformation or differing aggregation properties of the system. This could be due to varying polarity of the solvent and/or the differing hydrogen‐bond donor and acceptor strengths of methanol and water which could influence the nature of the solvent‐foldamer interactions present in the system [46]. By further increasing the quantity of water present in the methanol/water solvent mixture, from 0%, 5%, 20%, 30% to 35%, further marked reductions in the magnitude of the Cotton effect of the minima at 468–470 nm from −53 mdeg at 0% to −30 mdeg at 35% were observed (Figure 5). This indicates that more pronounced changes to level of helical bias, conformational preferences and/or aggregation of the system are seen in the presence of increasing amounts of water. However, it should be noted that despite the presence of 35% of water, which is a highly competitive solvent [21], a stable secondary conformation is still adopted by **1** as shown through the presence of the minimum at 468 nm with the Cotton effect of −30 mdeg (Figure 5).

**FIGURE 5:**
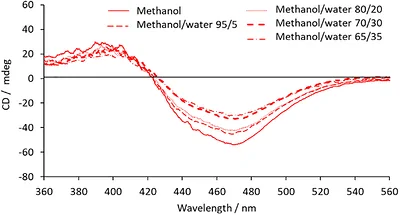
CD spectra of **1** in a selected range of studied methanol/water solvent mixtures.

Both methanol and water belong to the polar protic solvent class and, hence, will likely have the ability to form similar noncovalent interactions with the foldamer (e.g., O─H⋯Cl hydrogen‐bonding interactions) although their varying hydrogen‐bond acceptor and donor strengths might affect how favourable their formation is in the different methanol/water solvent mixtures [46]. Moreover, the dielectric constant of water is significantly higher than that of methanol and, hence, for the studied methanol/water solvent mixtures, the dielectric constants range from 33 – 52 upon increasing the quantity of water from 0% to 35% (Table 1) [47, 48, 49]. As for the organic solvents, a general trend of increasing the polarity of the solvent leads to a decrease in the magnitude of the Cotton effects seen in **1**. This indicates a lowering in the helical bias observed in the scaffold, a small change in the conformation of the system and/or the greater prominence of aggregation effects in the presence of increasing quantities of water.

### 2.4 Computational Studies

A series of calculations were undertaken to assess how the solvent influenced a number of key features associated with the solution‐based behaviour of the foldamer scaffold. These studies include i) investigating the effect of solvent on the lengths of the noncovalent interactions present in the core foldamer scaffold (i.e., those dominant factors that govern helix stabilisation), ii) the conformation adopted by the PEG chain and iii) the differences in energy of the two isomers of the foldamer (i.e., the *P*‐ and *M*‐helices) to ascertain their potential effect on the helical bias of the system as a function of solvent. Accordingly, DFT calculations were run on representative foldamer scaffolds with input geometries for the foldamer core taken from the crystal structure **4** (Figures 6 and 7). The geometry of the foldamer core was relaxed at B97‐D4/6‐31+G** level of theory and the optimised geometry was used as an input for exploring the effects of solvation using the polarised continuum model (PCM) in six representative solvents (chloroform, dimethylsulfoxide, methanol, tetrahydrofuran, toluene and water) [50, 51, 52]. These six solvents were selected as they represent a range of different solvent classes (i.e., apolar aprotic, chlorinated, polar aprotic and polar protic), and represent solvents of varying polarity (i.e., spanning a large dielectric constant range, ∈=2−47)Hence, these computational studies inform on the conformational behaviour of the foldamer in a diverse range of solvent types.

**FIGURE 6:**
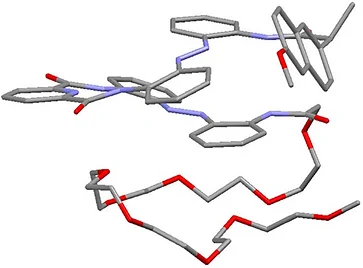
Global minimum geometry for **1** obtained at GFN‐XTB level of theory highlighting the optimized conformation of the PEG chain. C atoms are shown in grey, O in red, N in light blue. H atoms have been removed for clarity.

**FIGURE 7:**
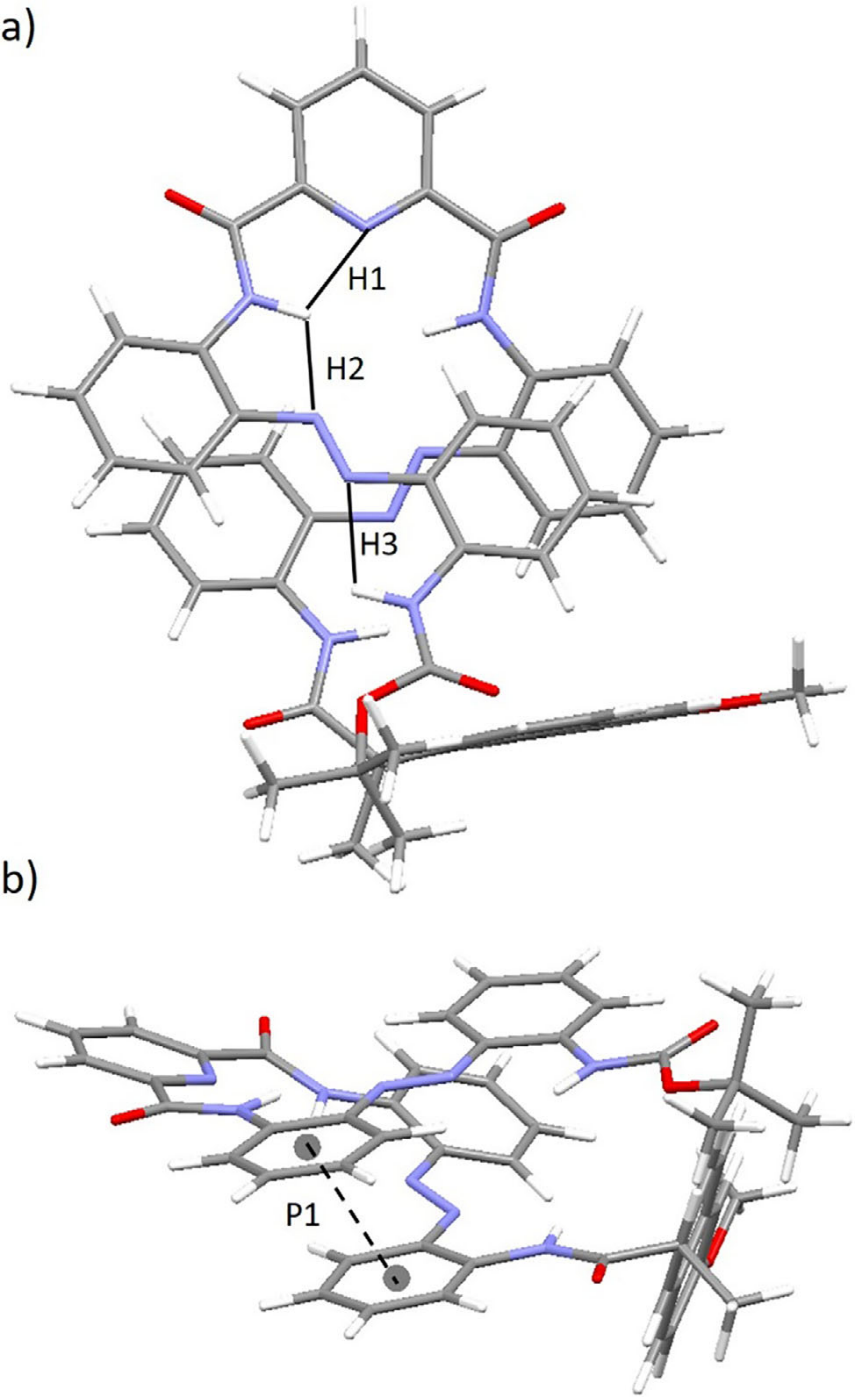
Optimized geometry of the foldamer core using DFT calculations. In (a) the black lines highlight the calculated bond lengths for the three distinct intramolecular N‐H···N hydrogen bonds and N‐H···N close contacts involving the pyridine, azo, and amide groups found in the foldamer core; (b) the dashed black line highlights the centroid‐centroid distance measured computationally to gain insights into the intramolecular π–π stacking interactions found between two aromatic rings in the foldamer core. C atoms are shown in grey, O in red, N in light blue, H atoms in white.

In the first instance we sought to identify to importance of the PEG chain on the foldamer stability. To do so, we manually added a linear PEG chain to a DFT‐optimised foldamer core taken from crystal structure **4**. The structure was optimised at DFT level, with the chain remaining in a largely linear conformation and the core remaining essentially unaltered (Supplementary Information). To better study the conformational landscape of the PEG chain, we froze the optimised core and performed a conformational search of the PEG using the geometry optimisation algorithm (GOAT) at GFN‐XTB level of theory. All of the identified (*ca* 200) conformations involved the PEG chain wrapping itself beneath the foldamer core, interacting with the foldamer core only through weak van der Waals interactions (Figure 6). We therefore posit that the PEG chain has little influence on the geometry of the foldamer core itself, and that, to a first approximation, one can study the stability of the foldamer core in the absence of the chain itself.

In this approximation, we subsequently explored the influence of solvent on the key noncovalent interactions that stabilise helical conformation within the foldamer core, with the aim to gain insights into their ability to perturb or support the folding behaviour of the system (Figure 7). Specifically, focus was placed on calculations concerning the intramolecular N─H···N hydrogen bonds and close N─H···N contacts involving the pyridine, azo and amide groups of the foldamer core (H1‐H3, Figure 7a). These interactions were studied as they promote the adoption of the *syn*‐*syn* conformation about the central pyridylcarboxamide unit and, hence, are likely the most dominant factor in helix stabilisation of the system. Crucially, the calculated bond lengths of these intramolecular N─H···N hydrogen bonds and close N─H···N contacts (H1‐H3, Figure 7a), which stabilise  the foldamer core, show no discernible difference in the six studied solvents. All calculated values for H1‐H3 in the gas phase and all six studied solvents show variations below *ca* 0.05 Å (Supporting Information). These results indicate that the solvent plays no significant role in perturbing the intramolecular N─H···N hydrogen bonds and close N─H···N contacts which are likely a dominant factor in helix stabilisation of this foldamer scaffold. Additionally, a second type of intramolecular non‐covalent interaction present within the foldamer core, of π–π stacking interactions between two aromatic rings in the foldamer core were studied (P1, Figure 7b). This interaction was selected to be studied as it is also likely to stabilise the adoption of the helical conformation by the scaffold, albeit to a smaller degree than the six consecutive intramolecular N─H···N hydrogen‐bonds and close N─H···N contacts (H1‐H3) promoting the adoption of the *syn*‐*syn* conformation within the core. Accordingly, to gain insight into the effect that solvent has on this likely helix‐stabilising intramolecular π–π stacking interaction, analysis of the centroid‐centroid distances between these two aromatic rings was undertaken (P1, Figure 7b). The calculations identified that in all six studied solvents, differences below *ca* 0.02 Å were observed for P1 (Supporting Information). Hence, these results indicate that there are no notable solvent effects on the strength of the intramolecular π–π stacking interactions present between two aromatic rings in the foldamer core. Accordingly, the helix‐stabilising intramolecular N─H···N hydrogen bonds and close N‐H···N contacts as well as the intramolecular π–π stacking interactions between two aromatic rings in the foldamer core display no noticeable variation in strength upon modification of the solvent across a large polarity range (ε = 2–47). Therefore, despite the diverse variation in the nature of the solvent, it appears that the system displays a strong propensity to adopt a folded structure as these two types of helix‐promoting intramolecular noncovalent interactions are seemingly largely unaffected by the nature of the solvent used in the study. These calculations are consistent with the foldamer remaining folded when in solution.

Finally, to assess whether solvent effects significantly influenced the helical bias of the system, a series of calculations were undertaken to determine the energy differences between the two different foldamer isomers (i.e., the *M*‐ and *P*‐helices), in the six outlined solvents (Supporting Information). In all cases regardless of the nature of the solvent, the original starting (*S*)‐enantiomer was found to be the lower energy system compared to the opposite (*R*)‐enantiomer (Supporting Information). A general trend of the energy differences between the *S* and *R* isomers was observed of toluene > chloroform ∼ tetrahydrofuran > DMSO ∼ methanol > water. Notably, the ordering of the solvents is broadly in line with observed for the CD minima in the different solvents (Table 1). The overall energy differences between the *S* and *R* isomers for the six studied solvents are within *ca* 20 kJ.mol^−1^ of each other, which likely means that the helical bias induced by the single, chiral naproxen group over the structure is not pronounced [53].

## 3 Conclusions

In conclusion, we have shown that a highly soluble PEGylated 2,6‐pyridylcarboxamide foldamer adopts a stable helical conformation in a diverse array of organic solvents and methanol/water mixtures. Circular dichroism spectroscopy has identified that aromatic solvents (e.g., benzene, toluene and pyridine), chlorinated solvents (e.g., chloroform, dichloromethane), polar aprotic solvents (e.g., tetrahydrofuran and dimethylsulfoxide) and polar protic solvents (methanol and methanol/water mixtures) all support the formation of well‐defined folded states by this foldamer class. Furthermore, a stable helical conformation is adopted by this foldamer class even in the presence of high quantities of water including to 35% of water in methanol/water mixtures. DFT calculations support these findings showing that the noncovalent intramolecular interactions within the foldamer core appear to be largely unaffected by the varying nature of the solvent used in the study.

Due to the widespread and growing interest in the role of solvent in regulating the conformational behaviour of foldamers, we envisage that this work will play a pivotal role in the future development and design of functional foldamers. Furthermore, these outlined studies, identifying the influence of water on the folding conformation of this supramolecular scaffold, could be particularly relevant to the progression of the field given the emerging applications of foldamers in biological and environmental uses, wherein water compatibility of the helical foldamer scaffold is often required for their successful operation.

## Conflicts of Interest

The authors declare no conflicts of interest.

## Supporting information



Supporting Information is available as a pdf file, including general methods, experimental procedures and full characterisation of new compounds including ^1^H and ^13^C NMR spectroscopy, mass spectrometry, melting point analysis, IR spectroscopy and, where possible, single crystal X‐ray diffraction analysis. Deposition number: https://www.ccdc.cam.ac.uk/services/structures?id = https://doi.org/10.1002/chem.71097 2440986 (for 4), contain(s) the supplementary crystallographic data for this paper. These data are provided free of charge by the joint Cambridge Crystallographic Data Centre and Fachinformationszentrum Karlsruhe http://www.ccdc.cam.ac.uk/structures ″>Access Structures service. Additional references cited within the Supporting Information [29, 54, 55, 56, 57, 58, 59, 60, 61]. **Supporting File**: chem71097‐sup‐0001‐SuppMat.pdf

## Data Availability

The data that support the findings of this study are available in the supplementary material of this article.
