# Supplementary material for: Solvent Effects on the Conformational Preferences of 2,6‐Pyridylcarboxamide Foldamers
Source: Chemistry. 2026 May 8;32(37):e71097. doi: 10.1002/chem.71097 (PMC13628530; doi:10.1002/chem.71097)
Supplement: Supplementary file 1 [file CHEM-32-e71097-s001.pdf]

Supporting Information

Solvent Effects on the Conformational Preferences of 2,6-Pyridylcarboxamide Foldamers

Alexander R. Davis,[a] Sena Öztürk,[a] Kushal Sumanta,[a] Louise Male,[a]

Adam A. L. Michalchuk[a]* and

Sarah J. Pike[a]*

[a]School of Chemistry, University of Birmingham, Edgbaston, Birmingham, B15 2TT, UK.

Email: s.j.pike@bham.ac.uk

Table of Contents

General Experimental Details 3

Crystallographic Details 5

Synthetic Details 6

COSY and NOESY Spectra 12

Crystal Data and Structural Refinement 18

Solid State Analysis 19

Circular Dichroism Spectroscopy 25

UV/Vis Spectroscopy 36

^{1}H NMR Dilution Studies 45

IR Spectroscopy 59

Computational Studies 62

Appendix: ^{1}H and ^{13}C NMR spectra 66

References 72

General Experimental Details

All NMR spectroscopy was carried out on a Bruker AVIII300, Bruker AVIII400, Bruker NEO400 and Bruker AV4-500 NMR spectrometer using the residual solvent as the internal standard. All the chemical shifts (δ) are quoted in ppm and coupling constants are given in Hz and are rounded to 0.1 Hz. Splitting patterns are abbreviated as follows: s (singlet), br (broad), d (doublet), t (triplet), m (multiplet) or some combination of these. Thin layer chromatography (TLC) was performed using commercially available precoated (Macherey-Nagel alugram. Sil G/UV_{254}) and visualized with UV light at 254 nm. Flash column chromatography was carried out using Fluorochem Davisil 40-63u 60Å. Melting points were obtained on a Gallenkemp melting point apparatus and are uncorrected. Mass spectra were obtained using Waters LCT (ES), Waters Synapt (ES) or Bruker MicroTOF spectrometers, each fitted with TOF detector. UV/Vis spectroscopic studies were carried out Agilent Cary 3500 UV/Vis photospectrometer fitted with a temperature control unit. Circular dichroism spectroscopic studies were carried out a Jasco J-1500 spectropolarimeter or Jasco J1100ST circular dichroism spectrophotometer fitted with a temperature control unit. Infrared spectroscopy was carried out on a PerkinElmer Spectrum Two FT-IR spectrometer. Petrol refers to the fraction of light petroleum ether boiling between 40 and 60 °C. (*S*)-(+)-naproxen chloride was purchased from Sigma Aldrich. Pyridine, trifloroacetic acid, acetone, dimethylformamide and chloroform were purchased from Thermofisher Scientific Chemicals. Acetonitrile and dimethyl sulfoxide were purchased from Sigma Aldrich. Nitromethane was purchased from Acros Organics. Tetrahydrofuran and dichloromethane were purchased from Sigma Aldrich. Toluene, ethyl acetate and petroleum ether were purchased from VWR. 3,6,9,12,15,18,21,24,27-Nonaoxaoctacosanoic acid was purchased from Fluorochem. All chemicals were used as received. **2** was prepared according to known literature procedures.[1,2]

The following abbreviations are employed: aq. = aqueous, Boc = *tert*-butyloxycarbonyl, DCM = dichloromethane, DMF = *N,N*-Dimethylformamide, DMSO = dimethylsulfoxide, d = doublet, eq. = equivalent(s), et = ethyl, g = gram(s), GOAT = geometry optimisation algorithm, h = hour(s), ^{i}Pr = isopropyl, IR = infared, Me = methyl, MeCN = acetonitrile, m.p. = melting point, min = minute(s), MS = molecular sieves, m = multiplet, PEG – polyethylene glycol, PCM = polarised continuum model, pet. = petroleum, q = quartet, s = singlet, t = triplet, TCE = 1,1,2,2-tetrachloroethane, THF = tetrahydrofuran.

Crystallographic Details

A suitable crystal of **4** were selected and collected on a XtaLAB Synergy, Dualflex, HyPix diffractometer. The crystal of **4** was kept at 99.99(10) K during data collection. Using Olex2,[3] the structure was solved with the ShelXT[4] structure solution program using Intrinsic Phasing and refined with the ShelXL[5] refinement package using Least Squares minimisation.

4: The ring system C(42)-C(43), C(46)-C(49), O(6)-C(50) / C(42)-C(43), C(46A)-C(48A), C(49), O(6A)-C(50A) is disordered over two positions at a refined percentage occupancy ratio of 67.2 (6) : 32.8 (6). The structure contains a molecule of diethyl ether, C(101)-C(104), O(101) / C(11A)-C(14A), O(11A) is disordered over two positions at a refined percentage occupancy ratio of 73.9 (5) : 26.1 (5). The hydrogen atoms bonded to N(2), N(5), N(6) and N(9) were located in the electron density and the positions refined freely. All remaining hydrogen atoms were fixed as riding models with the isotropic thermal parameters (U_{iso}) of all hydrogen atoms in the structure based on the U_{eq} of the parent atom. It was not possible to confirm the absolute structure from the diffraction data as, as such the assignment of S is confirmed from the synthetic scheme.

The CCDC number for **4** is 2440986 and this entry contains the supplementary crystallographic data for this paper. These data are provided free of charge by the joint Cambridge Crystallographic Data Centre and Fachinformationszentrum Karlsruhe through www.ccdc.cam.ac.uk/structures.

Synthetic Details

4

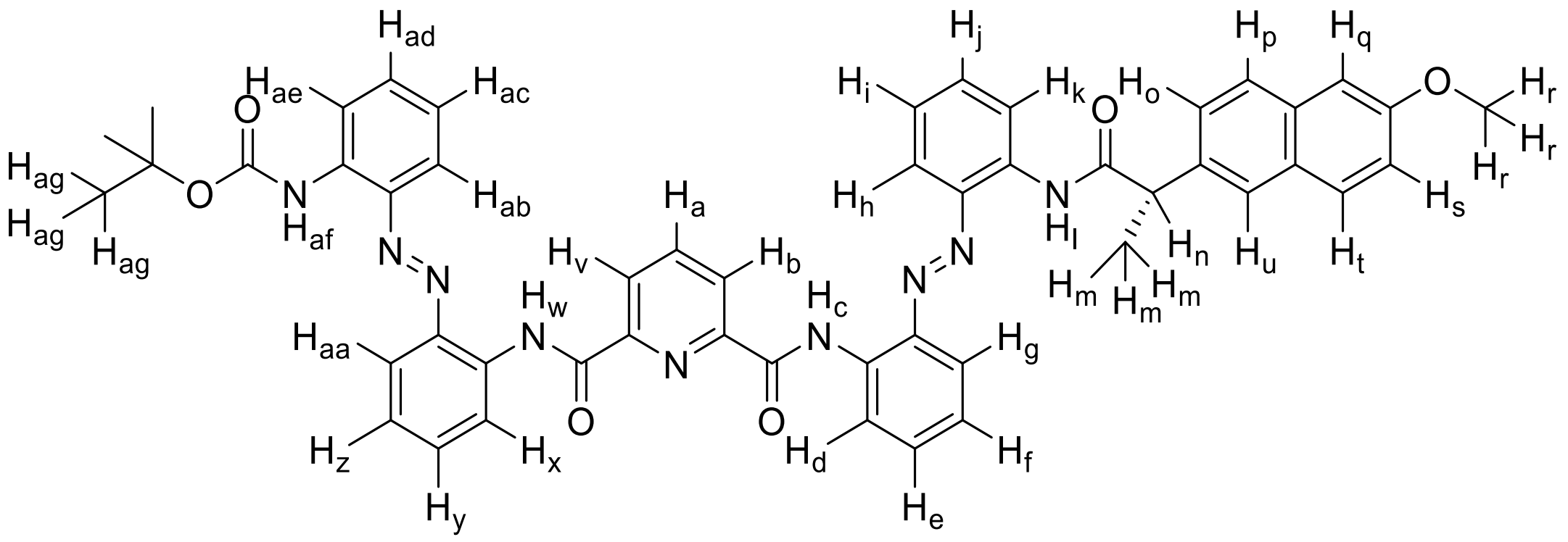

A solution of **3** (0.52 g, 0.79 mmol) was prepared in CH_2Cl_2 (10 mL), pyridine (2 mL) and 4 Å molecular sieves (0.2 g) were added, the resultant red solution was stirred at ambient temperature for 30 min. After this time, (*S*)-(+)-naproxen chloride (0.98 g, 3.95 mmol) was added to the reaction mixture and the resultant bright orange solution was stirred at ambient temperature for 16 h. The mixture was diluted with CH_2Cl_2 (10 mL), and the molecular sieves were filtered off. The organic layer was washed with water (2 × 15 mL), and brine (1 × 15 mL), then dried over $MgSO_4$ and filtered. The excess solvent was removed under reduced pressure, and the resultant residue was purified using flash column chromatography; (pet. ether/EtOAc; 70 : 30), to yield the desired product as an orange solid (0.53 g, 77 %). m.p. 173-174 °C. ^{1}H NMR (400 MHz, $CDCl_3$, 298 K) δ 11.39 (s, 1H, NH_c), 11.33 (s, 1H, NH_w), 8.85 (dd, ^3J = 8.4, ^4J = 1.3 Hz, 1H, ArH_d), 8.78 (s, 1H, NH_l), 8.75 (dd, ^3J = 8.4, ^4J = 1.3 Hz, 1H, ArH_x), 8.56 – 8.49 (m, 2H, ArH_{b+v}), 8.43 (dd, ^3J = 8.4, ^4J = 1.3 Hz, 1H, ArH_k), 8.24 (s, 1H, NH_{af}), 8.21 (dd, ^3J = 8.5, ^4J = 1.3 Hz, 1H, ArH_{ae}), 8.17 (t, ^3J = 7.8 Hz, 1H, ArH_a), 7.77 (d, ^3J = 8.9 Hz, 1H, ArH_p), 7.73 – 7.68 (m, 2H, ArH_{q+t}), 7.53 (ddd, ^3J = 8.6, 7.3, ^4J = 1.5 Hz, 1H, ArH_e), 7.37 – 7.30 (m, 3H, ArH_{g+s+y}), 7.27 – 7.22 (m, 2H, , ArH_{h+o}), 7.20 – 7.16 (m, 2H, ArH_{u+ab}), 7.13 (ddd, ^3J = 8.2, 7.3, ^4J = 1.3 Hz, 1H, ArH_f), 6.87 – 6.78 (m, 2H, ArH_{j+ad}), 6.18 – 6.10 (m, 2H, , ArH_{j+z}), 5.88 (ddd, ^3J = 8.3, 7.3, ^4J = 1.3 Hz, 1H, ArH_{ac}), 5.79 (dd, ^3J = 8.2, ^4J = 1.5 Hz, 1H, ArH_{aa}),

4.00 – 3.91 (m, 4H, CH_3 + CH, H_{n+r}), 1.64 (d, ^3J = 7.2 Hz, 3H, CH_3, H_m), 1.58 (s, 9H, 3 × CH_3, H_{ag}). ^{13}C NMR (101 MHz, $CDCl_3$, 298 K) δ 172.4, 161.4, 161.2, 158.0, 152.2, 149.7, 149.7, 140.0, 139.8, 139.7, 139.2, 138.1, 137.9, 137.5, 136.9, 136.7, 136.1, 133.9, 132.9, 132.9, 132.8, 132.8, 129.5, 129.2, 128.1, 127.3, 126.3, 125.9, 125.8, 124.1, 123.7, 122.8, 121.5, 120.2, 119.7, 119.6, 119.4, 118.0, 116.4, 115.6, 114.9, 114.1, 106.0, 81.2, 55.5, 48.3, 28.4, 18.8. IR (solid) ν_{max} 3394, 3354, 2916, 2849, 1720, 1686 cm^{-1}; LR-ESIMS (+ve): 868.36 [M+H] +; HRESI(+ve): calcd. 868.3571 found 868.3589 for C50H45N9O6.

5

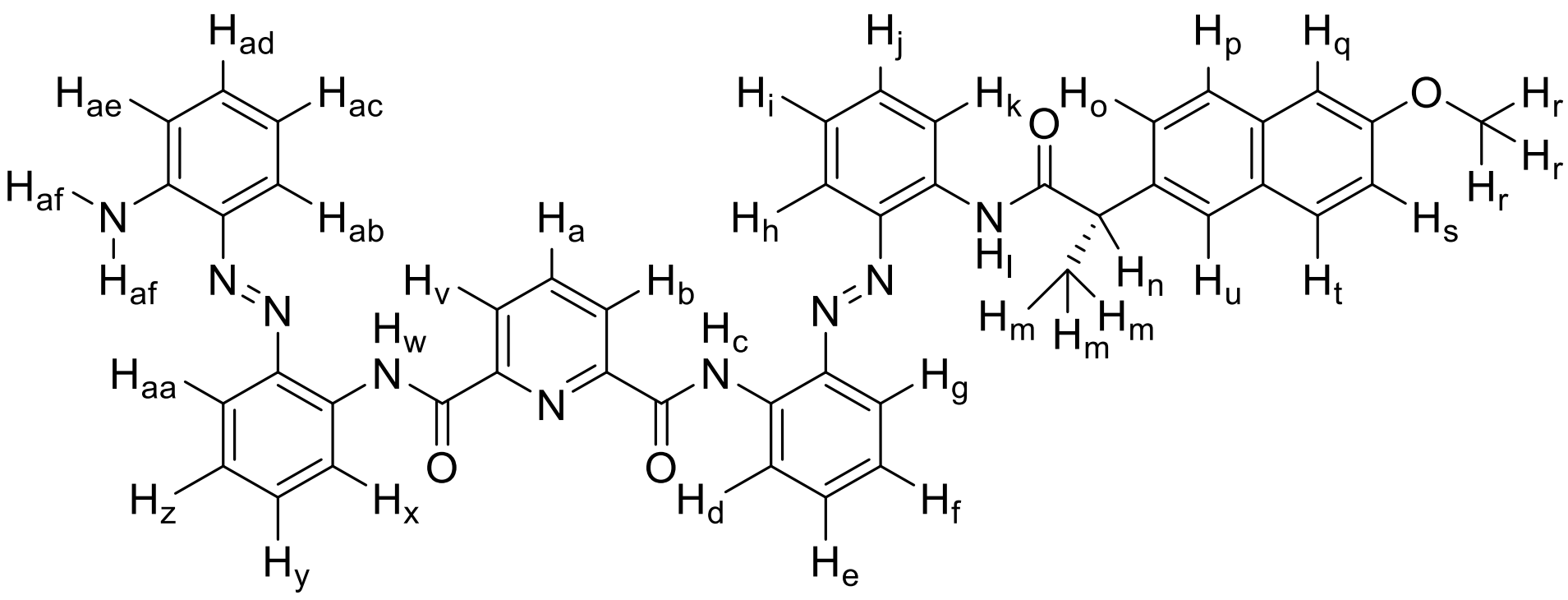

To a solution of **4** (0.53 g, 0.61 mmol) dissolved in CH_2Cl_2 (30 mL) trifluoroacetic acid (0.23 mL, 3.1 mmol) was added in a dropwise manner, the solution changed colour to black and the resultant mixture was allowed to stir at ambient temperature for 18 h. After this time, the black mixture was then quenched with an excess of NaOH (1M) and allowed to stir for 1 h to produce a red solution. The organic phase was then extracted with CH_2Cl_2 (2 × 20 mL) and washed with brine (1 × 30 mL), before drying with $MgSO_4$ and filtered. The excess solvent was removed under reduced pressure, and the resultant residue was purified using flash column chromatography; (pet. ether/EtOAc; 60 : 40), to yield the product as red solid (0.44 g, 93 % yield). m.p. 134-135 °C. ^{1}H NMR (400 MHz, CDCl3, 298 K): δ 11.64 (s, 1H, NH_w), 11.47 (s, 1H, NH_c), 8.99 (s, 1H, NH_l), 8.80 (dd, ^3J = 8.3, ^4J = 1.3 Hz, 1H, ArH_d), 8.72 (dd, ^3J = 8.3, ^4J = 1.3 Hz, 1H, ArH_x), 8.53 (m, 2H, ArH_{b+v}), 8.39 (dd, ^3J = 8.3, ^4J = 1.3 Hz, 1H, ArH_k), 8.19 (t, ^3J = 7.8 Hz, 1H, ArH_a), 7.86 – 7.73 (m, 3H, ArH_{p+q+t}), 7.49 – 7.42 (m, 2H, ArH_{e+s}), 7.39 (ddd, ^3J = 8.5, 7.3, ^4J = 1.5 Hz, 1H, ArH_y), 7.30 – 7.18 (m, 6H, Ar$H_{h+g+o+u+ab}$), 7.03 (ddd, ^3J = 8.3, 7.3, ^4J = 1.3 Hz, 1H, ArH_f), 6.84 (ddd, ^3J = 8.6, 7.3, ^4J = 1.5 Hz, 1H, ArH_j), 6.53 (ddd, ^3J = 8.3, 7.2, ^4J = 1.3 Hz, 1H, ArH_z), 6.48 (ddd, ^3J = 8.4, 7.0, ^4J = 1.5 Hz, 1H, ArH_{ad}), 6.39 (dd, ^3J = 8.2, ^4J = 1.5 Hz, 1H, ArH_{aa}), 6.15 (ddd, ^3J = 8.3, 7.2, ^4J = 1.3 Hz, 1H, ArH_i), 5.91 (ddd, ^3J = 8.2, 7.1, ^4J = 1.3 Hz, 1H, ArH_{ac}), 5.84 (apparent d, ^3J = 8 Hz, 1H, ArH_{ae}), 4.62 (s, 2H, NH_{af}), 4.03 – 3.94 (m, 4H, CH_3 + CH, CH_{n+r}), 1.71 (d, ^3J = 7.1 Hz, 3H, CH_m). ^{13}C NMR (101 MHz, $CDCl_3$,

298 K) δ 172.2, 161.4, 161.0, 158.1, 149.9, 149.6, 144.4, 140.3, 139.6, 139.6, 139.2, 137.4, 137.1, 137.1, 136.4, 135.2, 133.9, 132.6, 132.6, 132.3, 131.4, 129.4, 129.2, 128.0, 126.8, 126.3, 125.7, 125.5, 123.9, 123.5, 123.0, 120.4, 119.8 (2C), 119.7, 119.5, 118.0, 117.2, 116.1, 114.6, 114.1, 105.9, 55.5, 48.6, 18.7. IR (solid) ν_{max} 3476, 3352, 3056, 2934, 1681, 1587 cm^{-1}; LR-ESIMS (+ve): 768.31 [M+H] +; HRESI(+ve): calcd. 768.3047 found 768.3044 for C45H37N9O4.

1

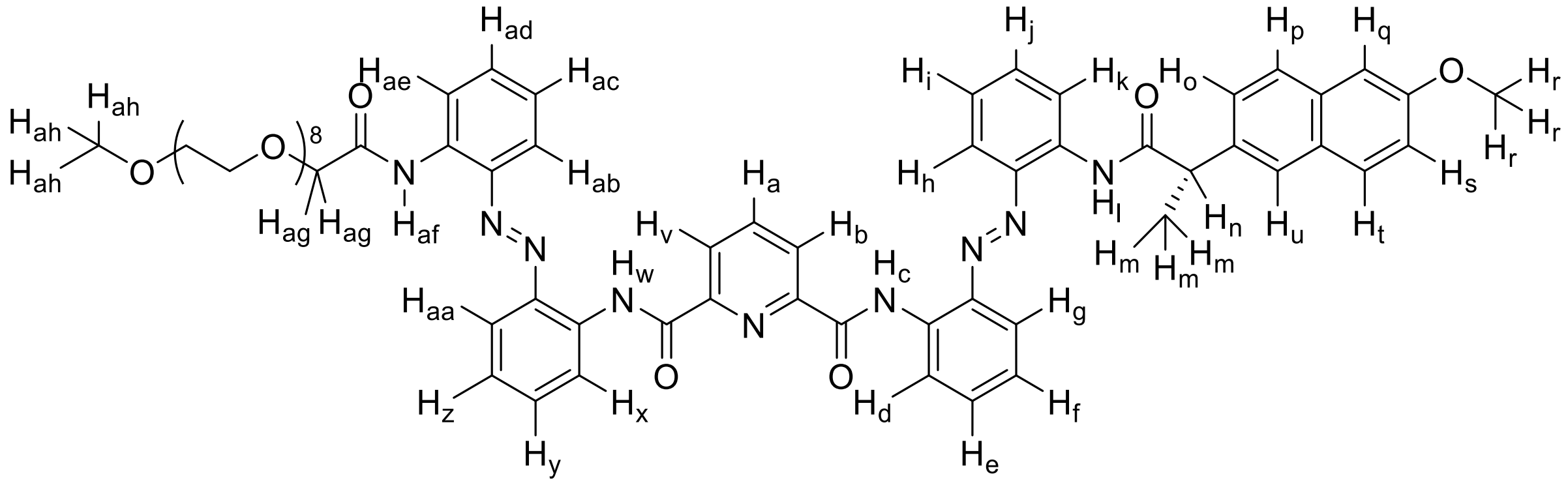

A solution of **5** (0.47 g, 0.61 mmol) was prepared in CH_2Cl_2 (10 mL), pyridine (4 mL) and 4 Å molecular sieves (0.2 g) were added and the resultant red solution was stirred at ambient temperature for 30 min. A solution of 3,6,9,12,15,18,21,24,27-nonaoxaoctacosanoic acid chloride was prepared in CH_2Cl_2 (5 mL) and was added to the reaction mixture, the resultant bright orange solution was stirred at ambient temperature for 16 h. The excess solvent was removed under reduced pressure, and the resultant residue was purified using flash column chromatography; (pet. ether/EtOAc; 0 – 100 %, then EtOAc/MeOH; 95 : 5), excess solvent was removed under reduced pressure and residue was dissolved in MeOH (60 mL). The MeOH was washed with hexane (2 × 20 mL) and then removed under reduced pressure to yield the desired product as a waxy orange solid (0.57 g, 79 %). ^{1}H NMR (400 MHz, CDCl3, 298 K) δ 11.38 (s, 1H, NH_w), 11.23 (s, 1H, NH_c), 9.99 (s, 1H, NH_{af}), 8.89 (dd, ^3J = 8.4, ^4J = 1.7 Hz, 1H, ArH_d), 8.79 (s, 1H, NH_l), 8.75 (dd, ^3J = 8.4, ^4J = 1.3 Hz, 1H, ArH_x), 8.57 (dd, ^3J = 8.3, ^4J = 1.2 Hz, 1H, ArH_{ae}), 8.55 – 8.50 (m, 2H, ArH_{b+v}), 8.48 (dd, ^3J = 8.5, ^4J = 1.3 Hz, 1H, ArH_k), 8.16 (t, ^3J = 7.8 Hz, 1H, ArH_a), 7.82 – 7.75 (m, 2H, ArH_{p+q}), 7.72 (d, ^3J = 8.5 Hz, 1H, ArH_t), 7.53 (ddd, ^3J = 8.6, 7.2, ^4J = 1.6 Hz, 1H, ArH_e), 7.39 – 7.29 (m, 3H, ArH_{g+s+y}), 7.25 – 7.14 (m, 4H, Ar$H_{h+o+u+ab}$), 7.09 (ddd, ^3J = 8.4, 7.2, ^4J = 1.4 Hz, 1H, ArH_f), 6.81 (ddd, ^3J = 8.6, 7.3, ^4J = 1.5 Hz, 1H, ArH_j), 6.75 (ddd, ^3J = 8.5, 7.3, ^4J = 1.5 Hz, 1H, ArH_{ad}), 6.14 (ddd, ^3J = 8.4, 7.3, ^4J = 1.3 Hz, 1H, ArH_i), 6.04 (ddd, ^3J = 7.7, ^4J = 1.3 Hz, 1H, ArH_z), 5.95 (ddd, ^3J = 7.7, ^4J = 1.3 Hz,

1H, ArH_{ac}), 5.79 (dd, ^3J = 8.2, ^4J = 1.4 Hz, 1H, ArH_{aa}), 4.20 (d, ^3J = 2.8 Hz, 2H, PEG CH_2, ArH_{ag}), 4.00 – 3.92 (m, 4H, CH_3 + CH, CH_{n+r}), 3.87 – 3.78 (m, 2H, PEG CH_2), 3.75 – 3.71 (m, 2H, PEG CH_2), 3.69 – 3.59 (m, 26H, PEG CH_2), 3.54 – 3.50 (m, 2H, PEG CH_2), 3.35 (s, 3H,PEG CH_2, CH_{ah}), 1.63 (d, ^3J = 7.2 Hz, 3H, CH_3, H_m). ^{13}C NMR (101 MHz, CDCl3, 298 K) δ 172.7, 167.9, 161.4, 161.3, 158.0, 149.8, 149.8, 140.3, 139.7, 139.6, 138.8, 138.7, 137.9, 137.2, 137.1, 137.0, 136.1, 134.0, 133.2, 132.9, 132.9 (2C), 129.6, 129.2, 128.0, 127.6, 126.5, 126.0 (2C), 123.9, 123.7, 123.1, 122.8, 120.2, 119.9, 119.5, 119.2, 119.2, 115.9, 114.8, 114.2, 114.0, 106.0, 72.0, 71.2, 70.8, 70.6 (13C from PEG group), 70.3, 59.1, 55.5, 48.4, 18.9. IR (solid) ν_{max} 3355, 3056, 2919, 2868, 2854, 1686, 1589, 1516 cm^{-1}; LR-ESIMS (+ve): 1214.51 [M+Na] +; HRESI(+ve): calcd. 1214.5175 found 1214.5144 for C64H73N9O14.

COSY and NOESY Spectra

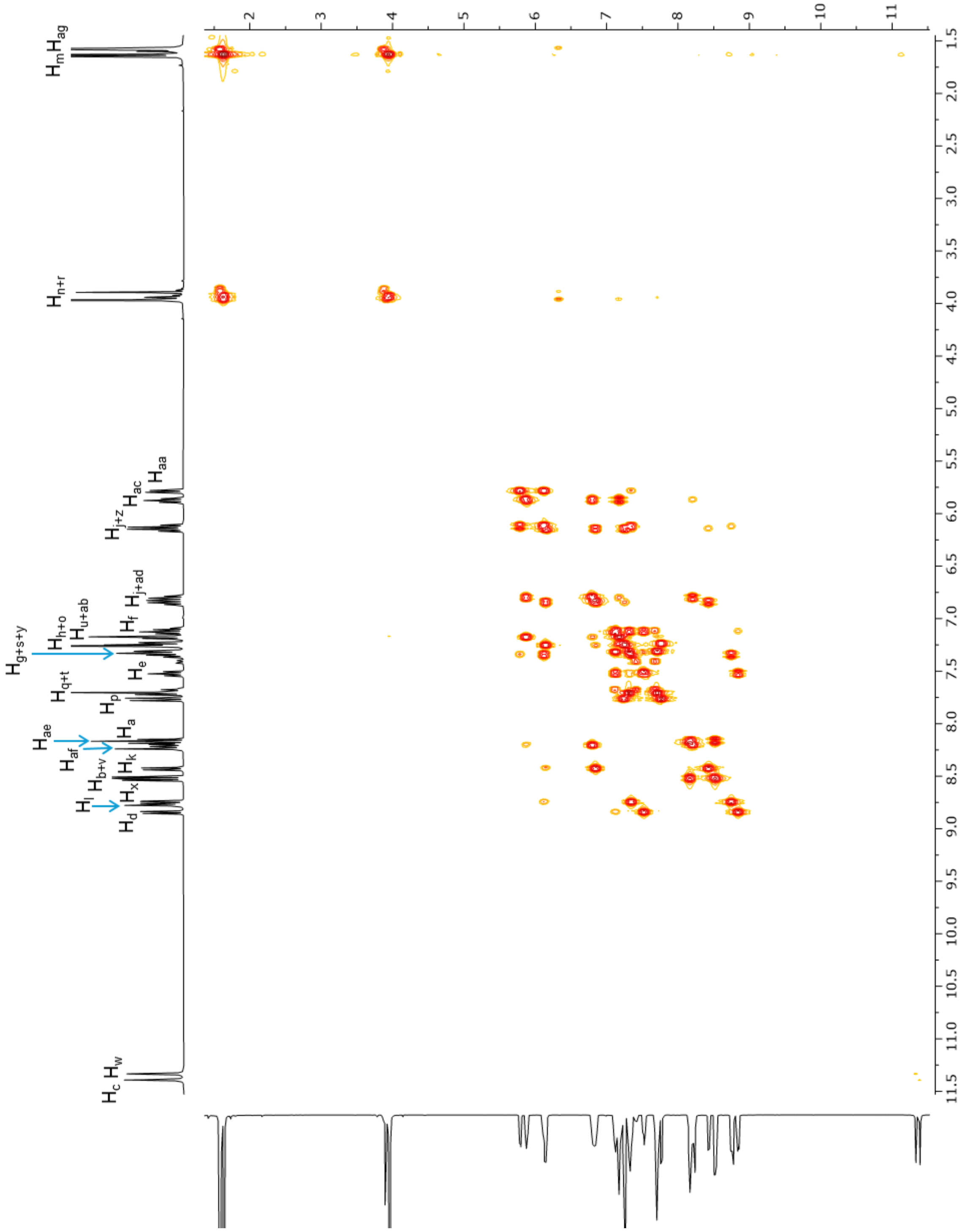

Figure S 1: Partial COSY NMR spectrum of **4** (400 MHz, CDCl3, 298 K).

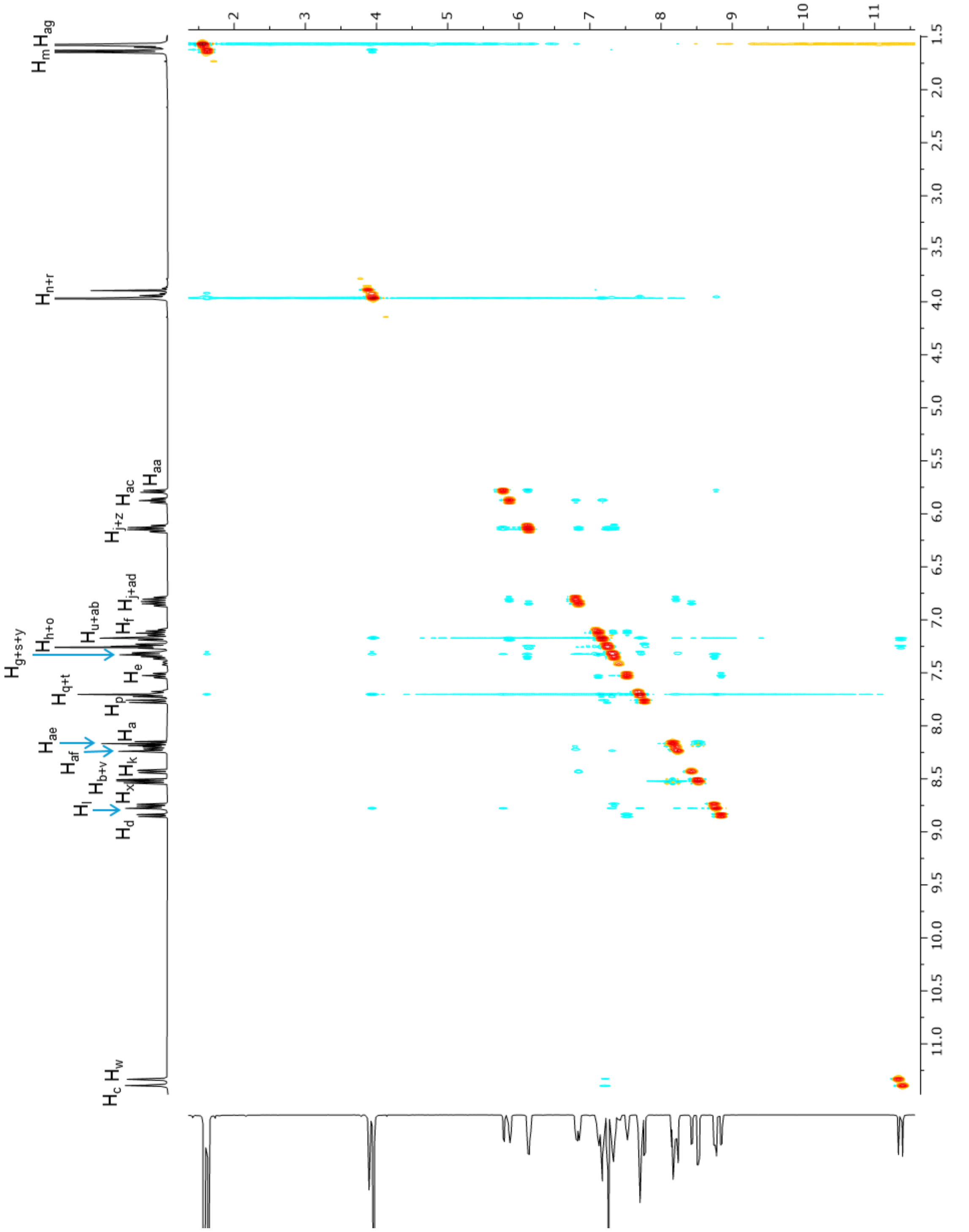

Figure S 2: Partial NOESY NMR spectrum of **4** (400 MHz, CDCl3, 298 K).

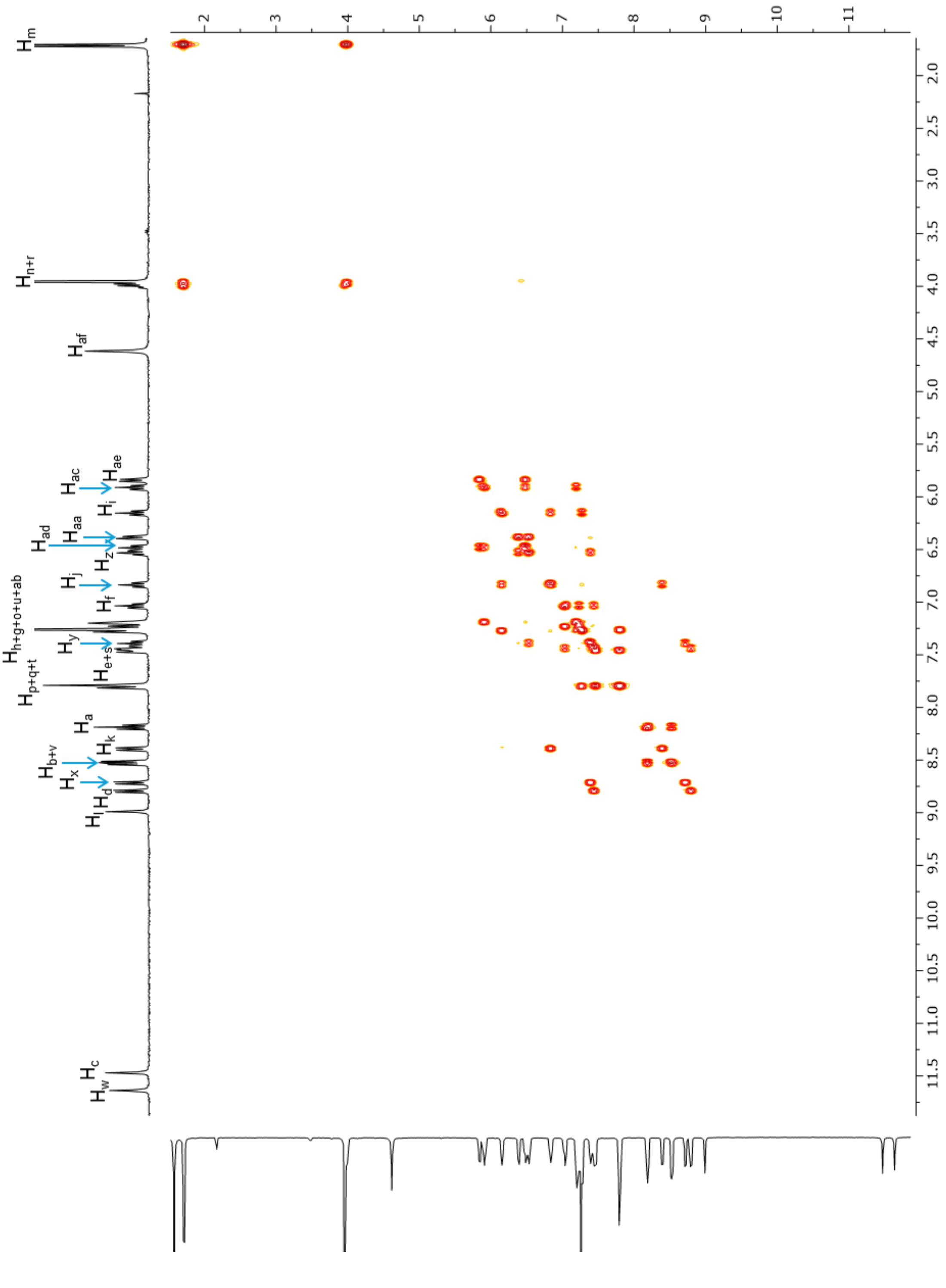

Figure S 3: Partial COSY NMR spectrum of **5** (400 MHz, CDCl3, 298 K).

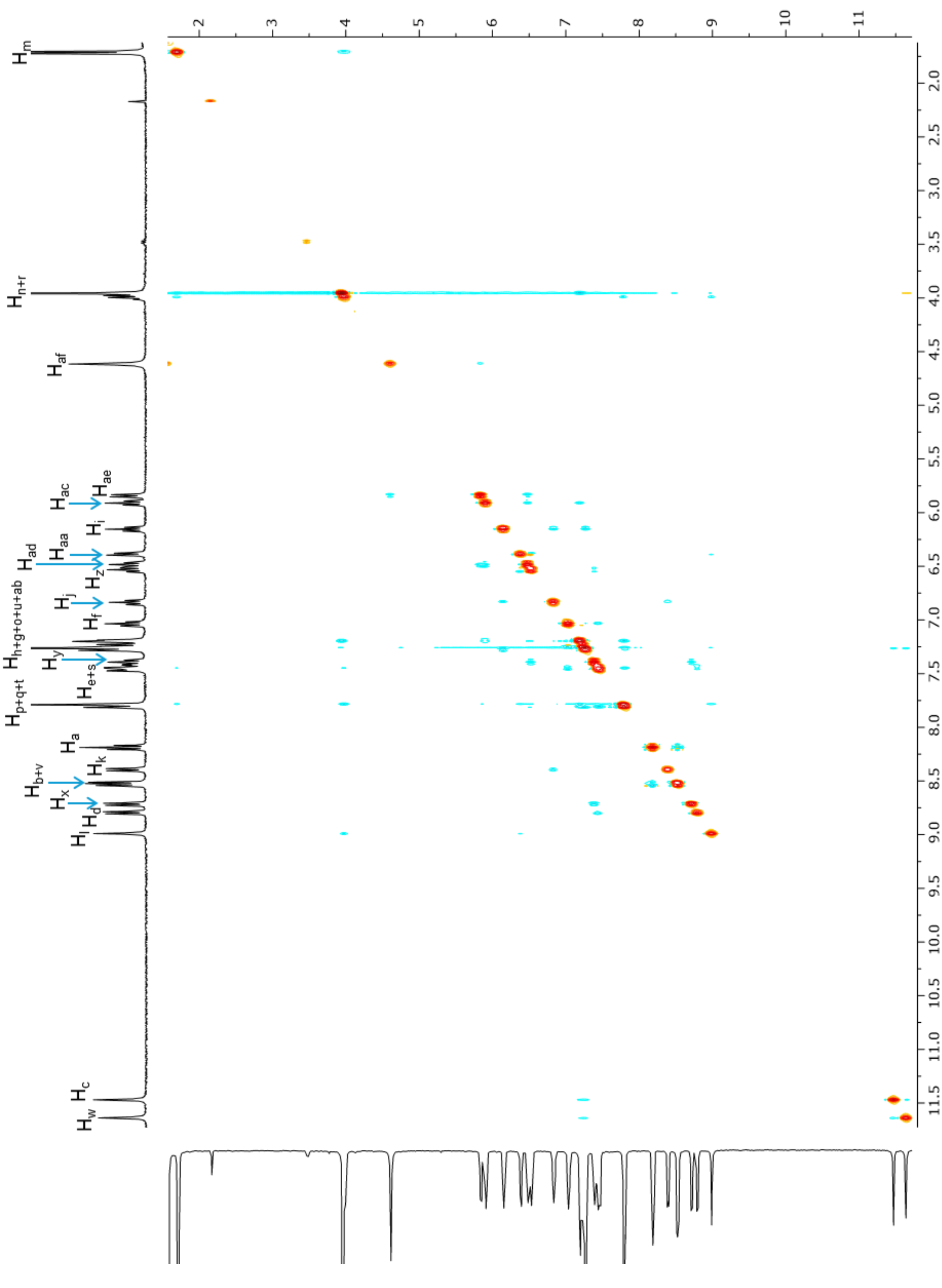

Figure S 4: Partial NOESY NMR spectrum of **5** (400 MHz, CDCl3, 298 K).

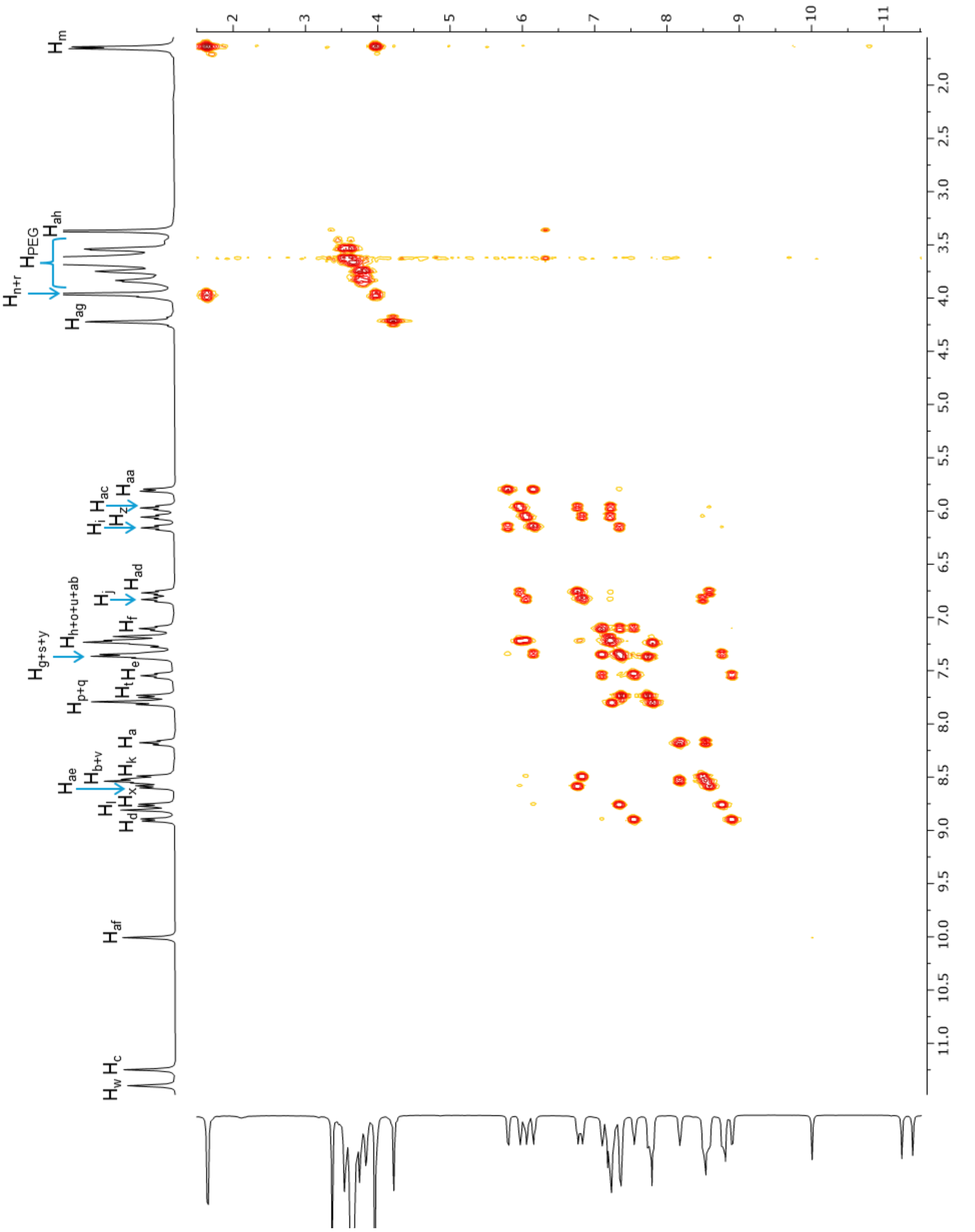

Figure S 5: Partial COSY NMR spectrum of **1** (400 MHz, CDCl3, 298 K).

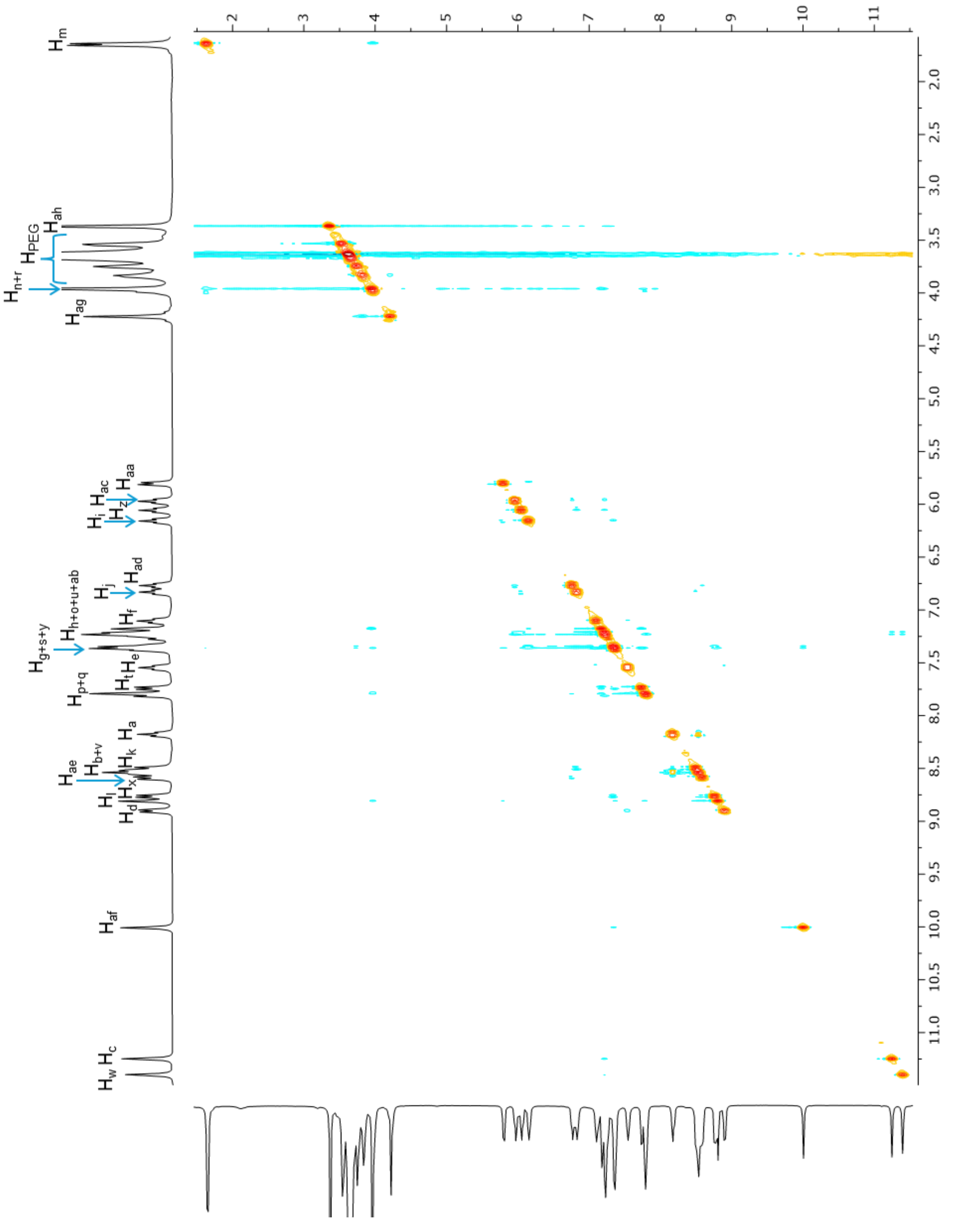

Figure S 6: Partial NOESY NMR spectrum of **1** (400 MHz, CDCl3, 298 K).

Crystal Data and Structural Refinement

4

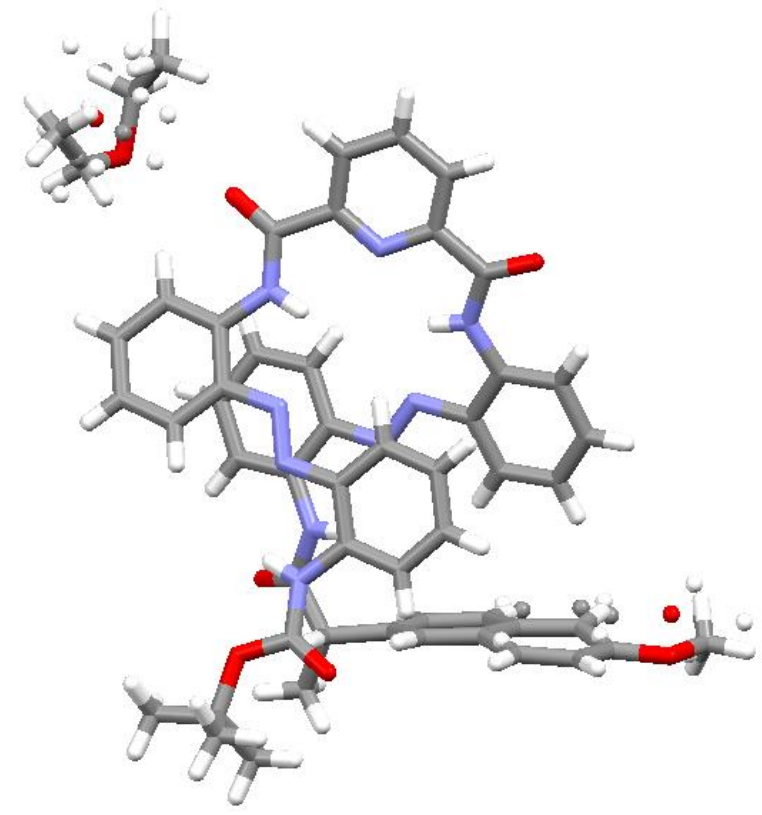

Table S1 **Crystal data and structure refinement for 4.**

Identification code	**4**
Empirical formula	$C_{54}H_{55}N_9O_7$
Formula weight	942.07
Temperature/K	99.99(10)
Crystal system	Monoclinic
Space group	C_2
a/Å	38.7699(3)
b/Å	10.22310(10)
c/Å	12.50090(10)
α/°	90
β/°	94.6670(10)
γ/°	90
Volume/Å^3	4938.29(7)
Z	4
ρcalcg/cm^3	1.267
μ/mm^{-1}	0.695
F(000)	1992.0
Crystal size/mm^3	0.184 × 0.055 × 0.034
Radiation	Cu Kα (λ = 1.54184)
2Θ range for data collection/°	4.574 to 156.646
Index ranges	$-48 \le h \le 49$, $-12 \le k \le 9$, $-15 \le l \le 15$
Reflections collected	52305
Independent reflections	8990 [R_{int} = 0.0373, R_{sigma} = 0.0271]
Data/restraints/parameters	8990/314/741
Goodness-of-fit on F^2	1.049
Final R indexes [I>=2σ (I)]	R_1 = 0.0491, wR_2 = 0.1343
Final R indexes [all data]	R_1 = 0.0517, wR_2 = 0.1367
Largest diff. peak/hole / e Å^{-3}	0.74/-0.38

Solid-State Analysis

a) Solid State Analysis of 4

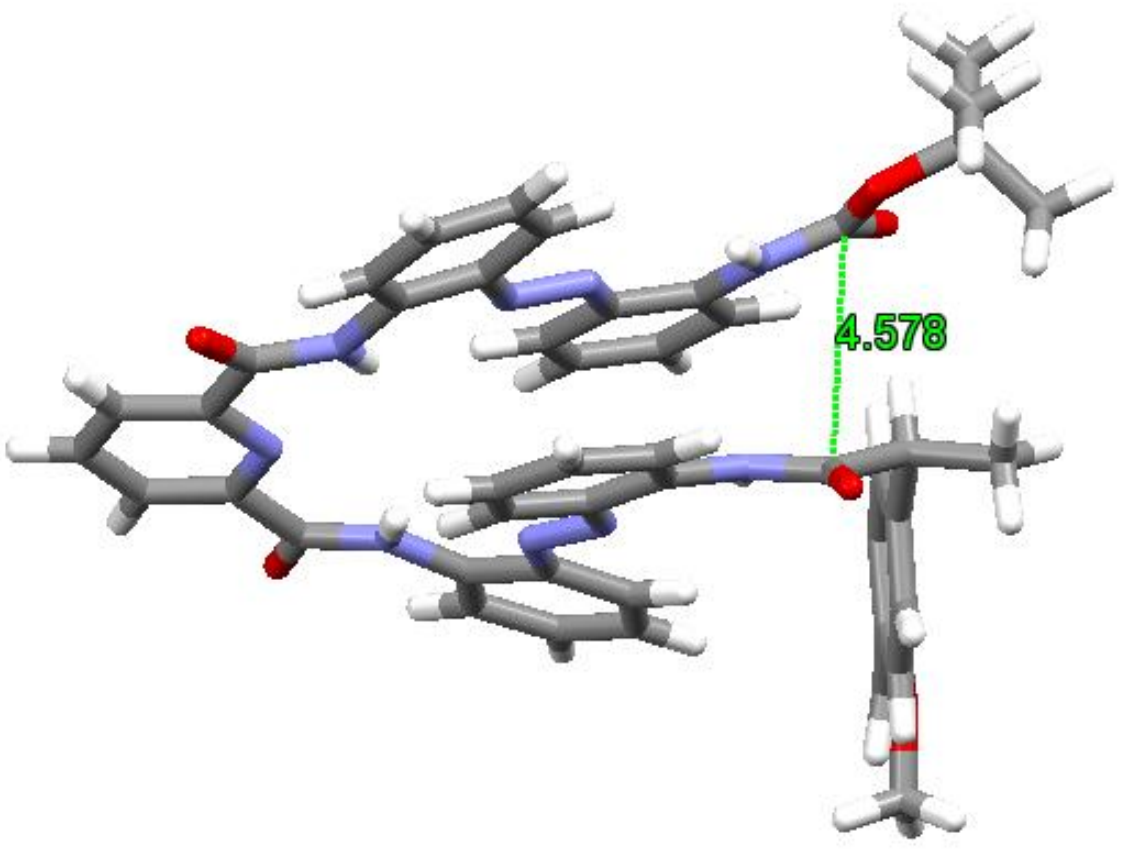

Figure S7. Solid state analysis of one of the molecules in the unit cell of **4**, showing helical pitch (distance between N atoms at each terminus).

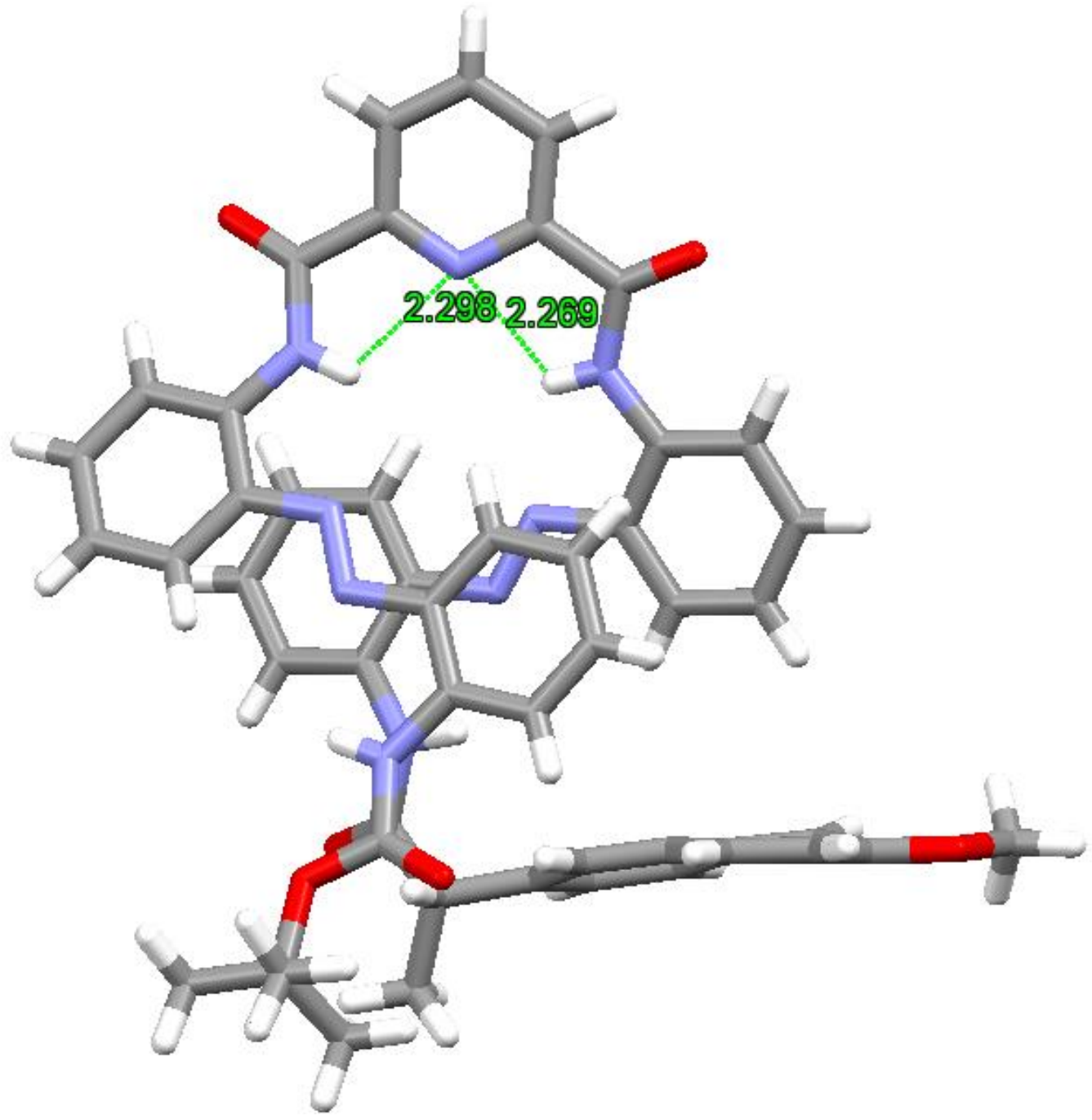

Figure S8. Solid state analysis of one molecule of **4** in the unit cell, highlighting the presence of bifurcated[6] intramolecular N-H···N hydrogen bonding interactions[6] involving the N atom of the pyridine and the two NH's of the adjacent amide bonds leading to the *syn*-*syn* conformation about the central pyridinecarboxamide unit.[7]

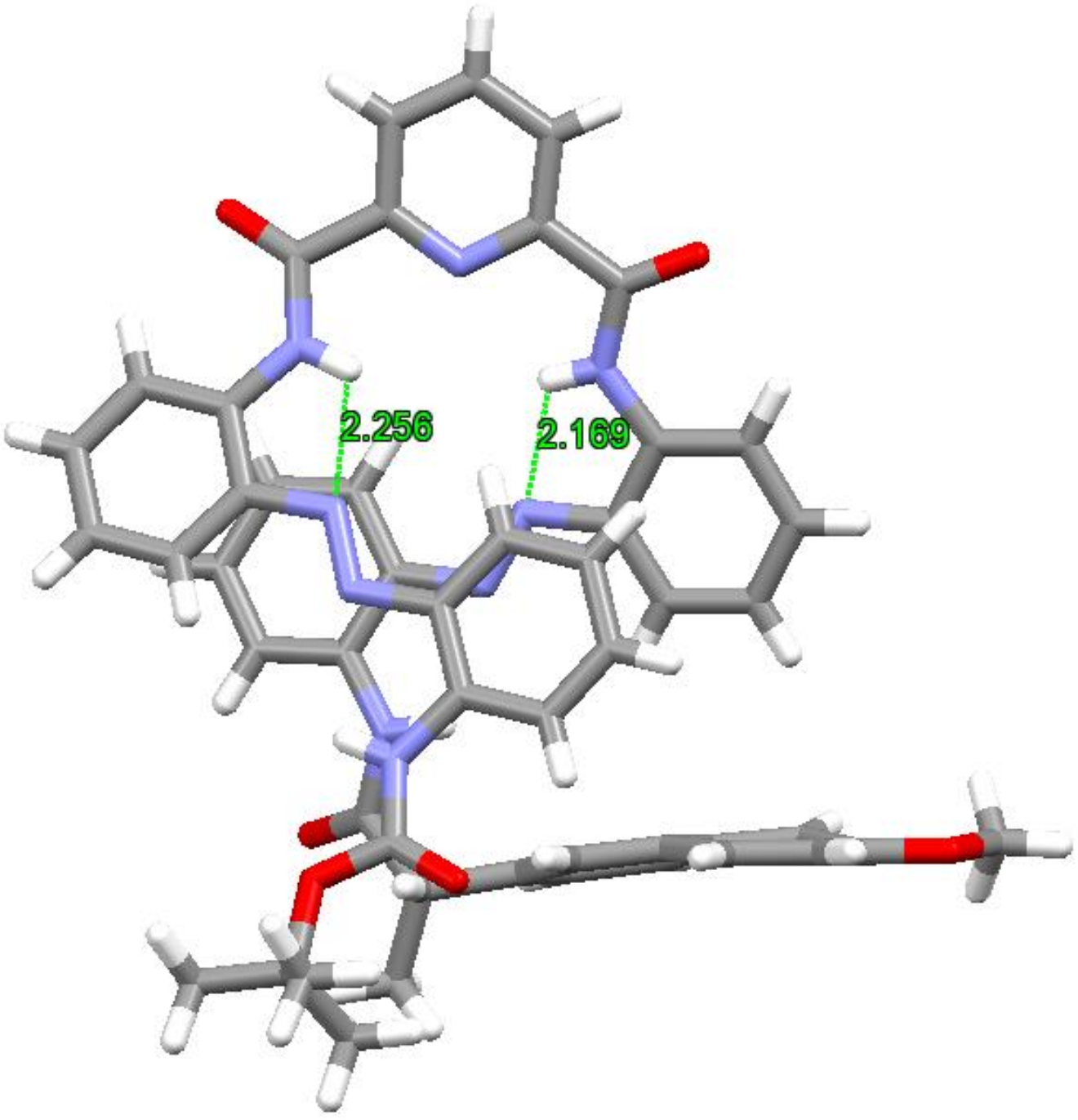

Figure S9. Solid state analysis of one molecule in the unit cell of **4**, highlighting the close contacts between the pyridyl amide NHs and the adjacent azo N atoms.[8]

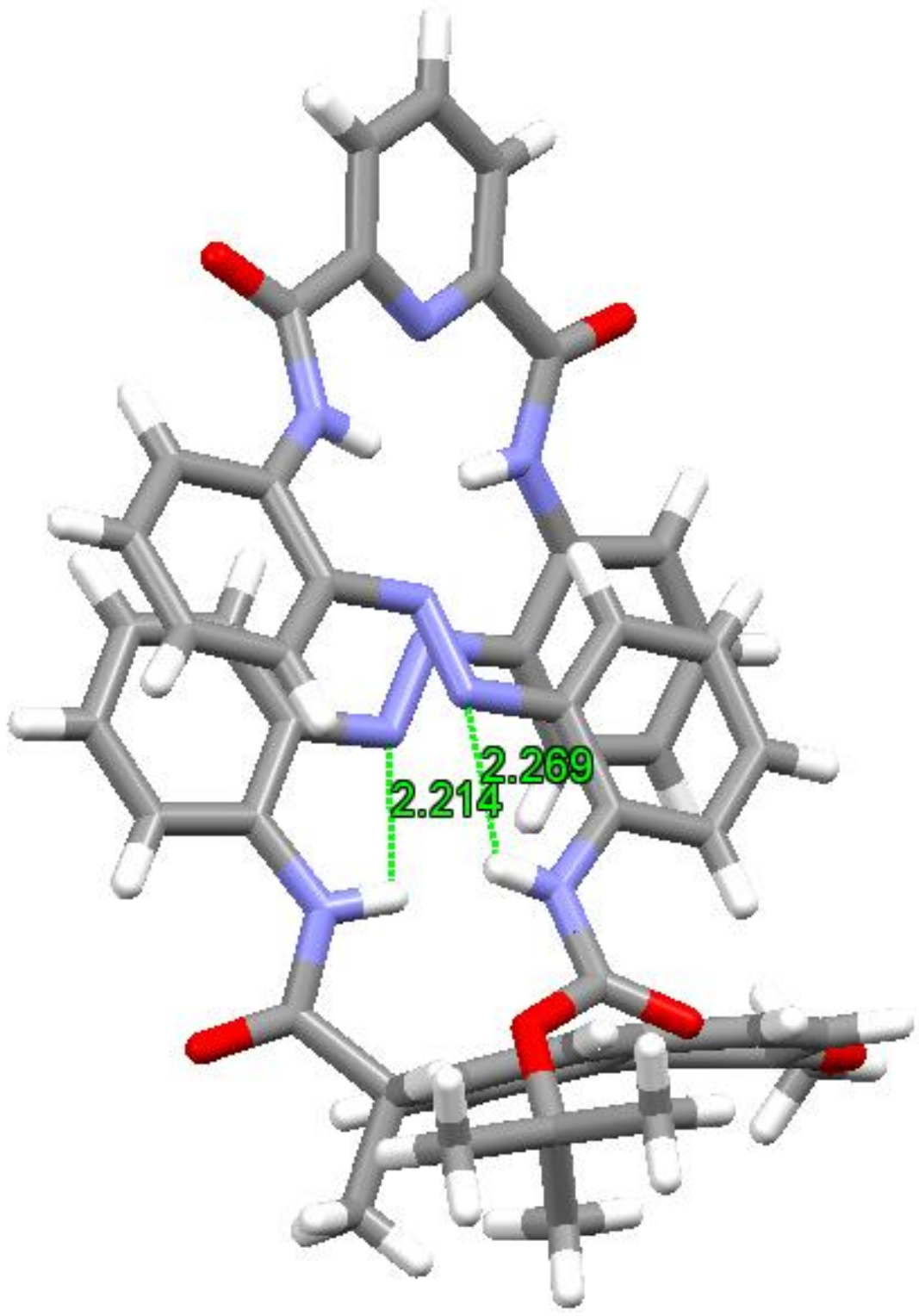

Figure S10 Solid state analysis of one molecule in the unit cell of **4**, highlighting the close contacts between the terminal acetyl amide and the adjacent azo N atoms.[8]

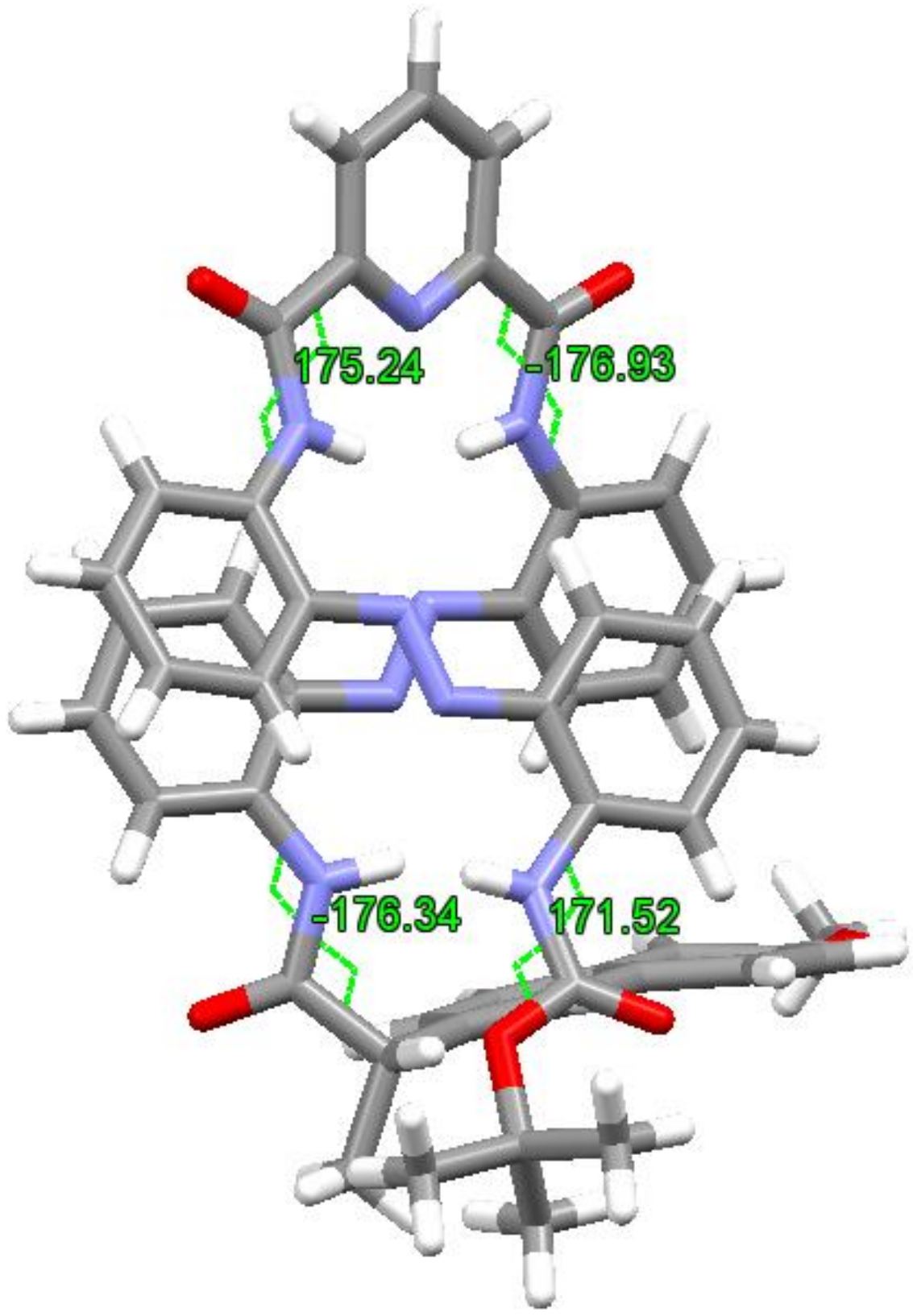

Figure S11. Solid state analysis of one molecule in the unit cell of **4**, highlighting the torsion angles of the internal peptide bonds.

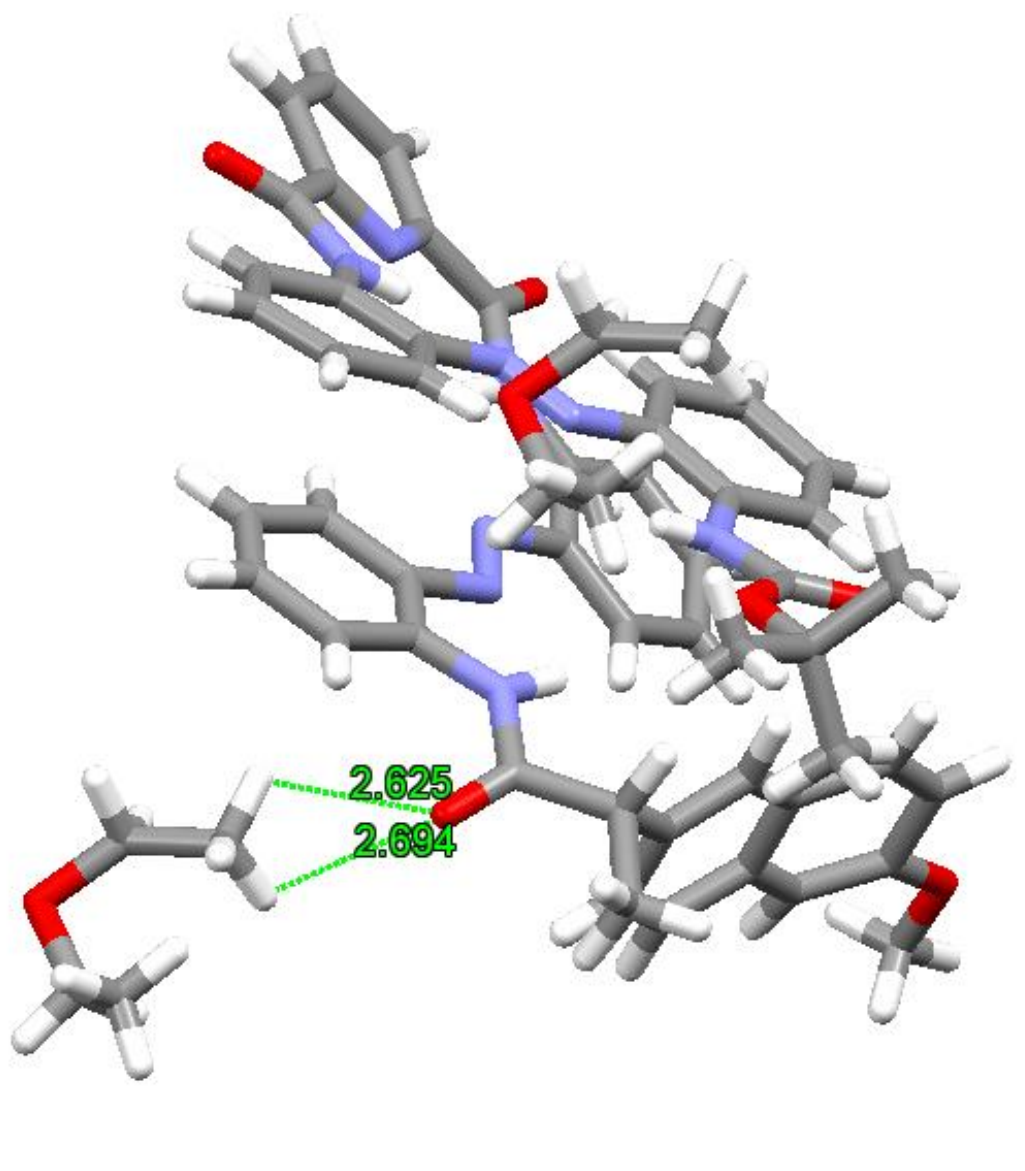

Figure S12. Solid state analysis of **4** highlighting the presence of intermolecular C-H···O hydrogen-bonding interactions[9] between the aliphatic group of the di-ethyl solvent molecule and the O atom of the terminal amide functionality in the foldamer molecule.

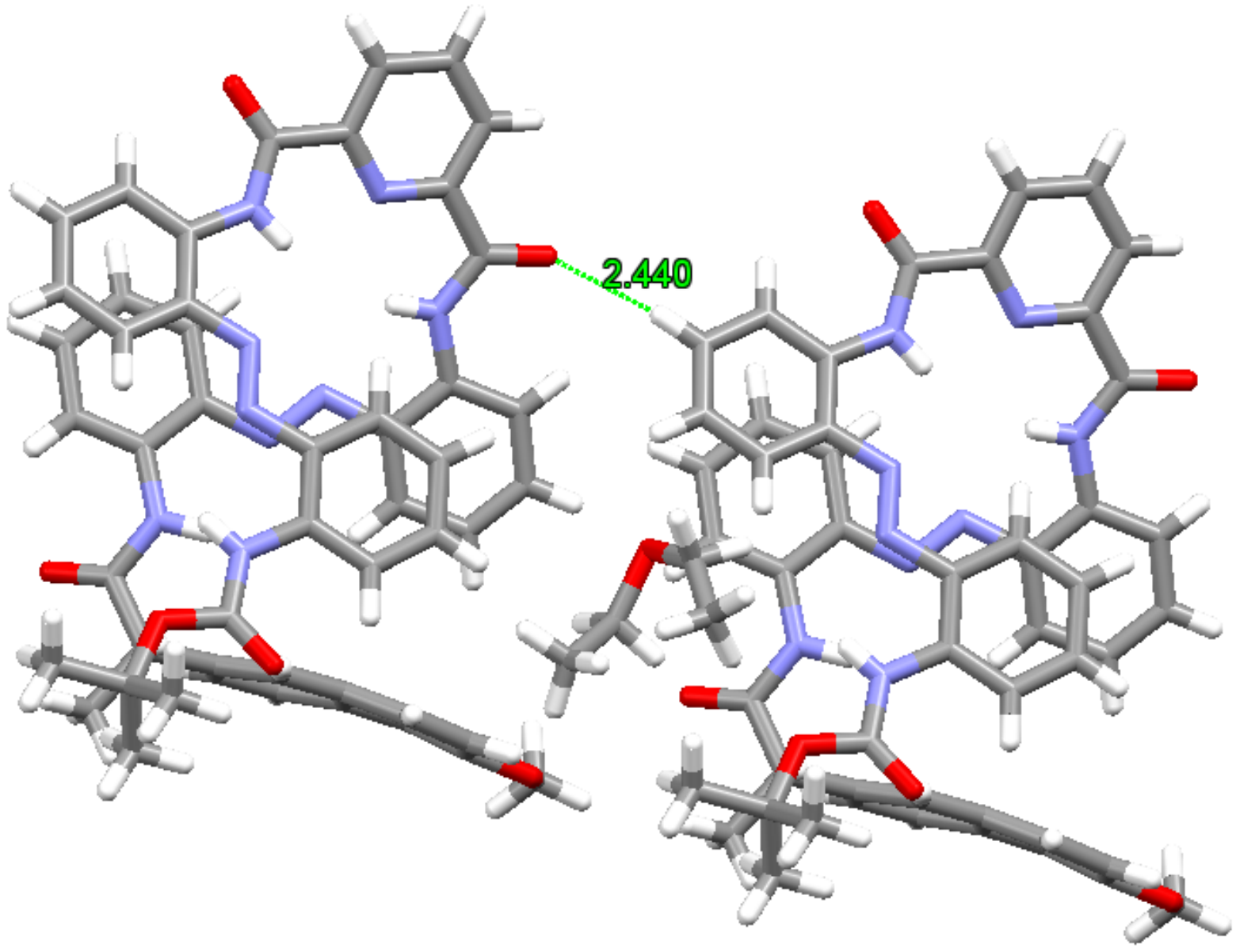

Figure S13. Solid state analysis of **4** highlighting the presence of intermolecular C-H···O hydrogen-bonding interactions[9] between aromatic protons on the one foldamer molecule and the O atoms of a carbonyl group in the carboxamide unit of an adjacent foldamer molecule.

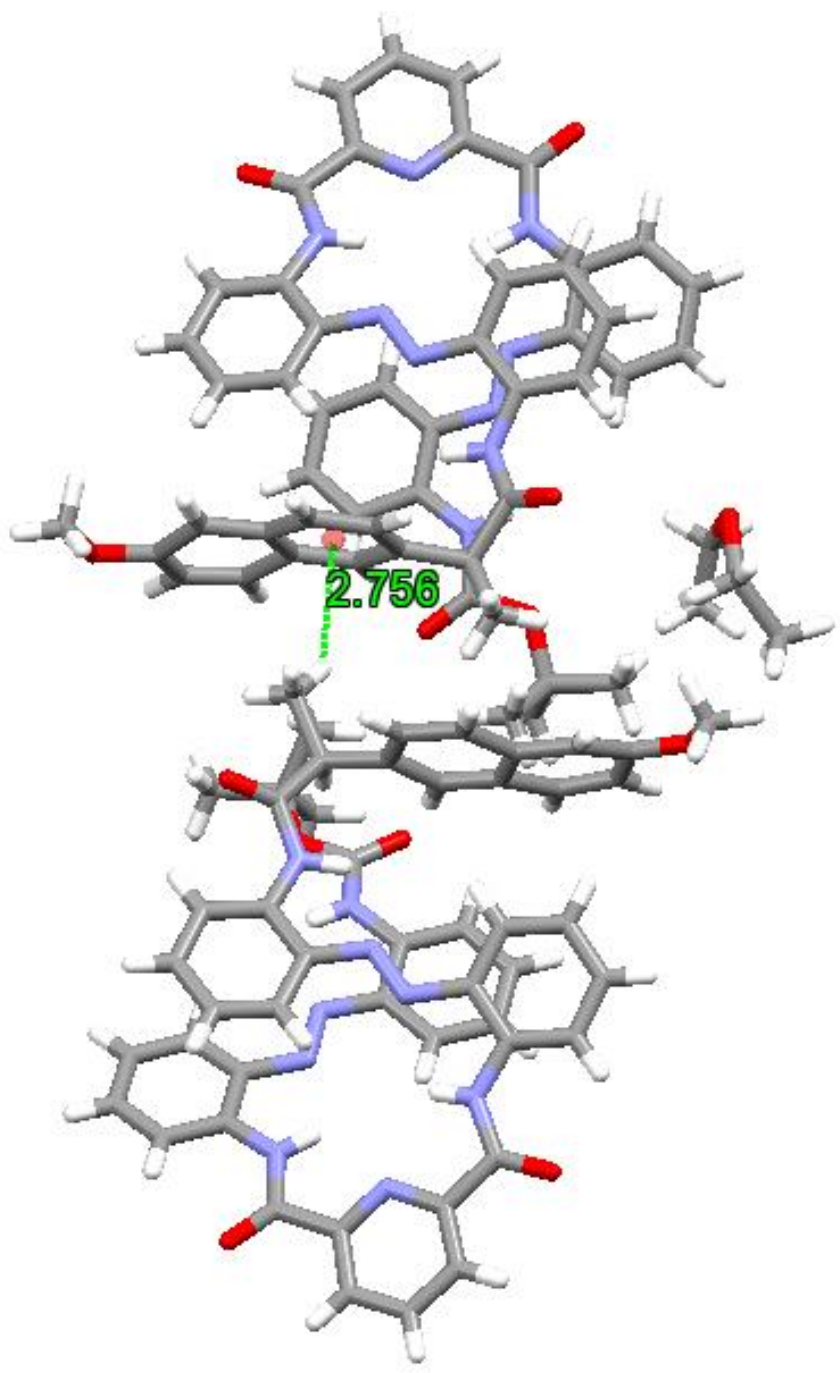

Figure S14. Solid state analysis of **4** highlighting the presence of intermolecular C-H···π stacking interactions[9] between an aliphatic proton on the terminal Boc group of one foldamer and an aromatic ring of the the naproxen group of a neighbouring foldamer molecule.

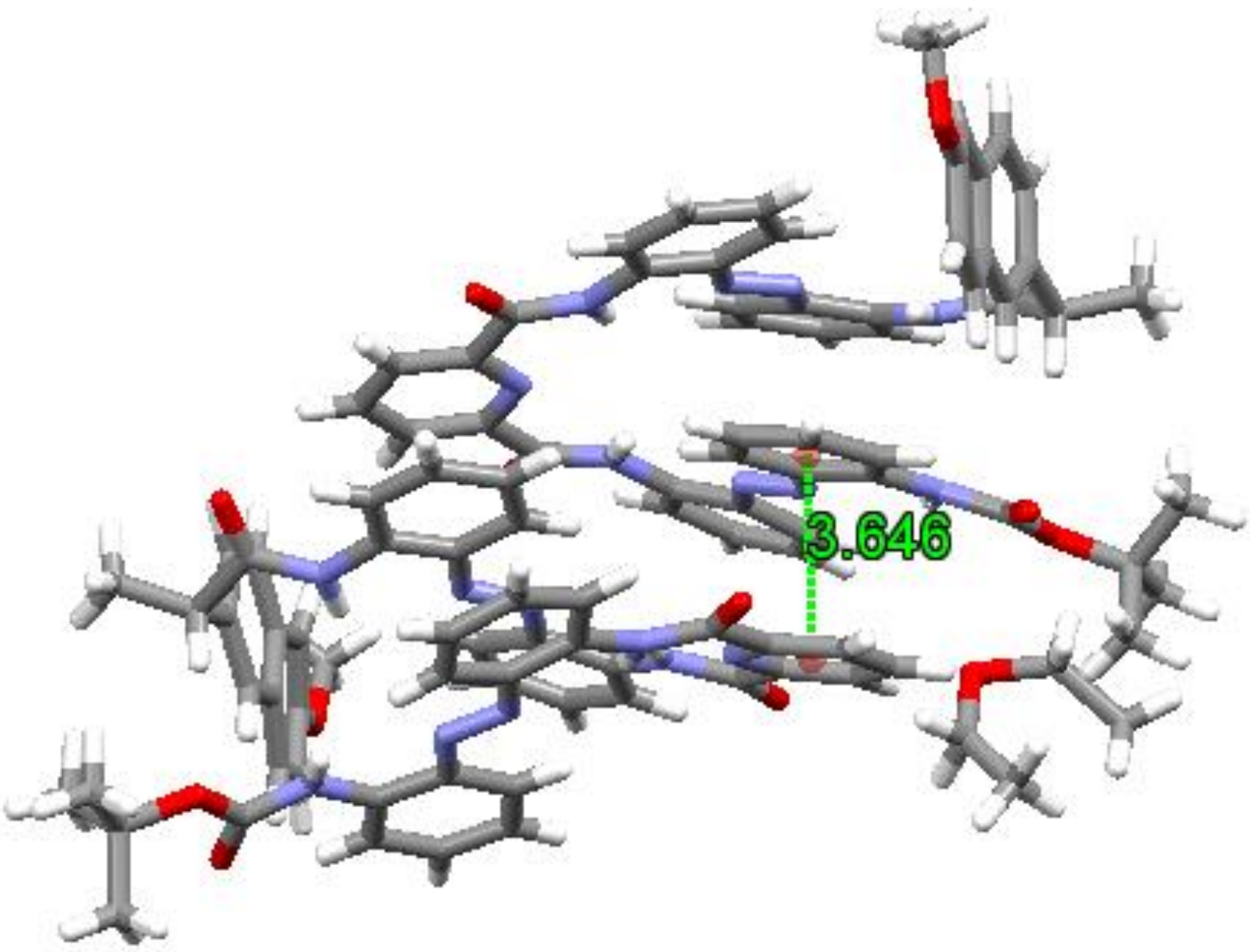

Figure S15. Solid state analysis of **4** highlighting the presence of intermolecular π-π stacking interactions between the aromatic rings of adjacent foldamer molecules.

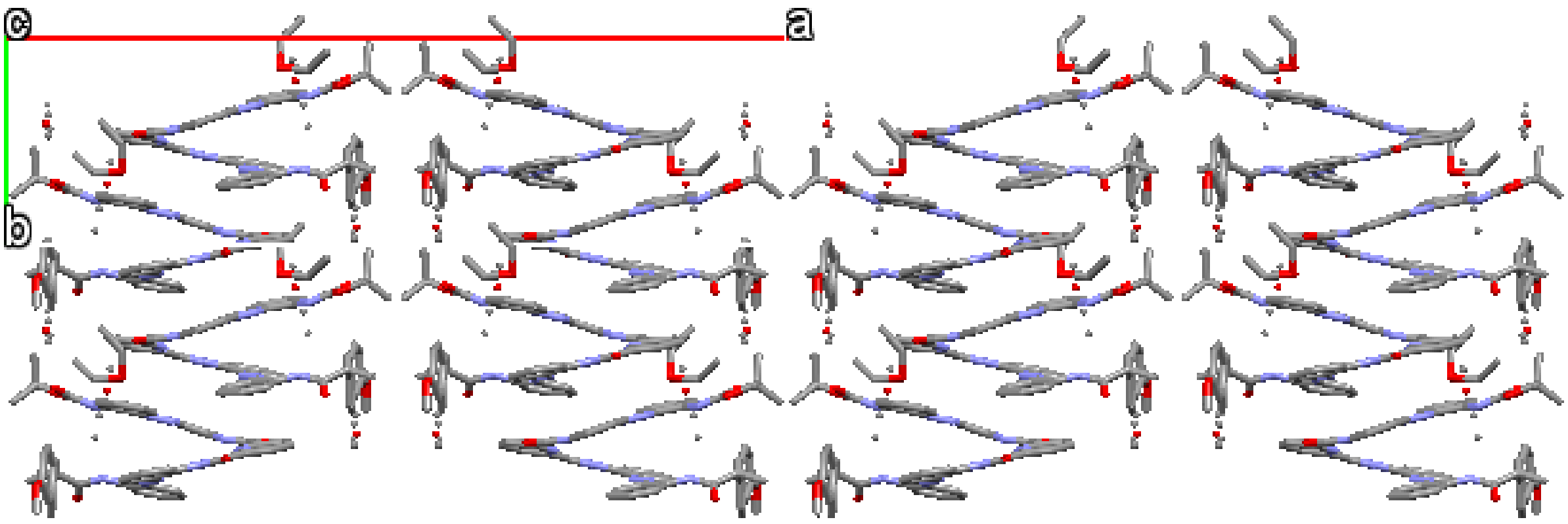

Figure S16. Solid state analysis showing the crystal packing of **4** as viewed along the *c* axis. H atoms have been removed for clarity.

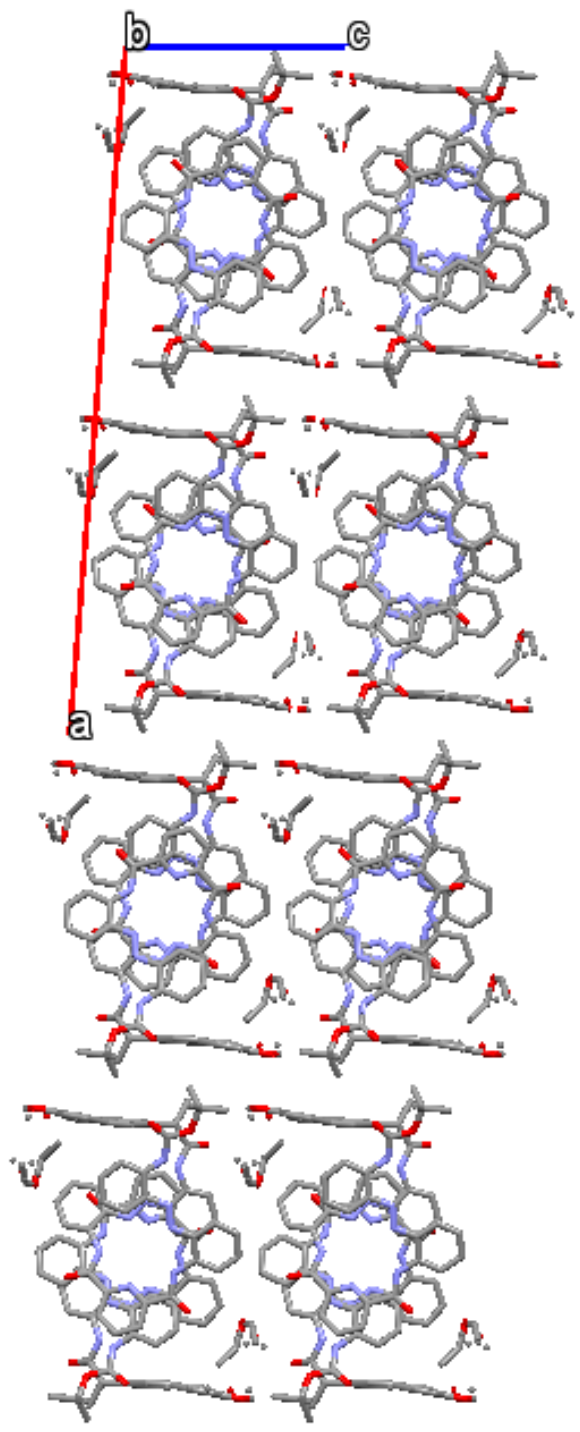

Figure S17. Solid state analysis showing the crystal packing of **4** as viewed along the *b* axis. H atoms have been removed for clarity.

Circular Dichroism Spectroscopy Solvent Studies

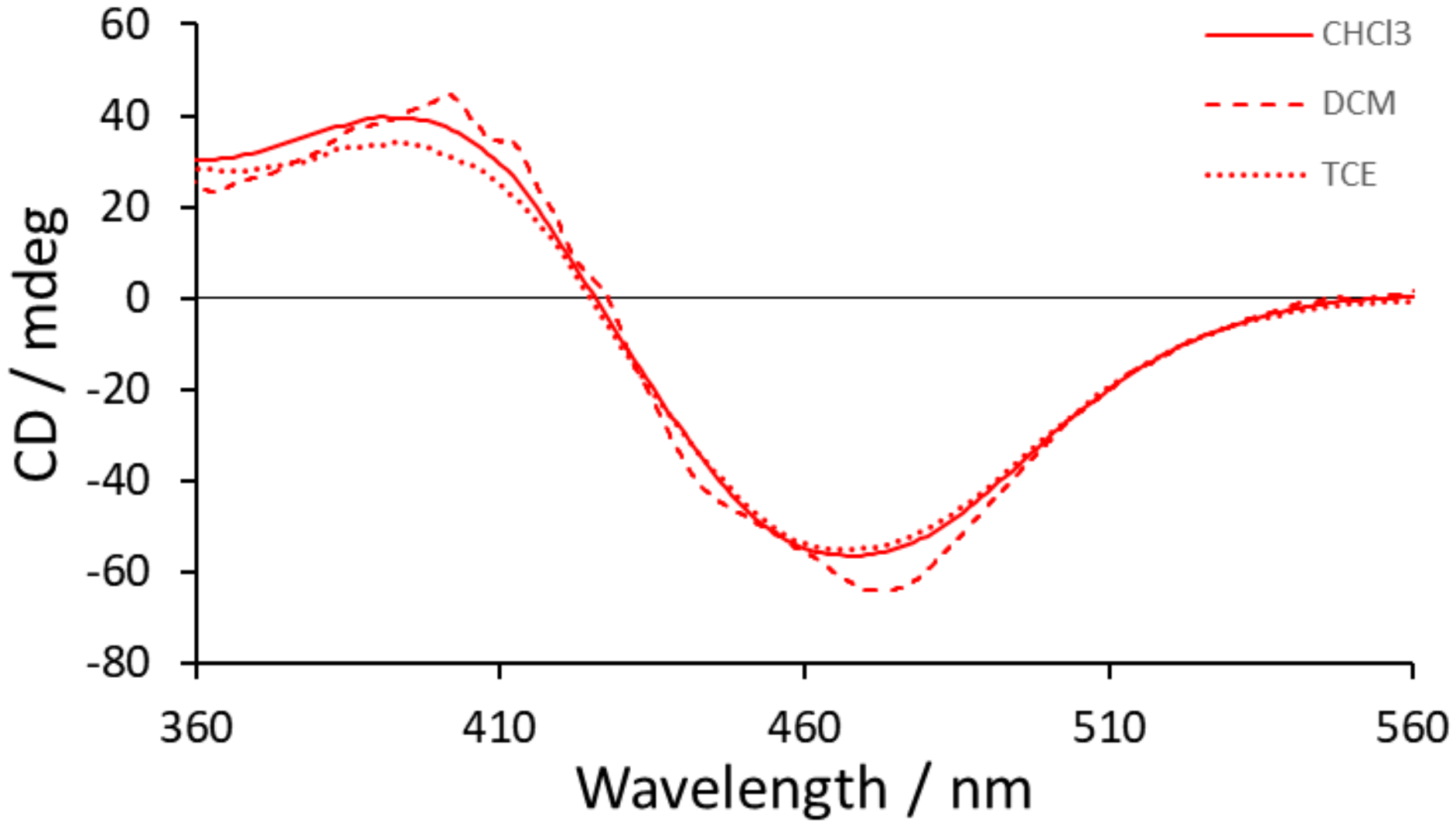

Figure S18: CD spectrum of **1** in halogenated solvents (1.5 × 10^{-4} M, 293 K).

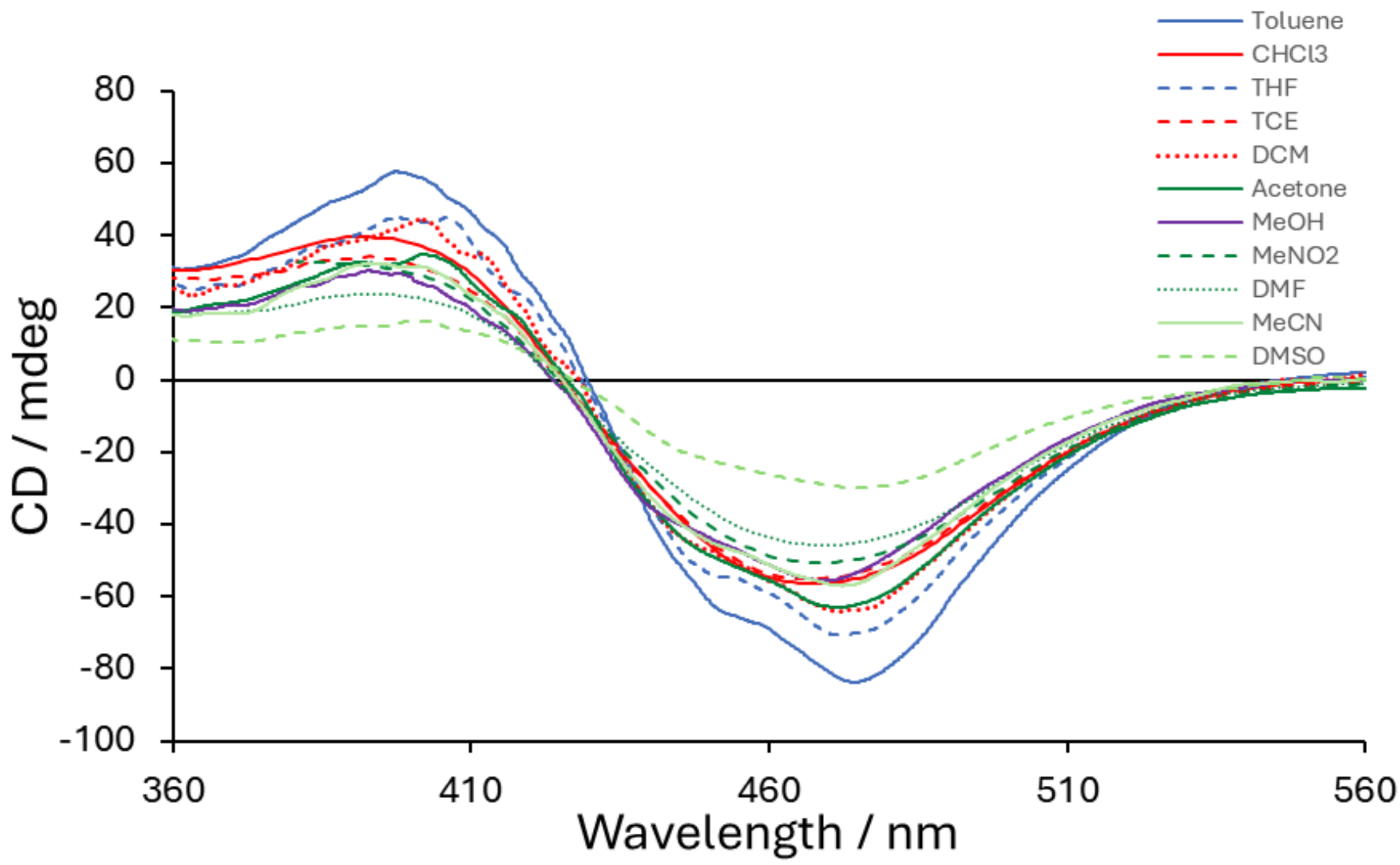

Figure S19: CD spectrum of **1** in a range of solvents (1.5 × 10^{-4} M, 293 K).

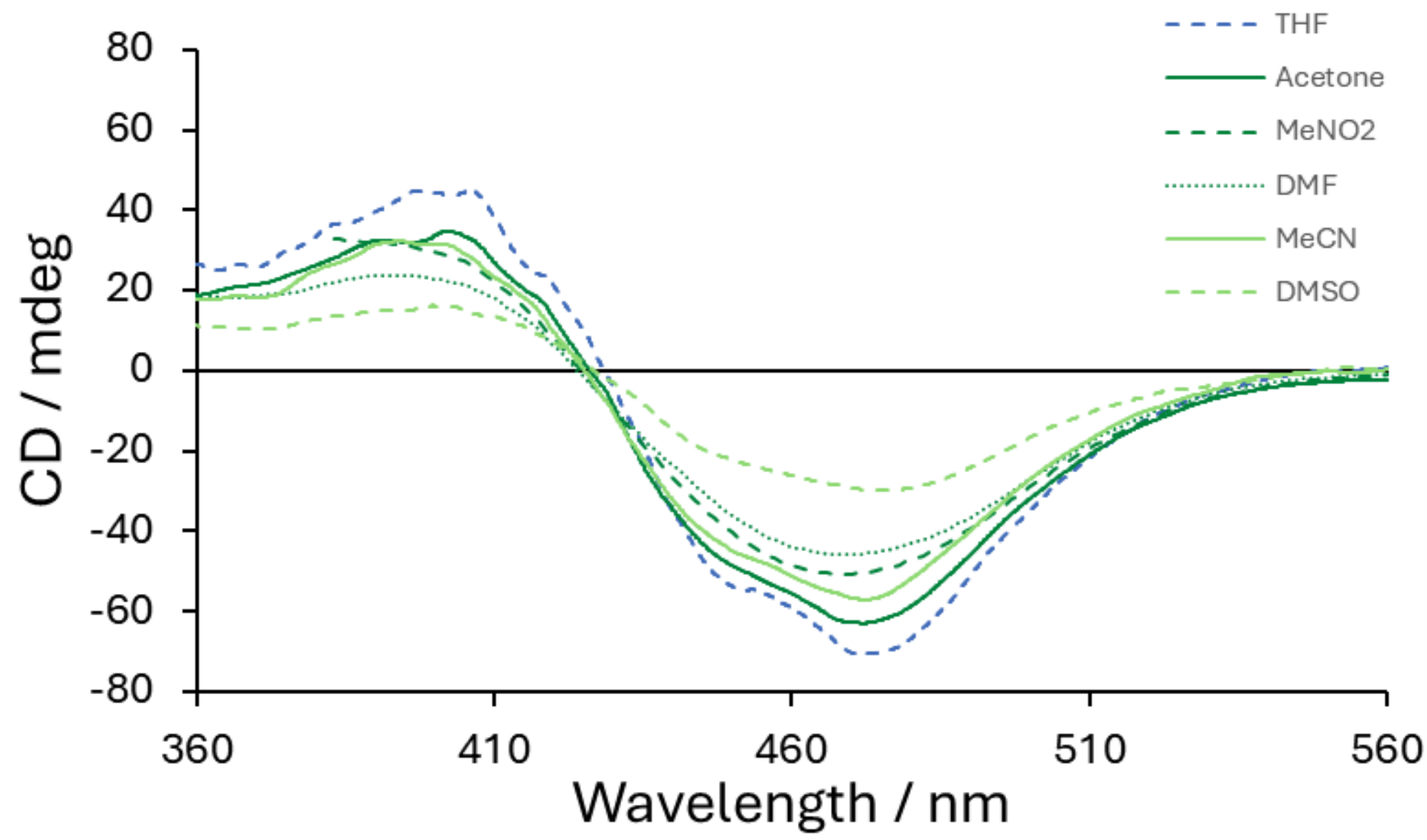

Figure S20: CD spectrum of **1** in a range of solvents (1.5×10^{-4} M, 293 K).

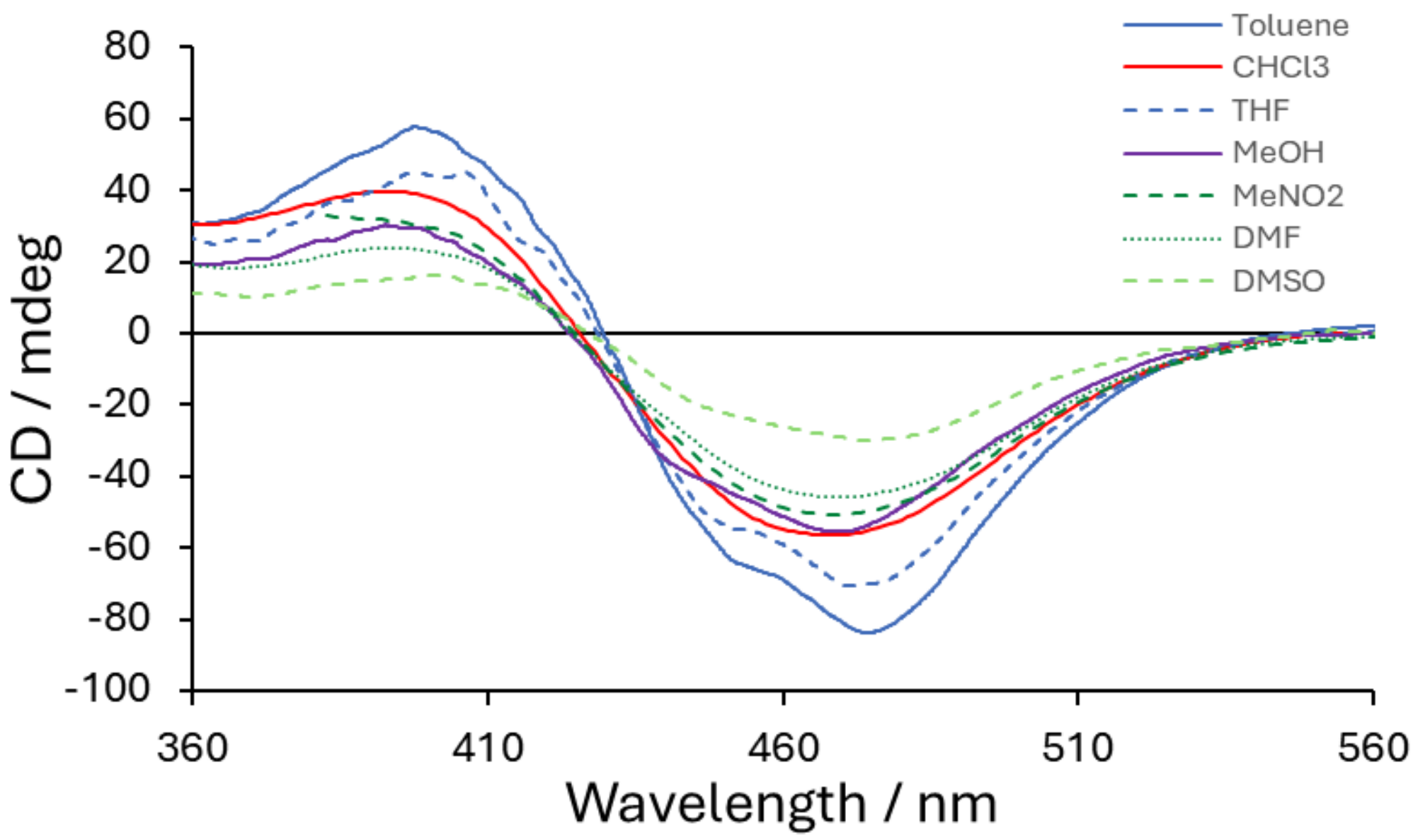

Figure S21: CD spectrum of **1** in a range of solvents (1.5×10^{-4} M, 293 K).

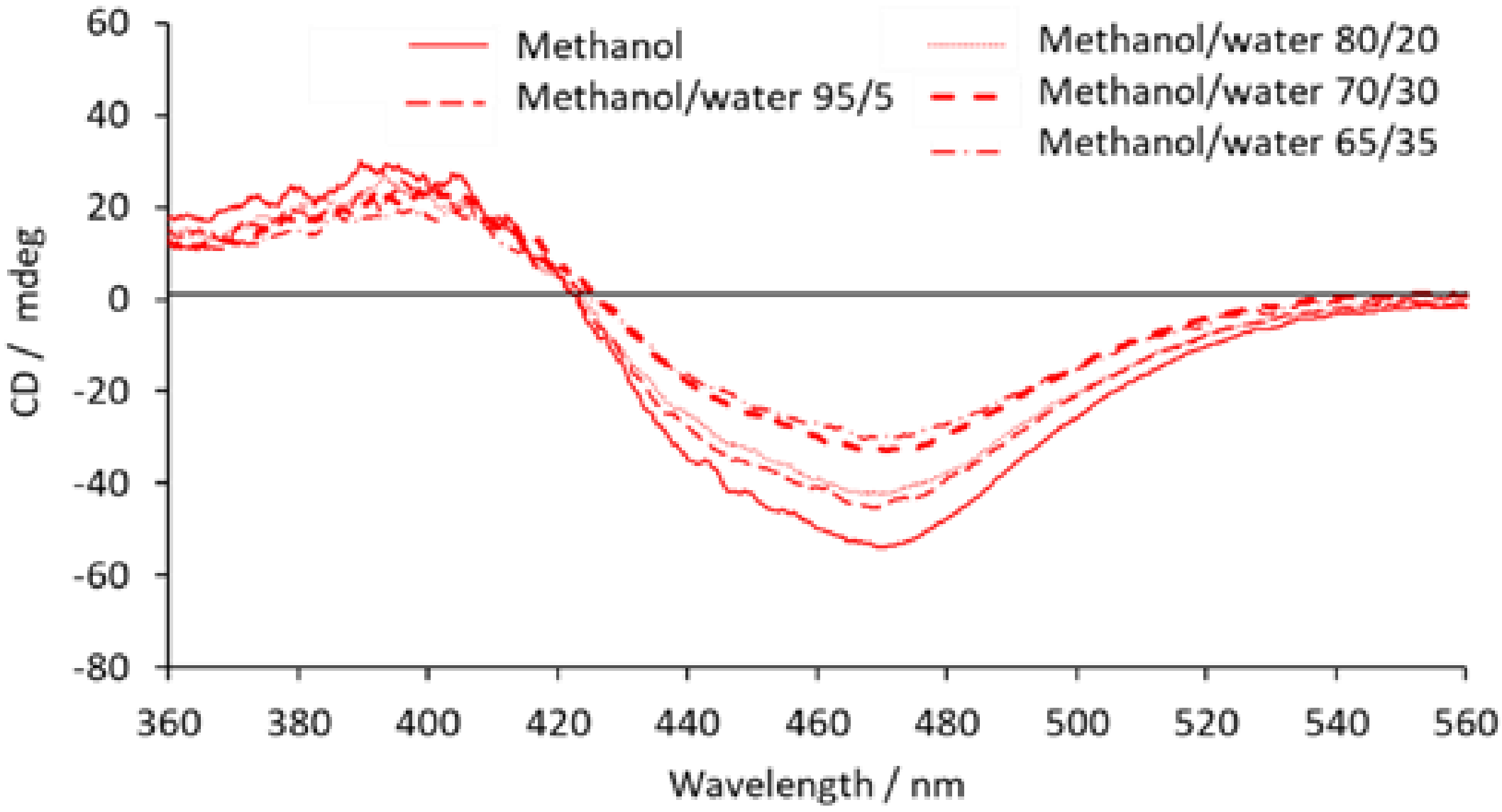

Figure S22: CD spectrum of **1** in a range of solvents (1.5 × 10^{-4} M, 293 K).

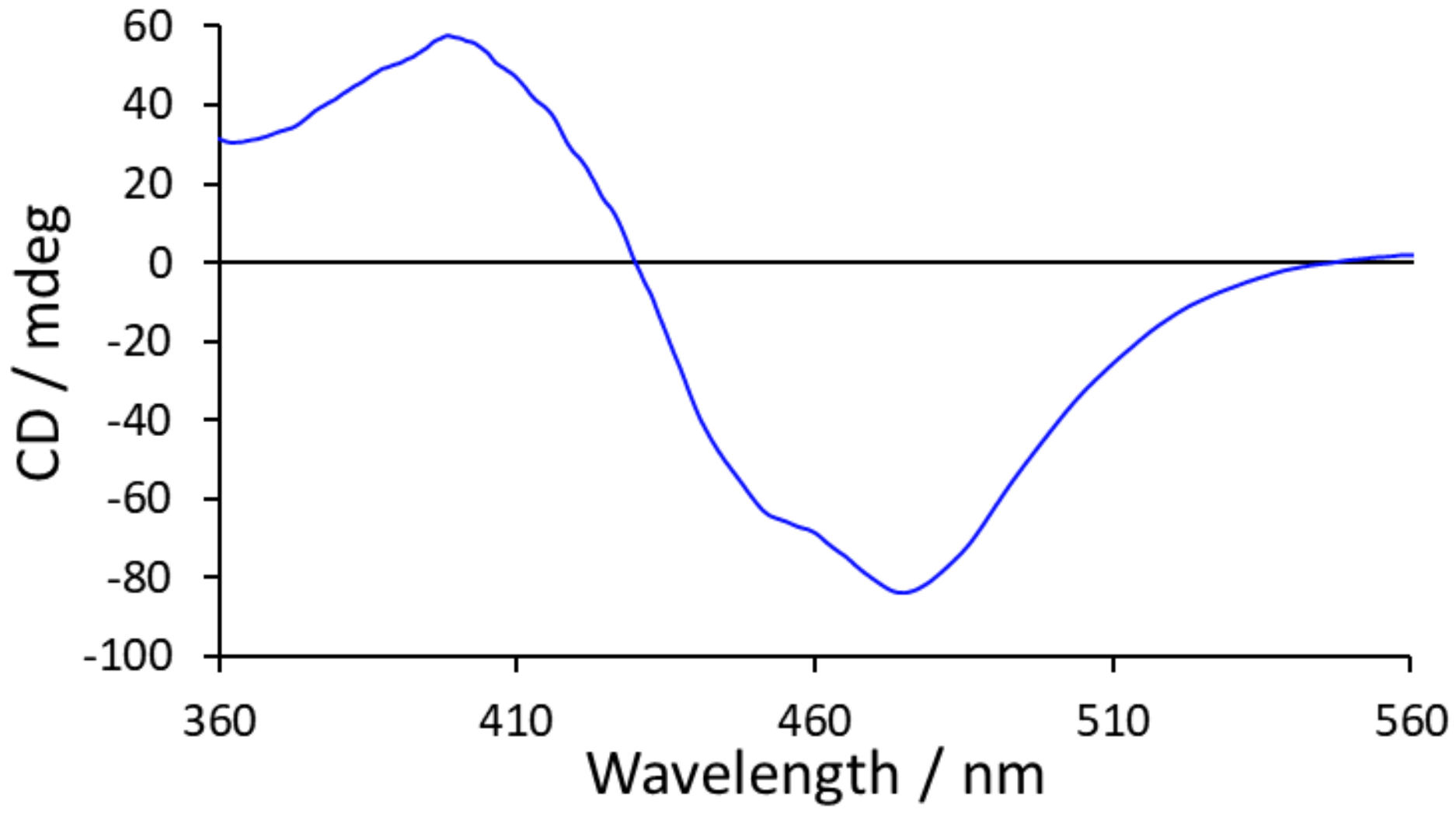

Figure S23: CD spectrum of **1** (Toluene, 1.5 × 10^{-4} M, 293 K).

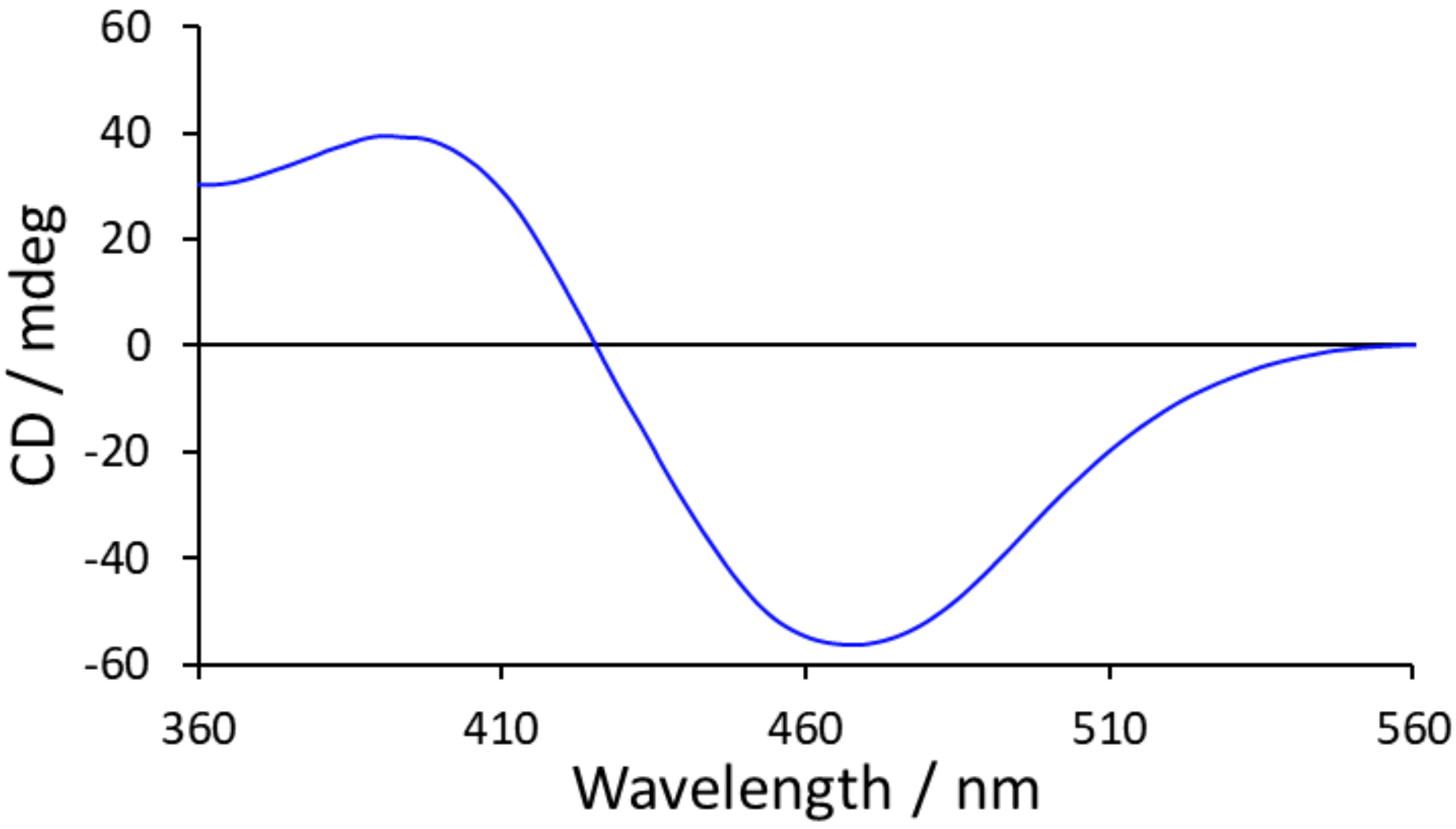

Figure S24: CD spectrum of **1** ($CHCl_3$, 1.5 × 10^{-4} M, 293 K).

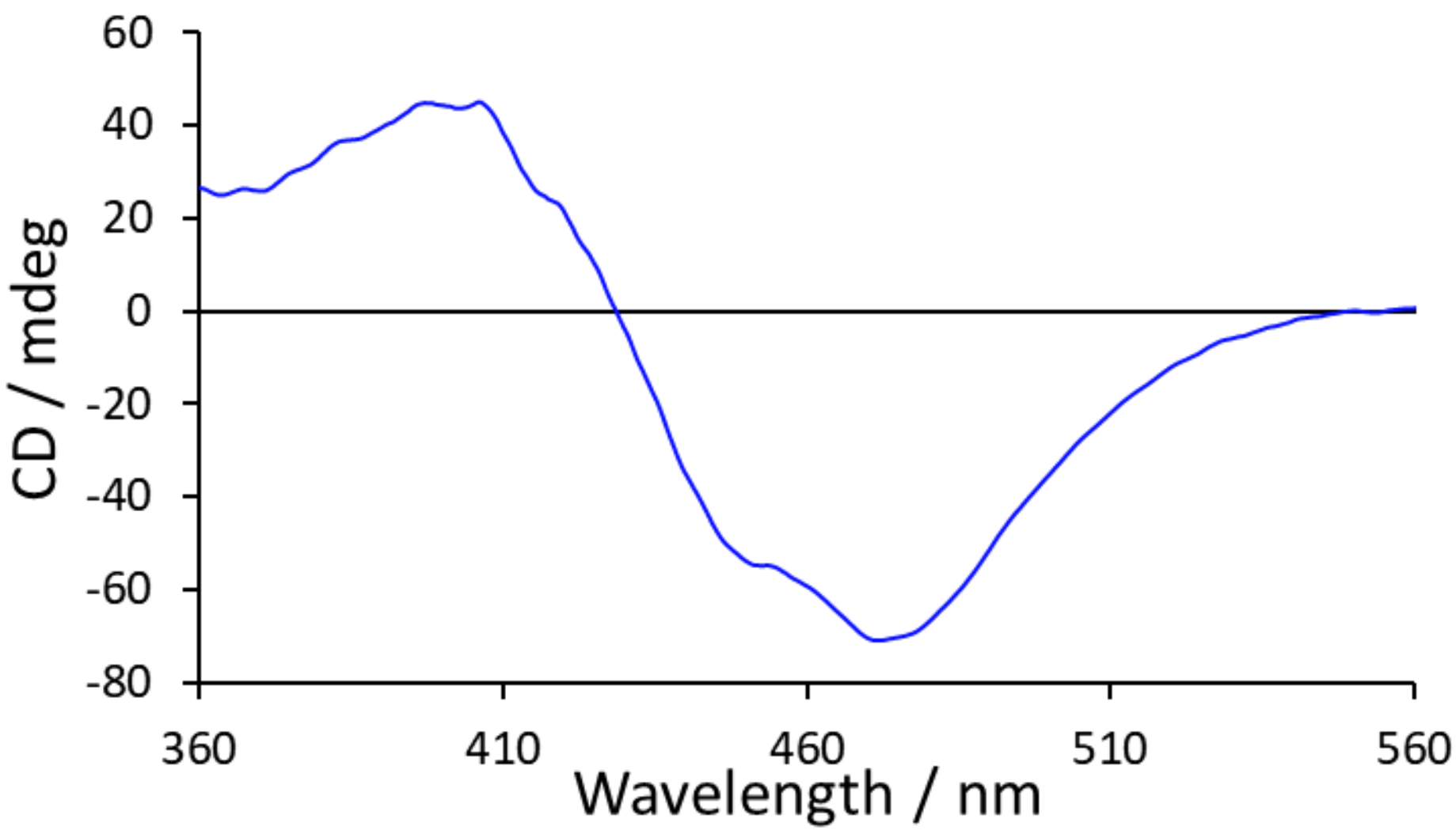

Figure S25: CD spectrum of **1** (THF, 1.5 × 10^{-4} M, 293 K).

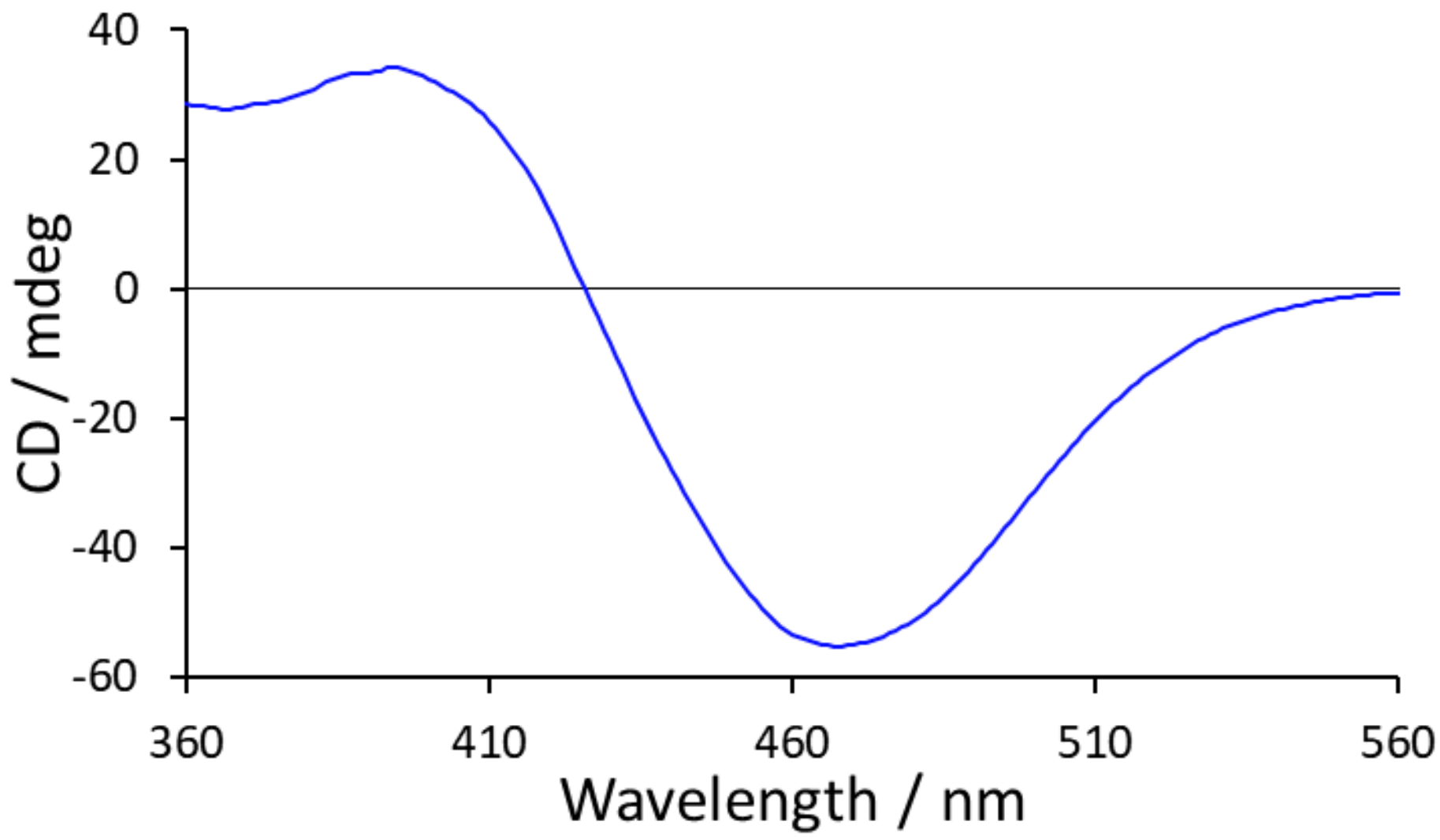

Figure S26: CD spectrum of **1** (TCE, 1.5 × 10^{-4} M, 293 K).

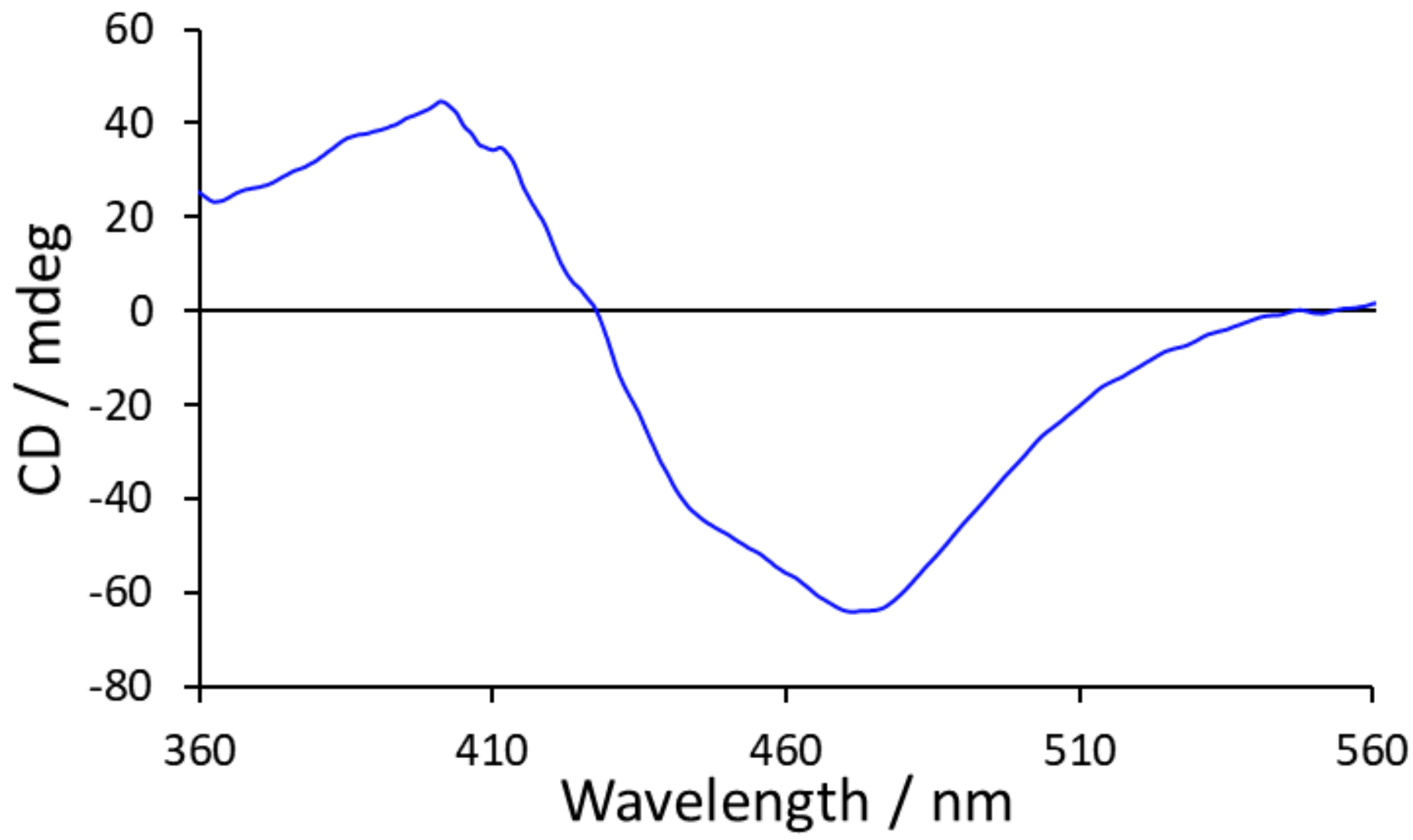

Figure S27: CD spectrum of **1** (DCM, 1.5 × 10^{-4} M, 293 K).

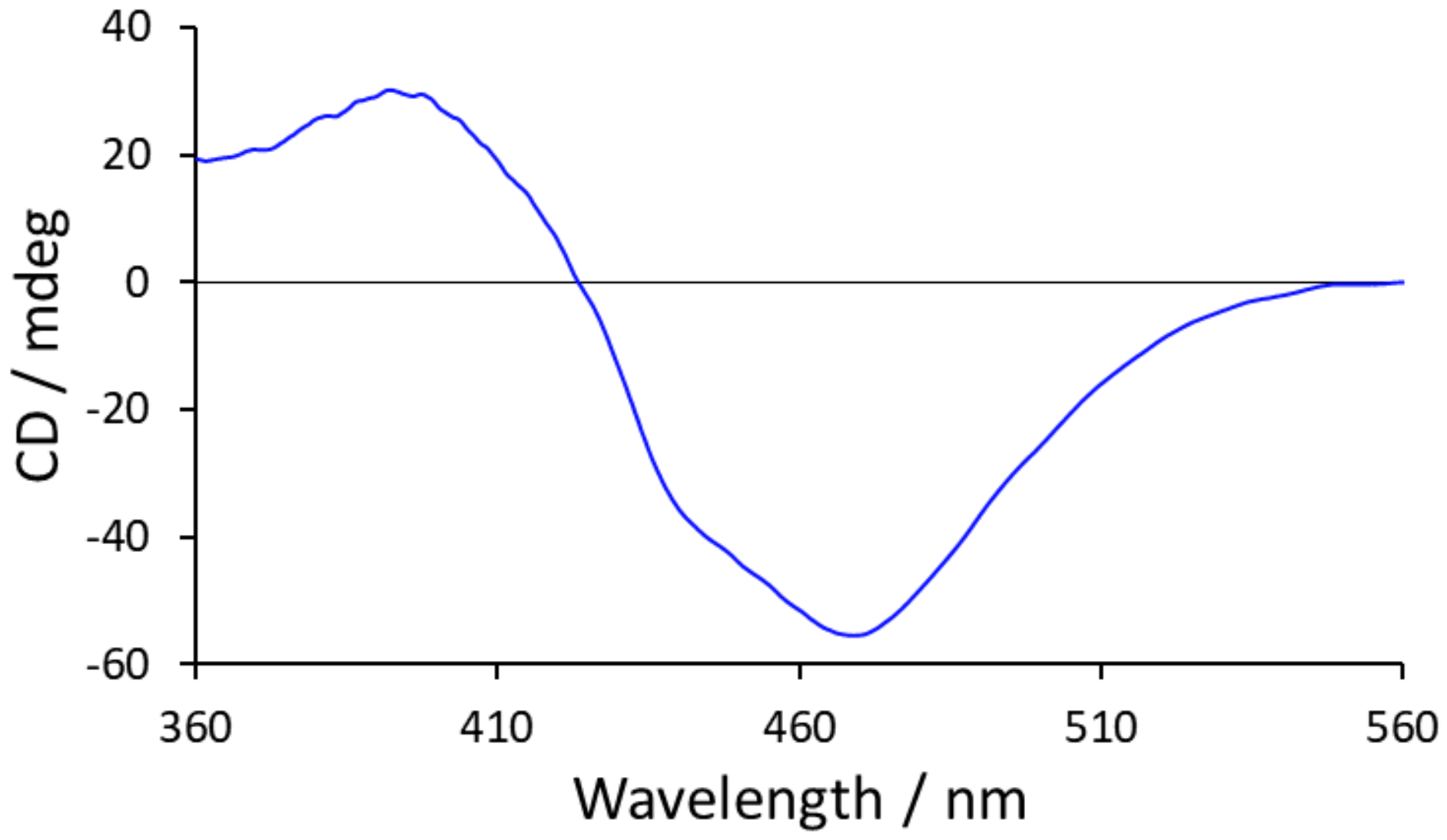

Figure S28: CD spectrum of **1** (MeOH, 1.5×10^{-4} M, 293 K).

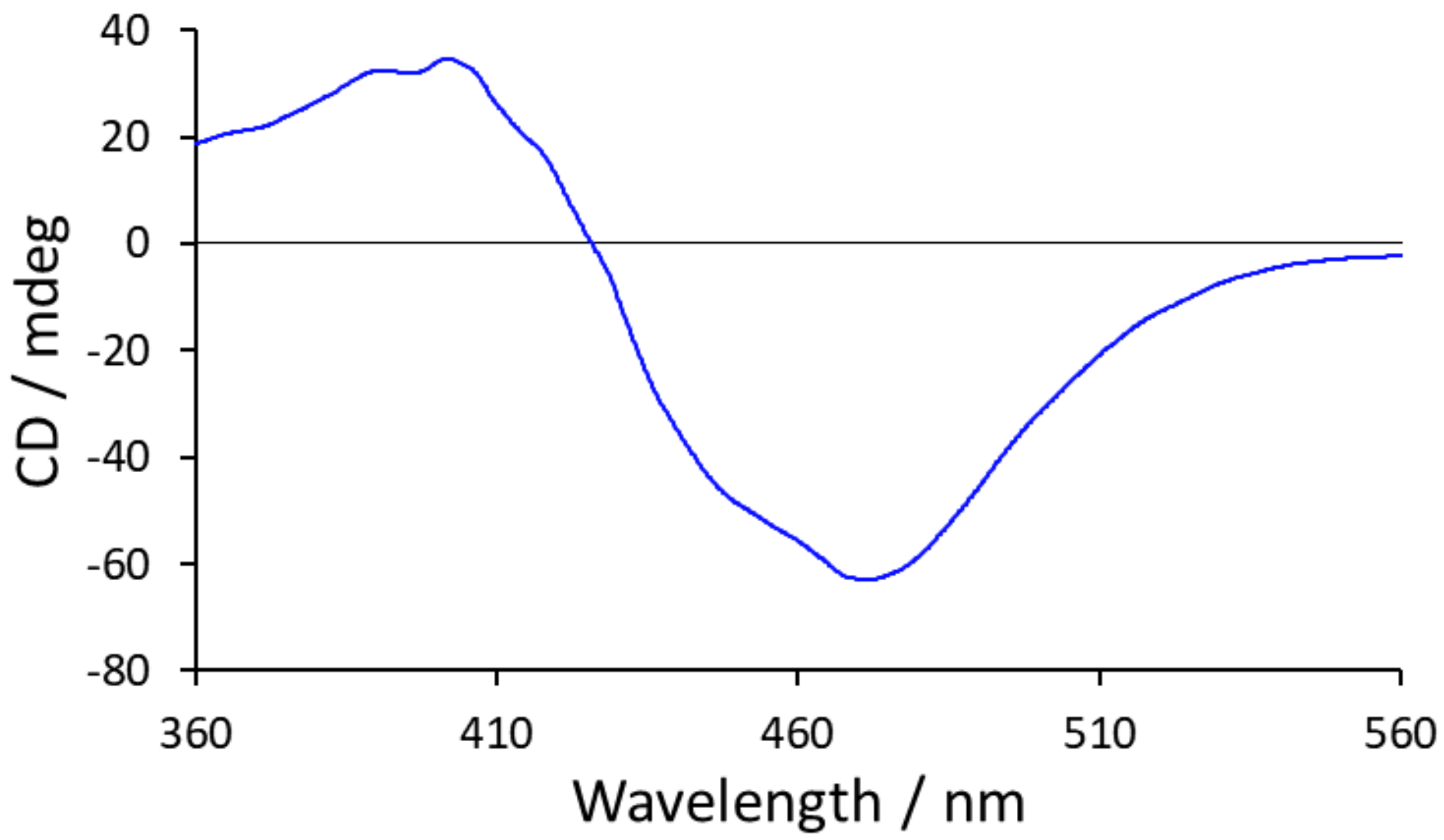

Figure S29: CD spectrum of **1** (Acetone, 1.5×10^{-4} M, 293 K).

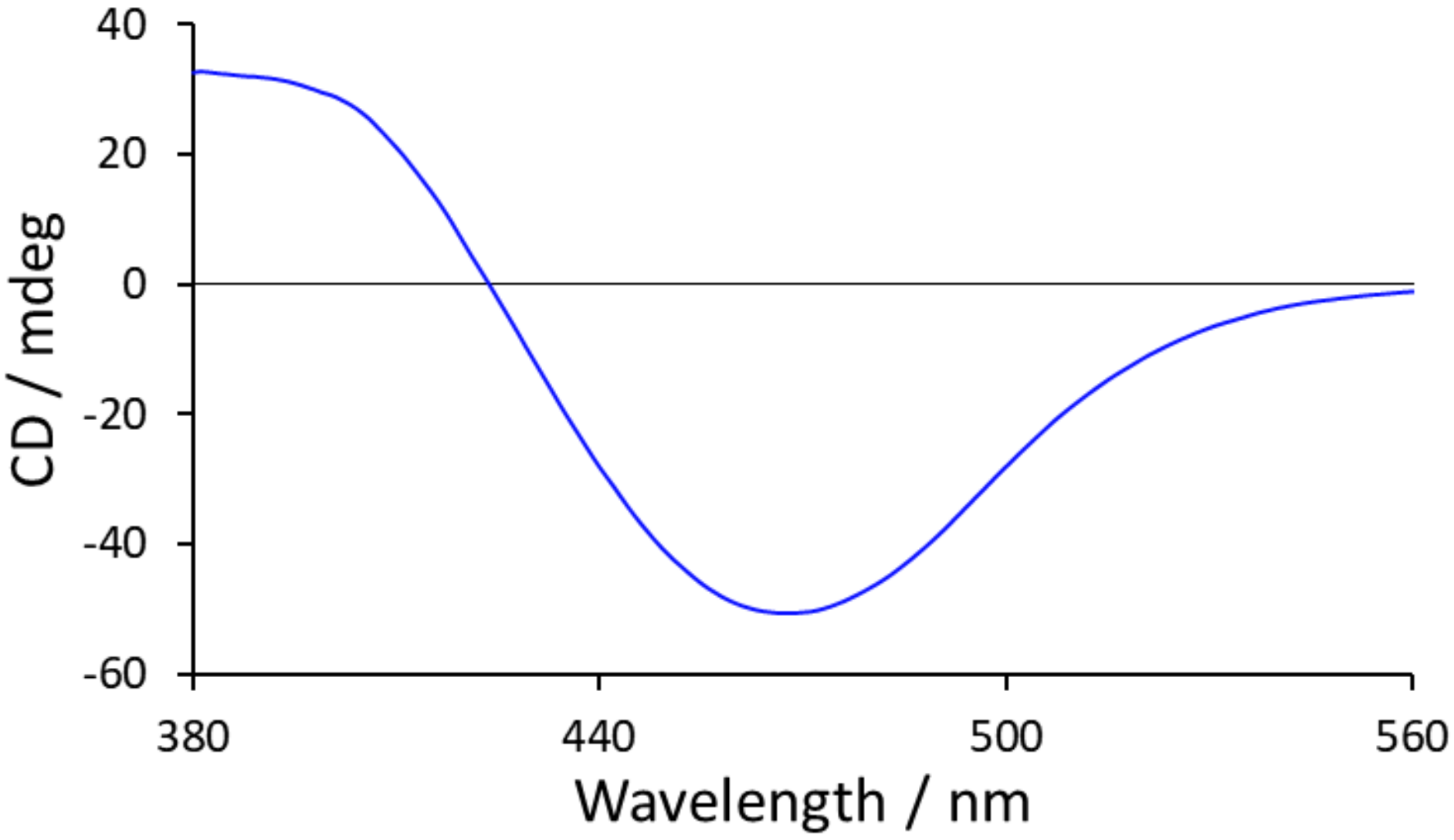

Figure S30: CD spectrum of **1** ($MeNO_2$, 1.5 × 10^{-4} M, 293 K).

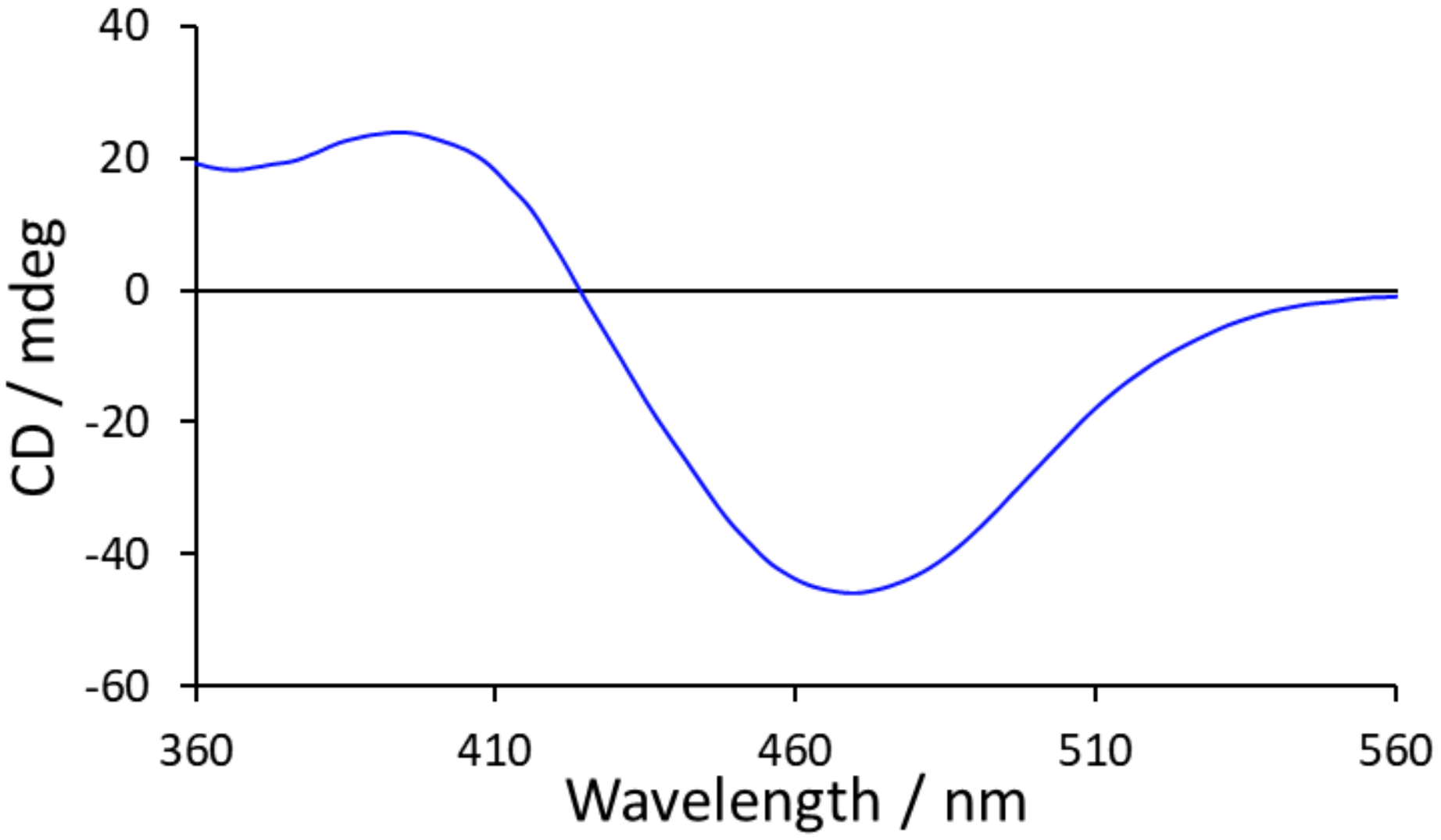

Figure S31: CD spectrum of **1** (DMF, 1.5 × 10^{-4} M, 293 K).

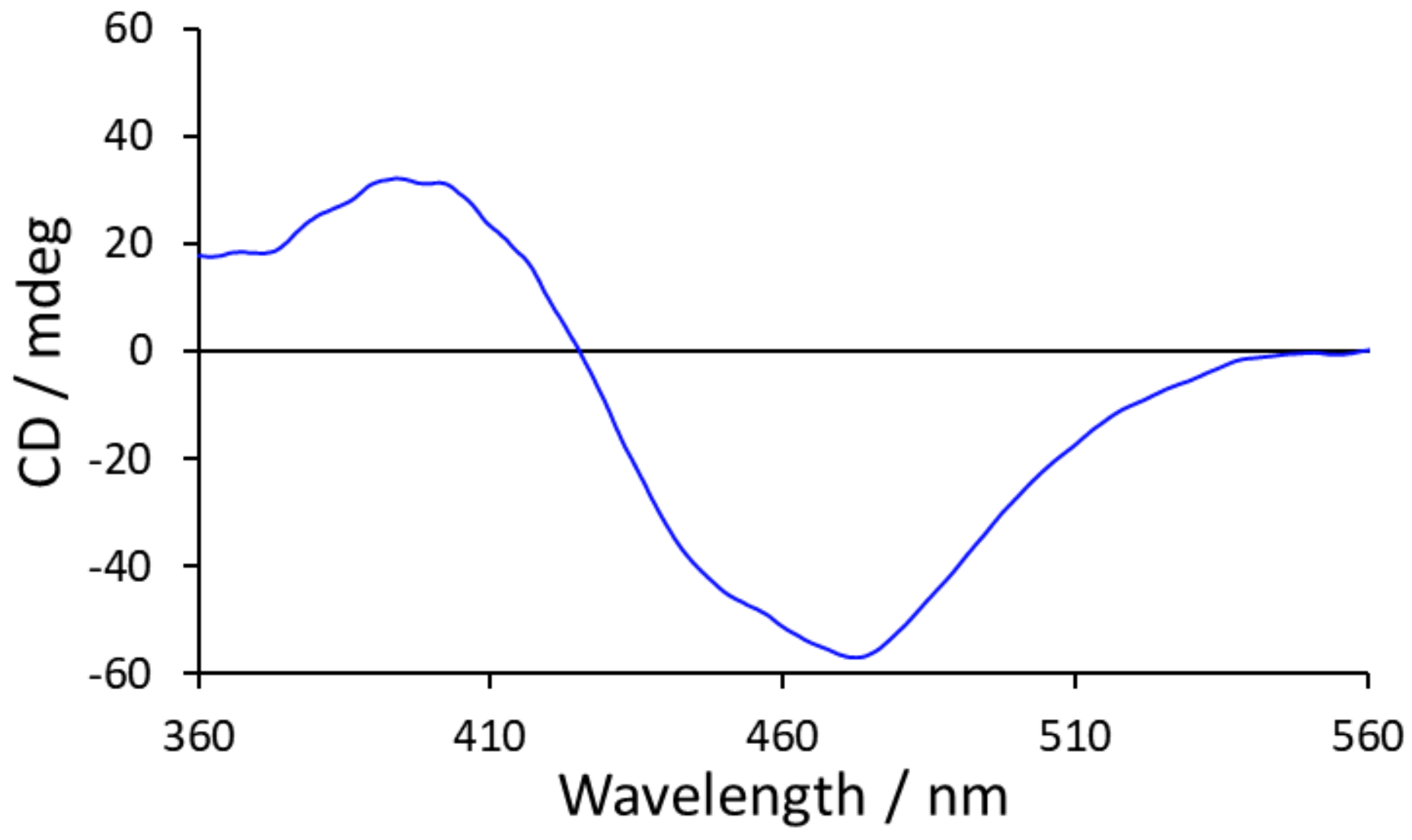

Figure S32: CD spectrum of **1** (MeCN, 1.5 × 10^{-4} M, 293 K).

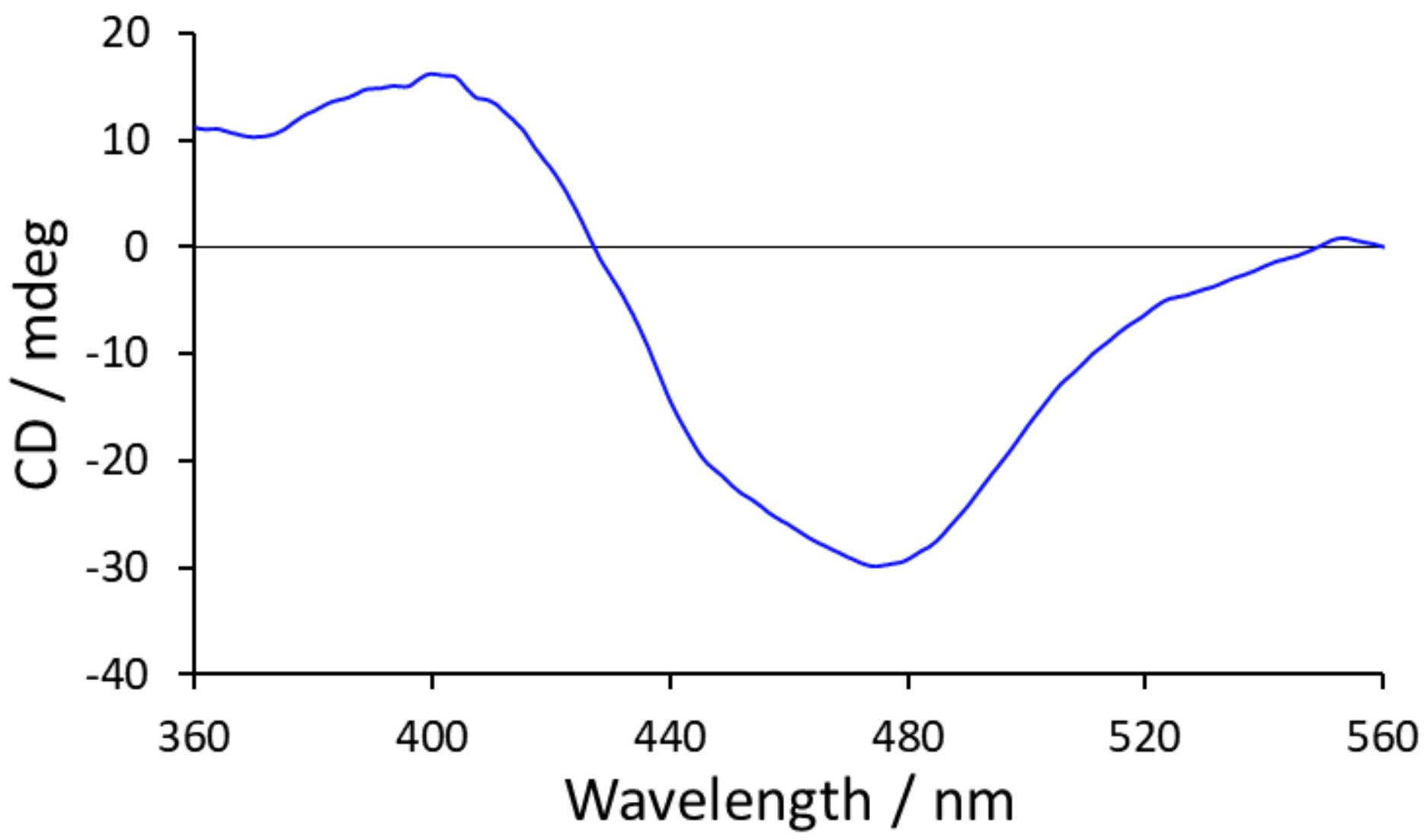

Figure S33: CD spectrum of **1** (DMSO, 1.5 × 10^{-4} M, 293 K).

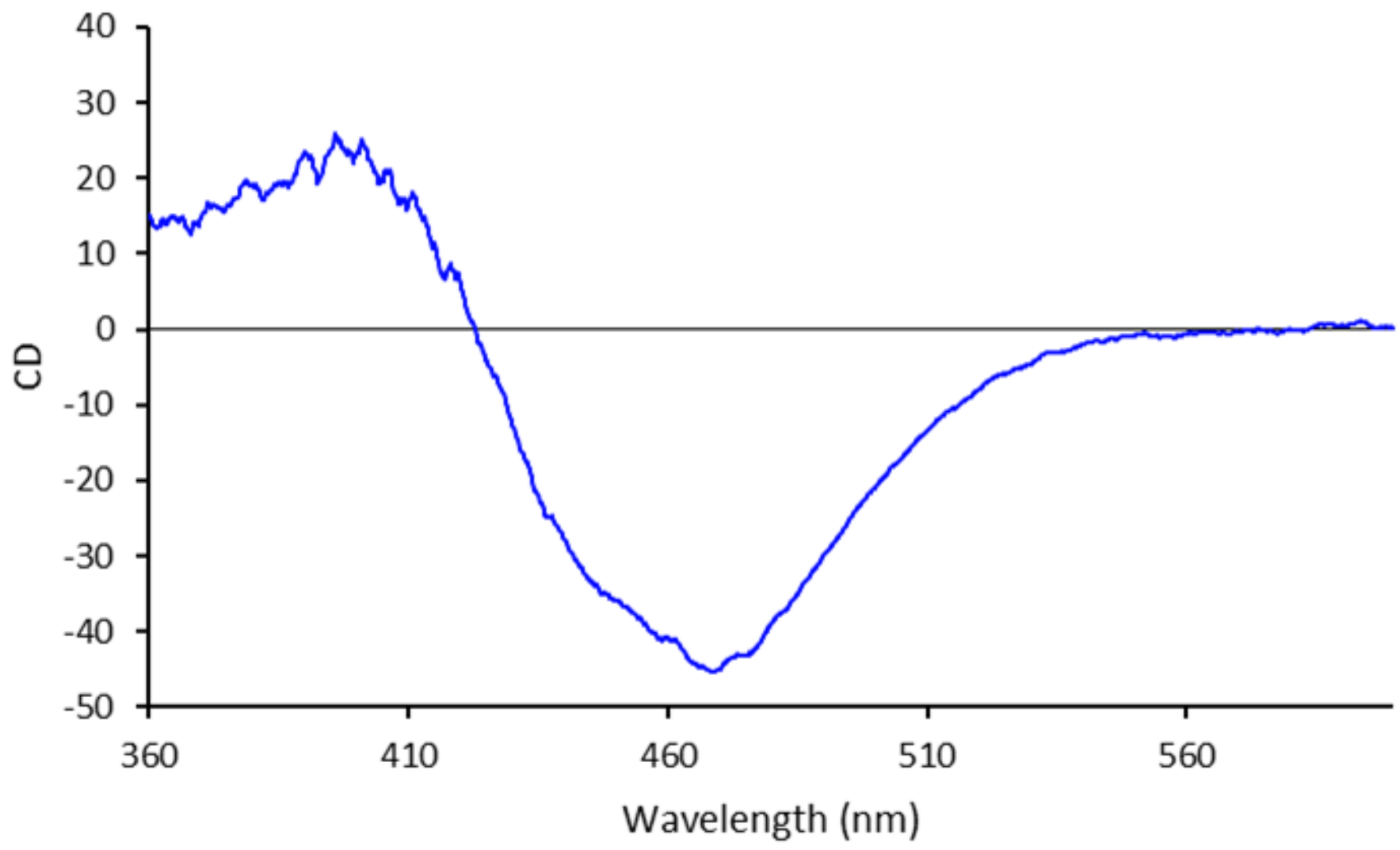

Figure S34: CD spectrum of **1** (MeOH/H_2O, 95/5, 1.5 × 10^{-4} M, 293 K).

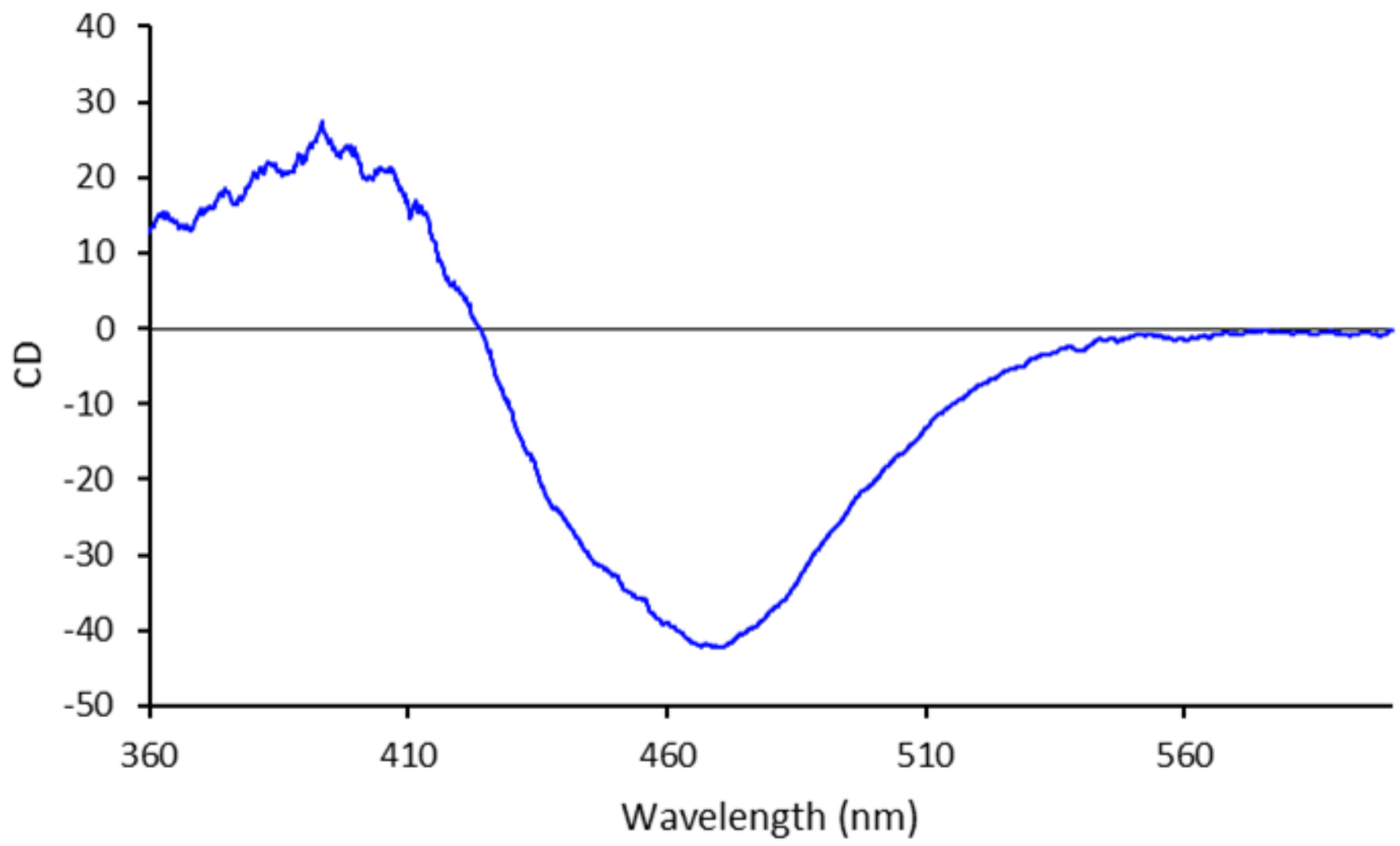

Figure S35: CD spectrum of **1** (MeOH/H_2O, 80/20, 1.5 × 10^{-4} M, 293 K).

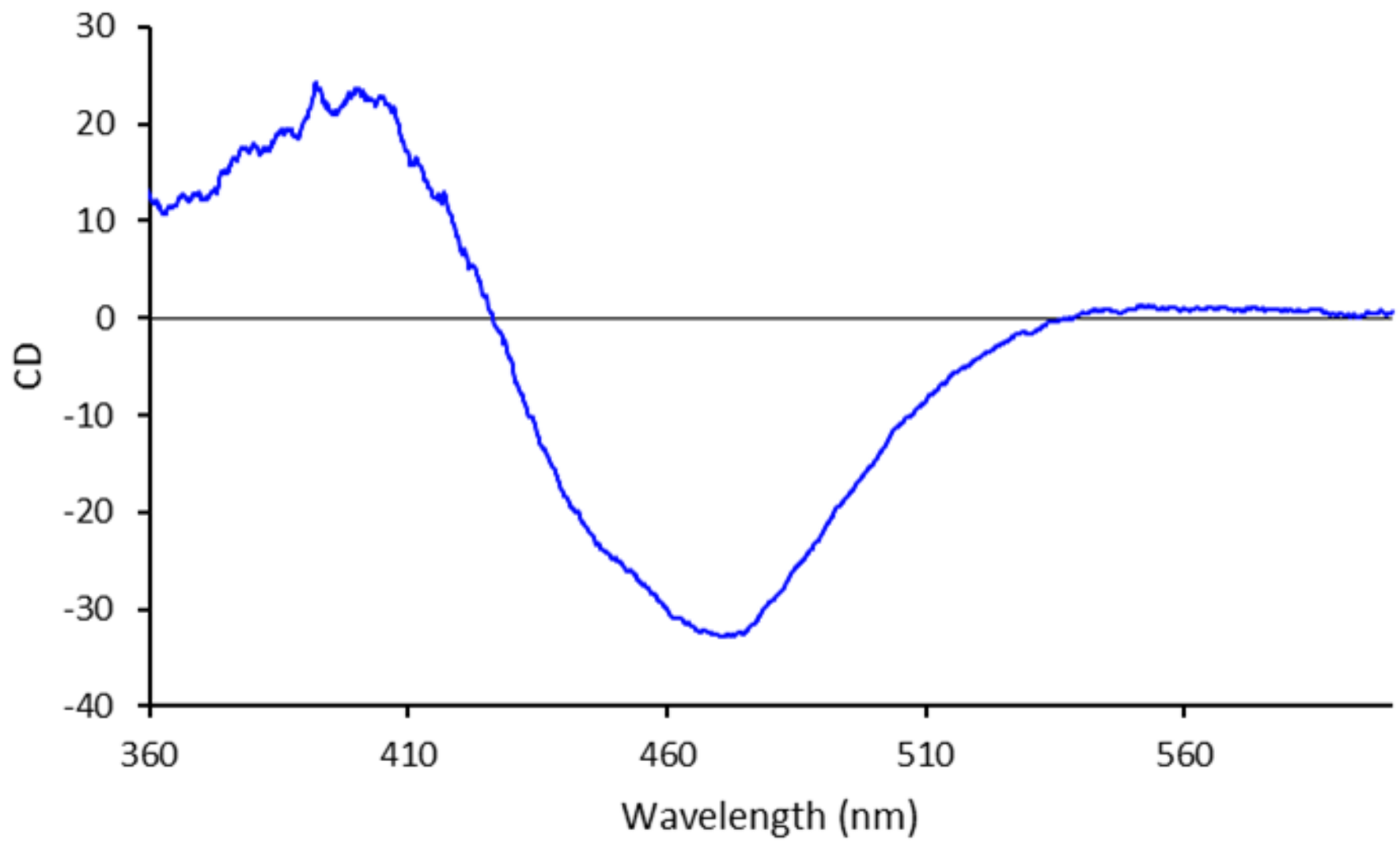

Figure S36: CD spectrum of **1** (MeOH/H_2O, 70/30, 1.5 × 10^{-4} M, 293 K).

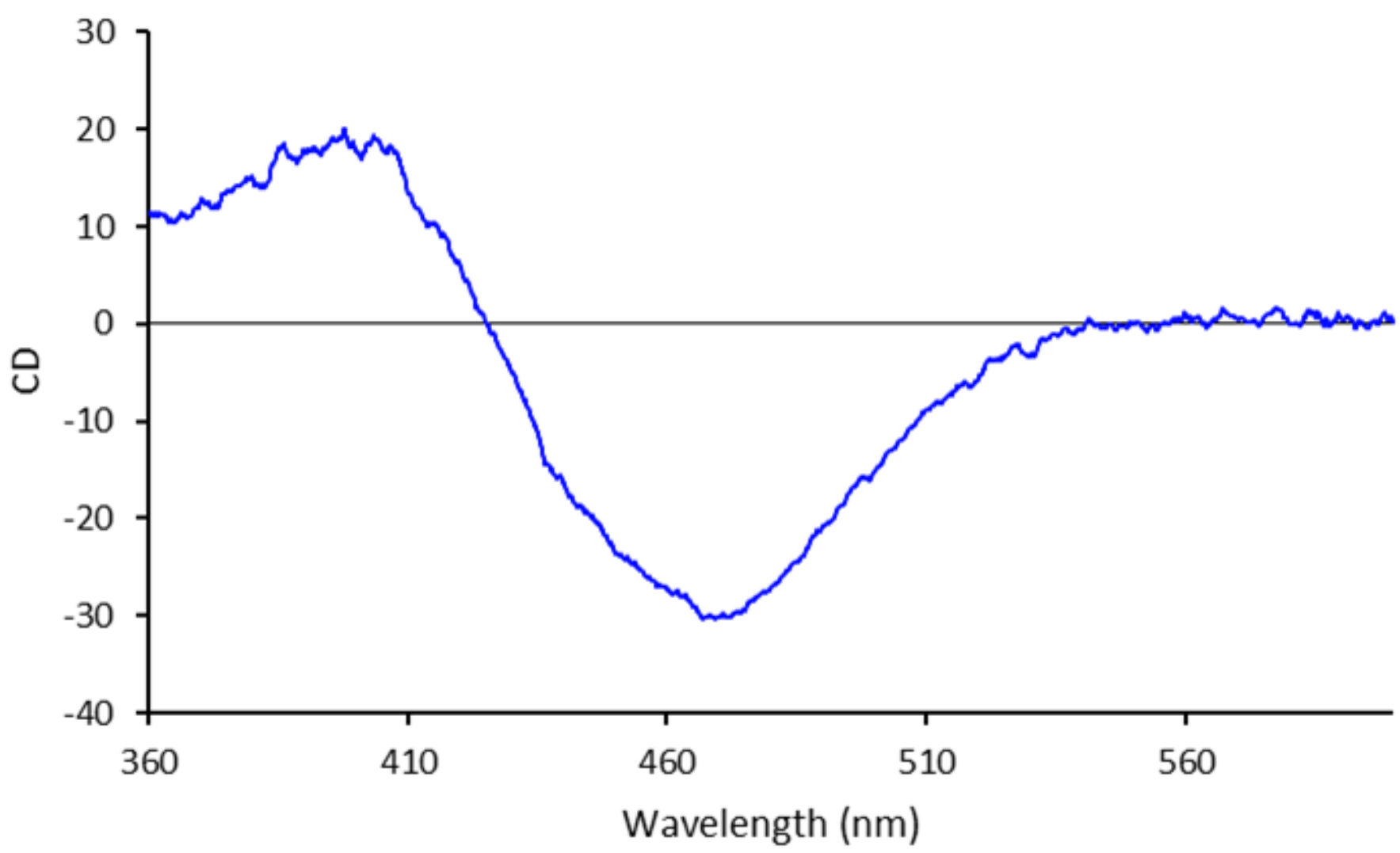

Figure S37: CD spectrum of **1** (MeOH/H_2O, 65/35, 1.5 × 10^{-4} M, 293 K).

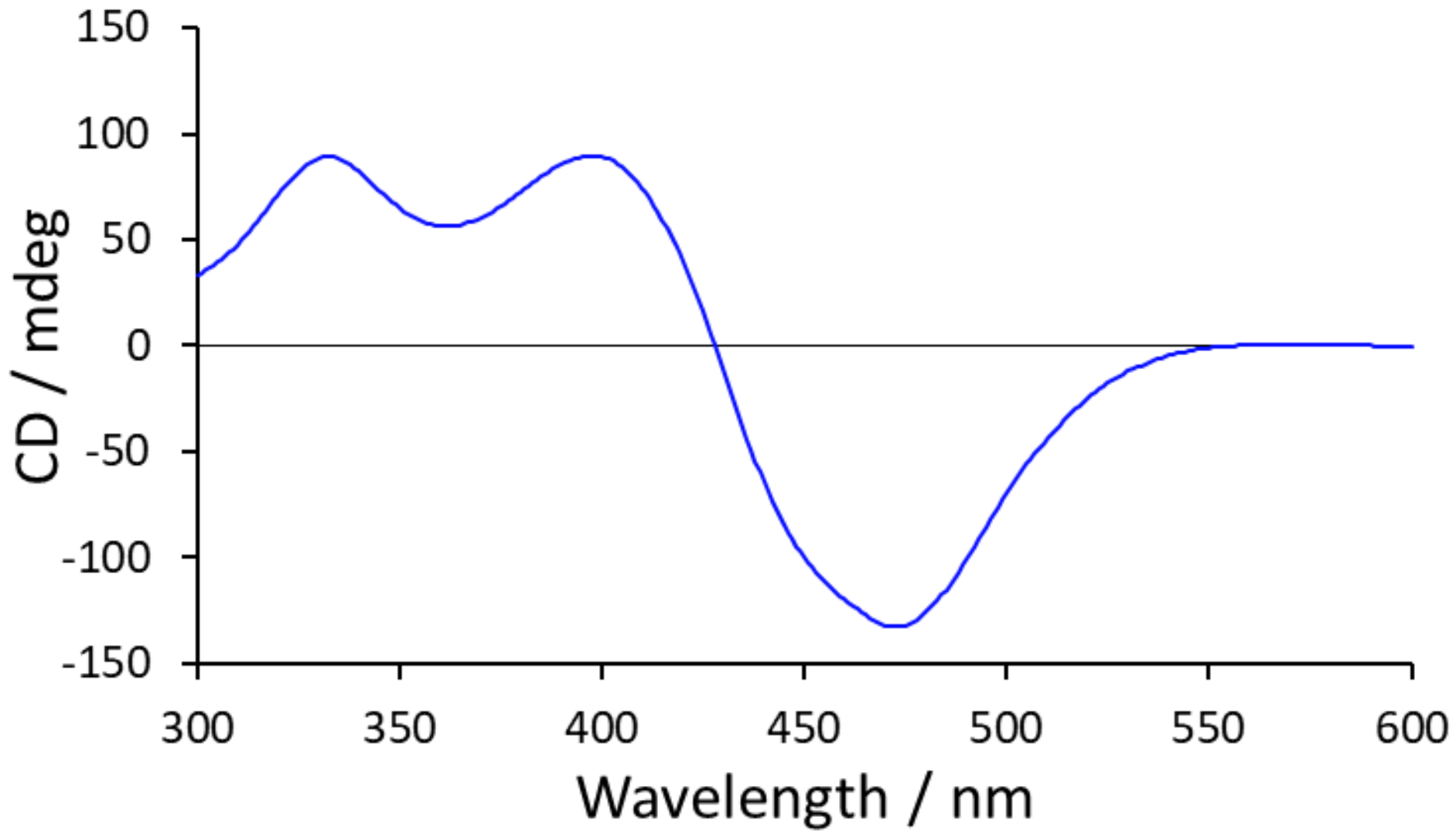

Figure S38: CD spectrum of **1** (Benzene, 1.5 × 10^{-4} M, 293 K).

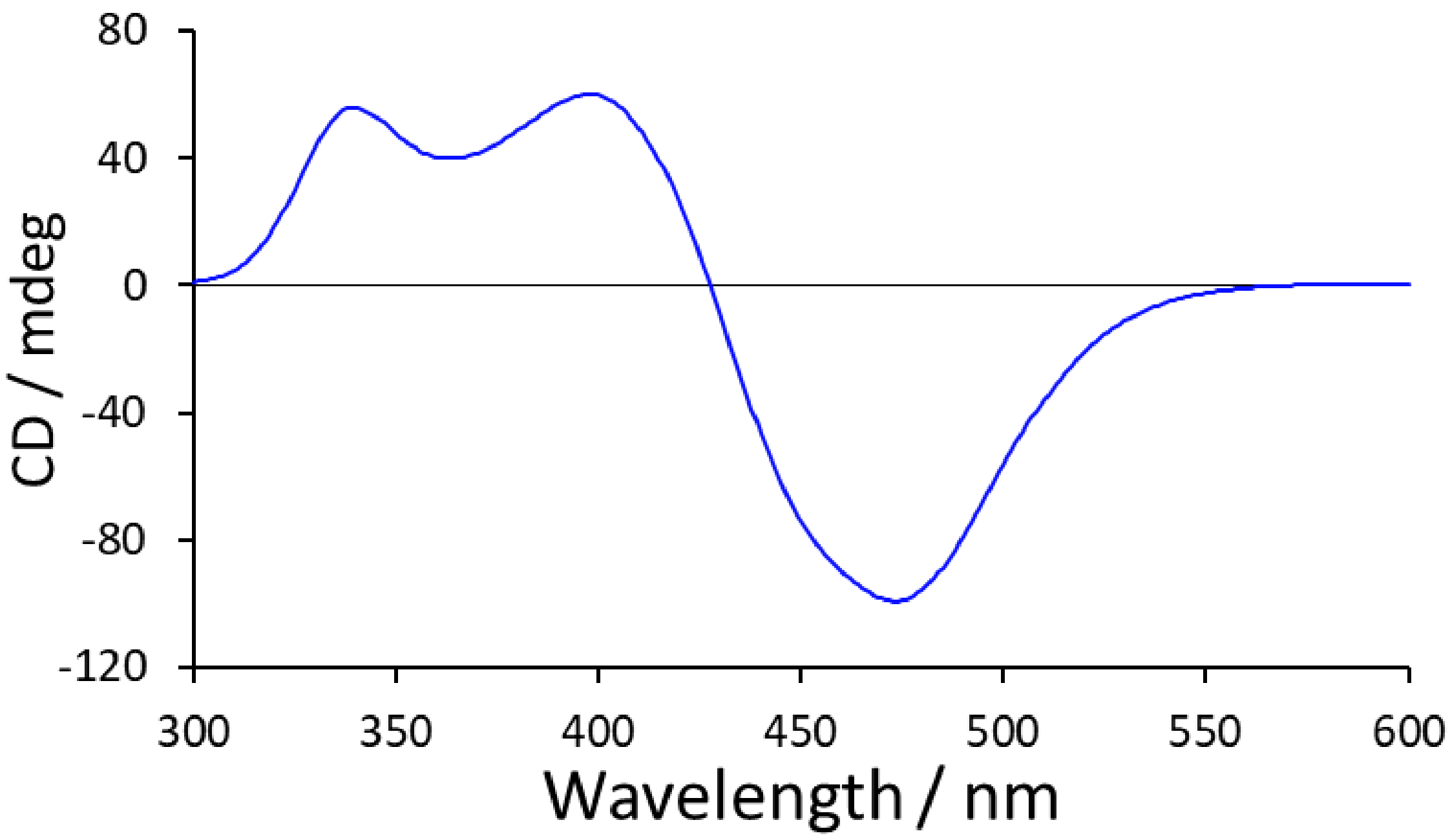

Figure S39: CD spectrum of **1** (pyridine, 1.5 × 10^{-4} M, 293 K).

UV/Vis Spectroscopic Studies

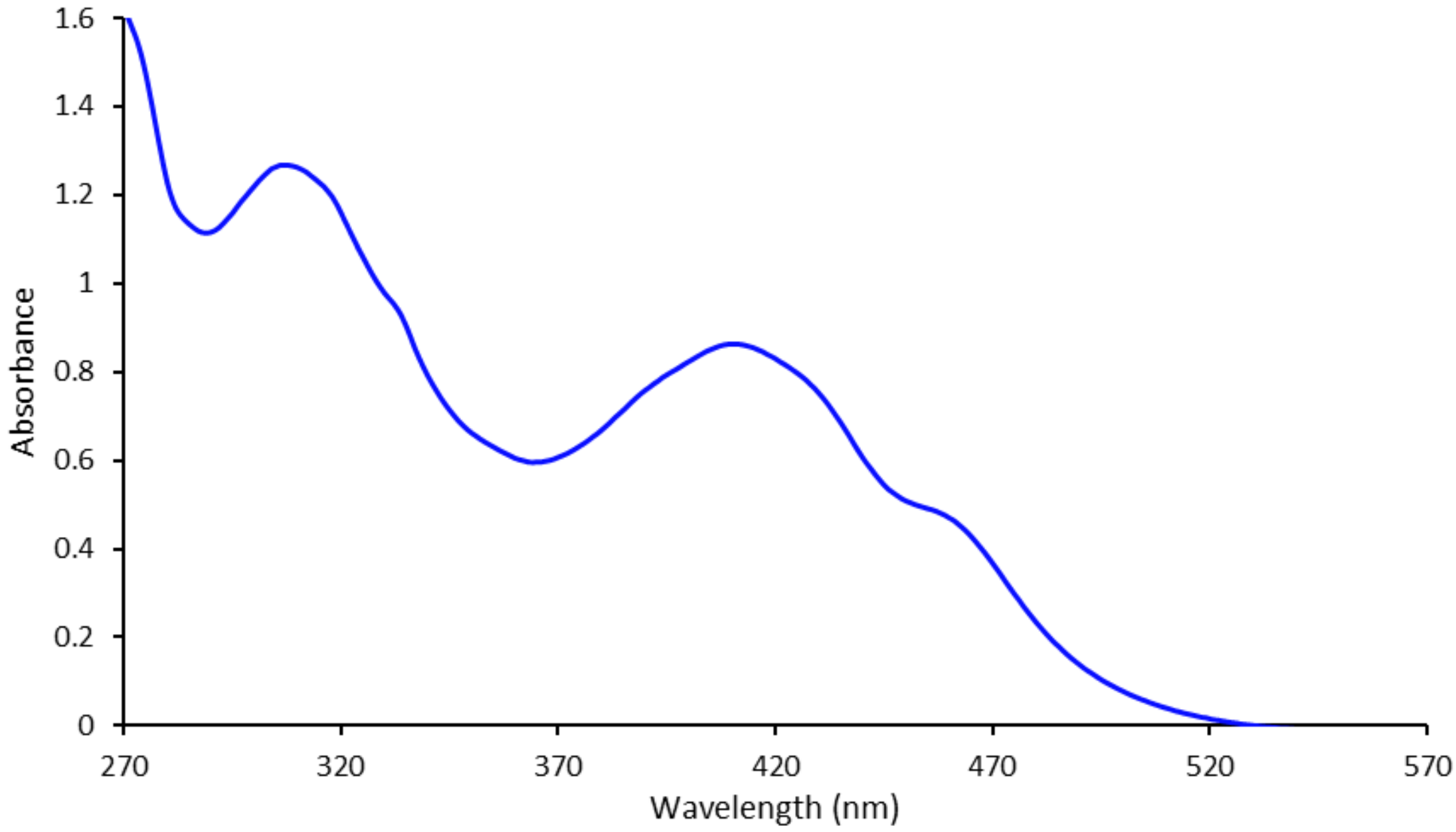

Figure S40: UV/Vis spectrum of **1** (DCM, 4.4 × 10^{-5} M, 298 K).

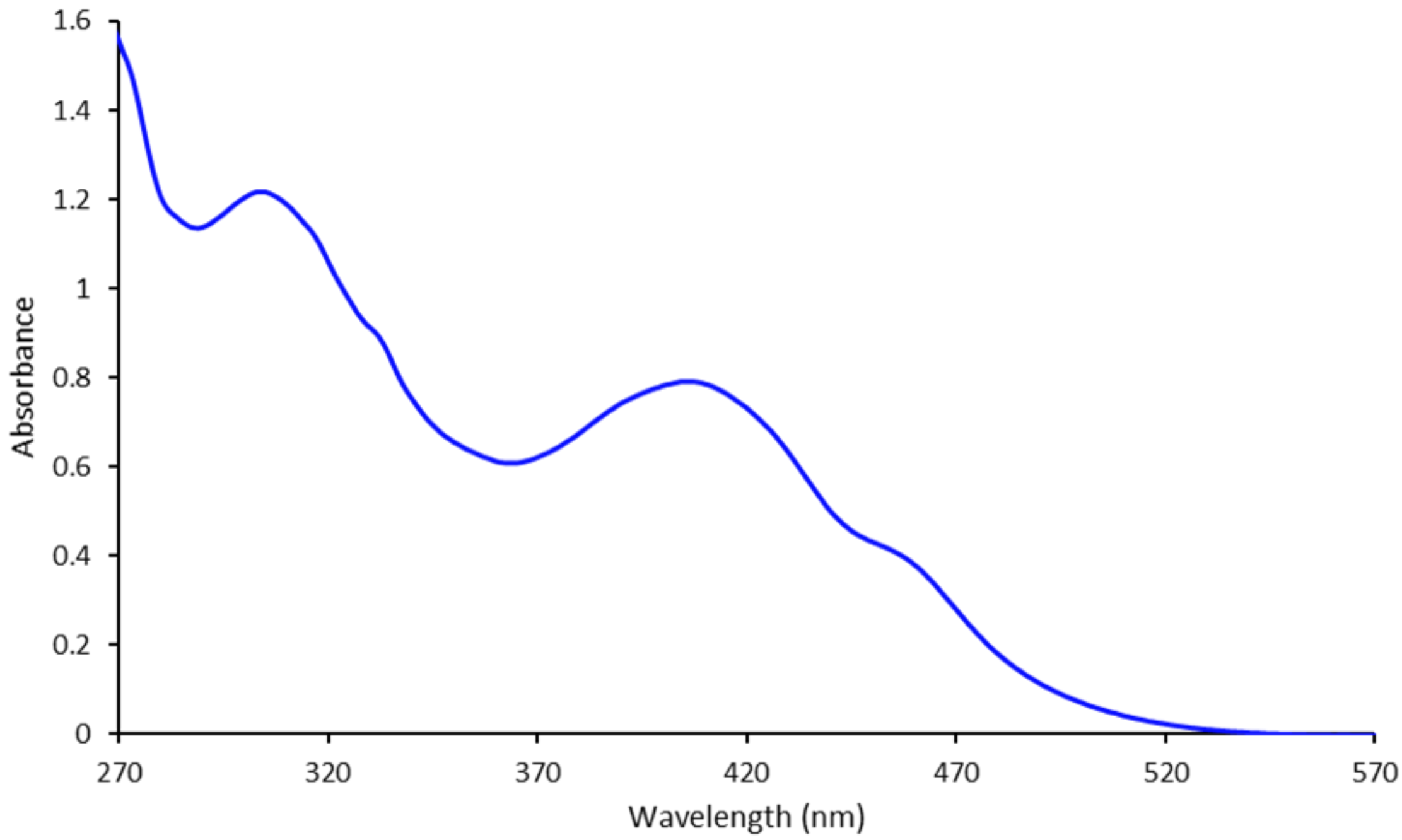

Figure S41: UV/Vis spectrum of **1** (MeOH, 4.4 × 10^{-5} M, 298 K).

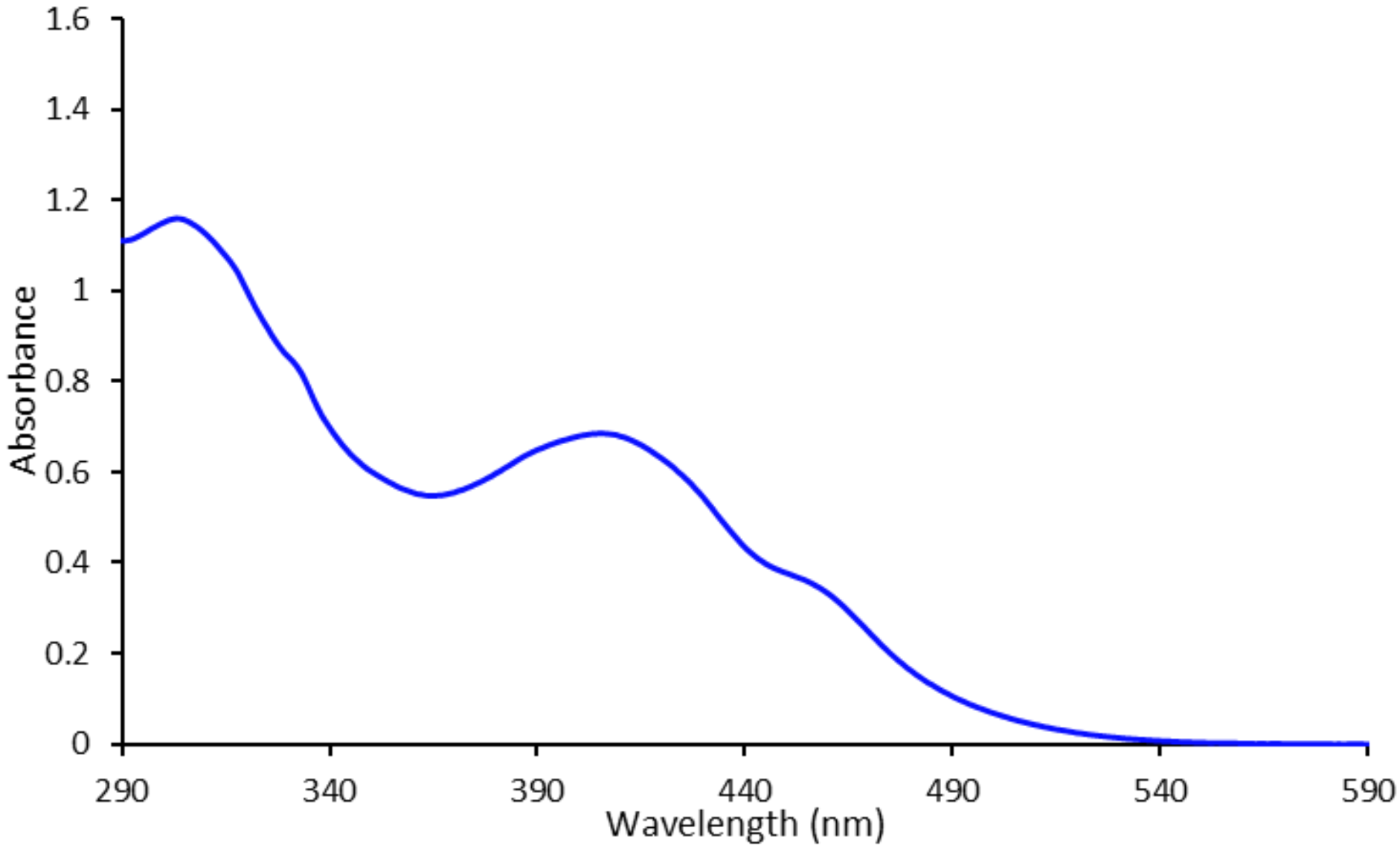

Figure S42: UV/Vis spectrum of **1** (MeOH/H_2O, 95/5, 4.4 × 10^{-5} M, 298 K).

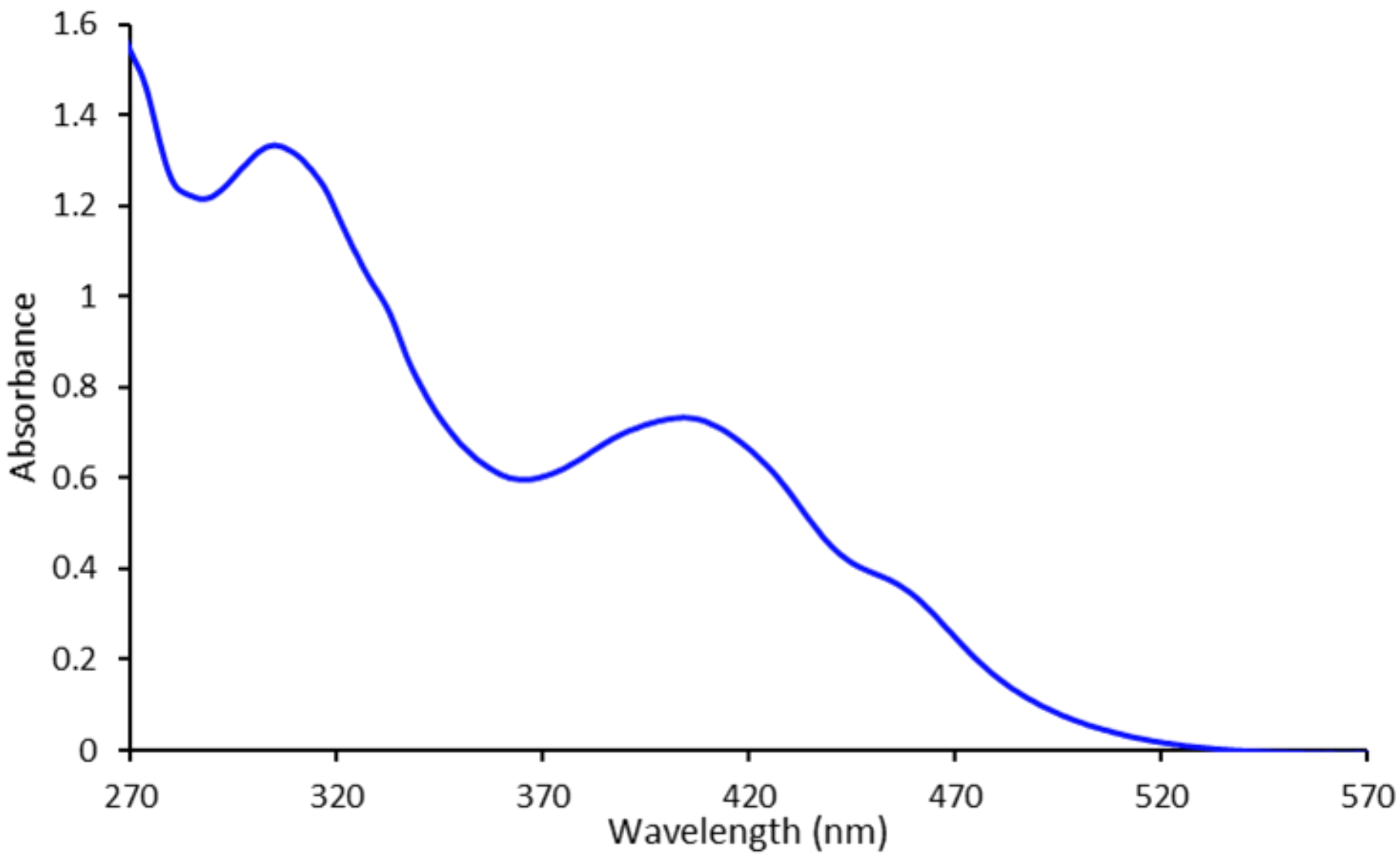

Figure S43: UV/Vis spectrum of **1** (MeOH/H_2O, 80/20, 4.4 × 10^{-5} M, 298 K).

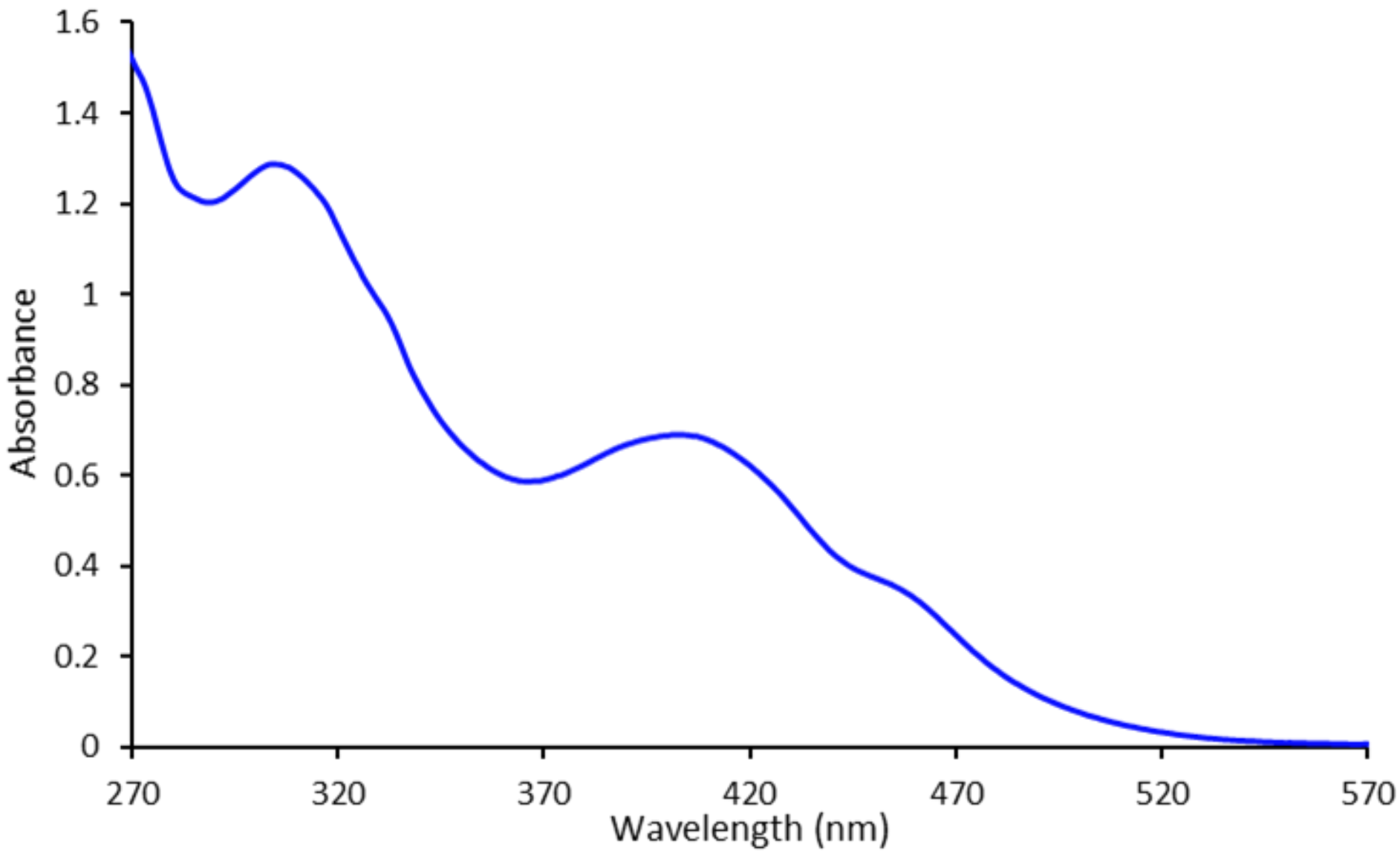

Figure S44: UV/Vis spectrum of **1** (MeOH/H_2O, 70/30, 4.4 × 10^{-5} M, 298 K).

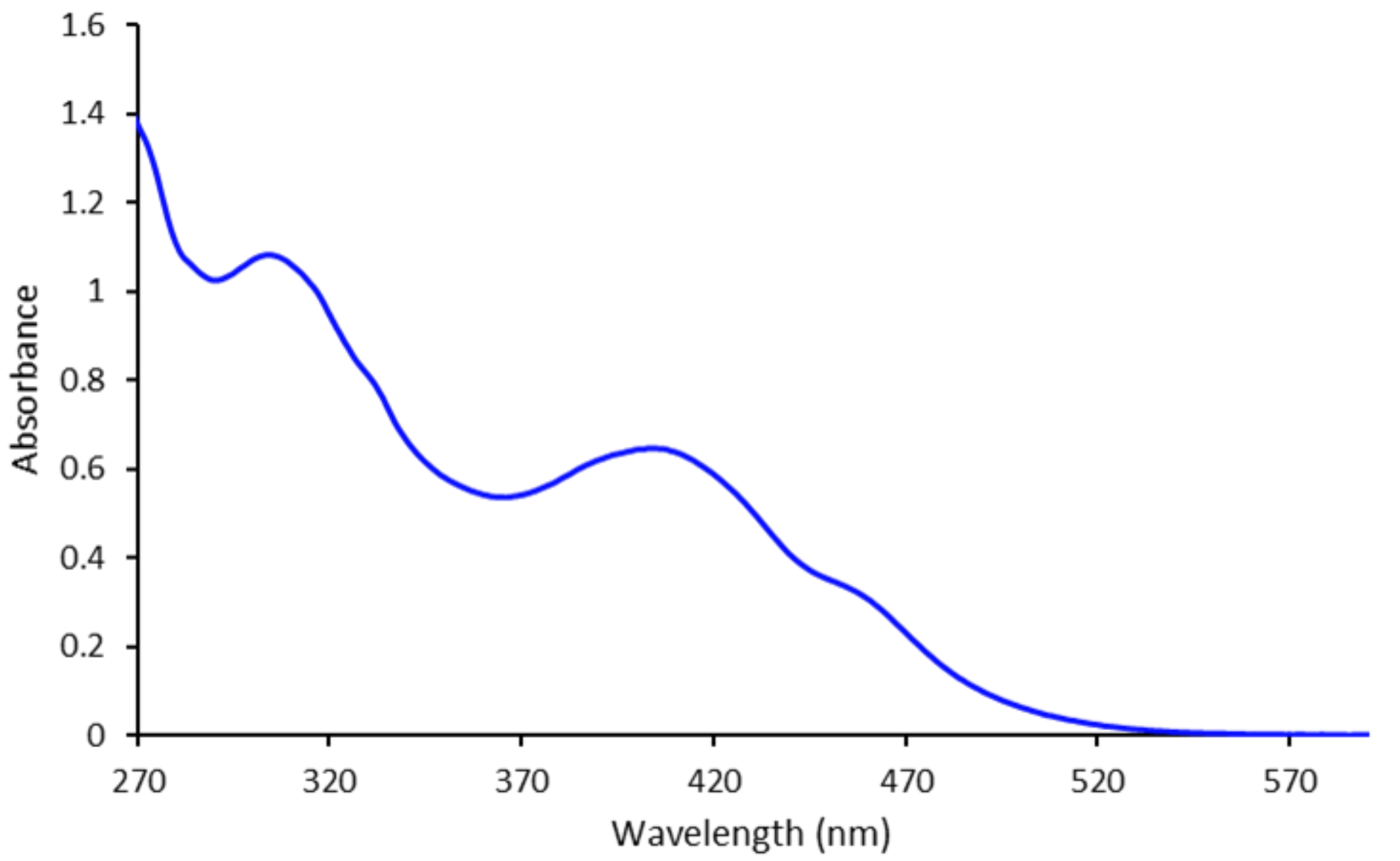

Figure S45: UV/Vis spectrum of **1** (MeOH/H_2O, 65/35, 4.4 × 10^{-5} M, 298 K).

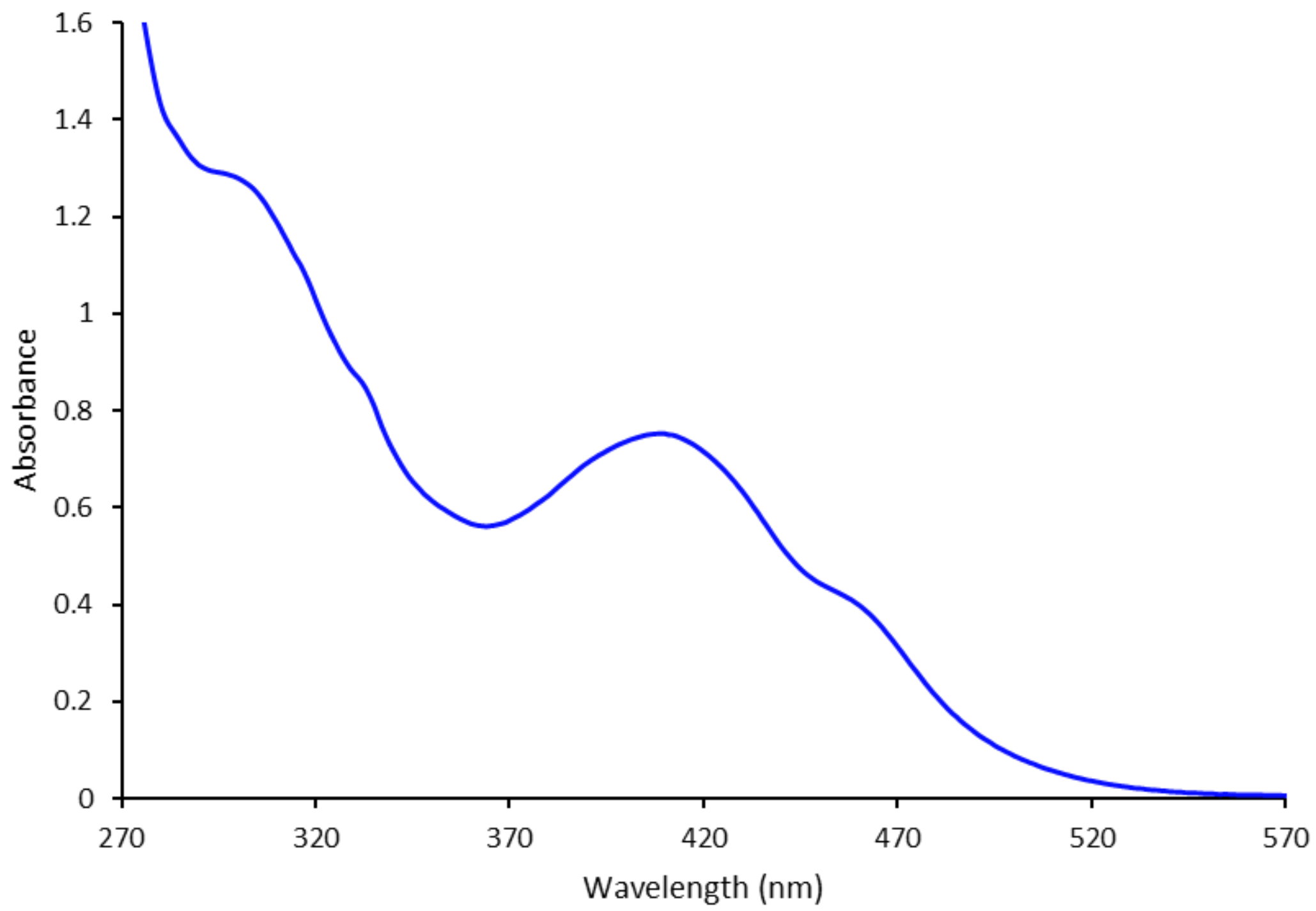

Figure S46: UV/Vis spectrum of **1** (MeCN, 4.4 × 10^{-5} M, 298 K).

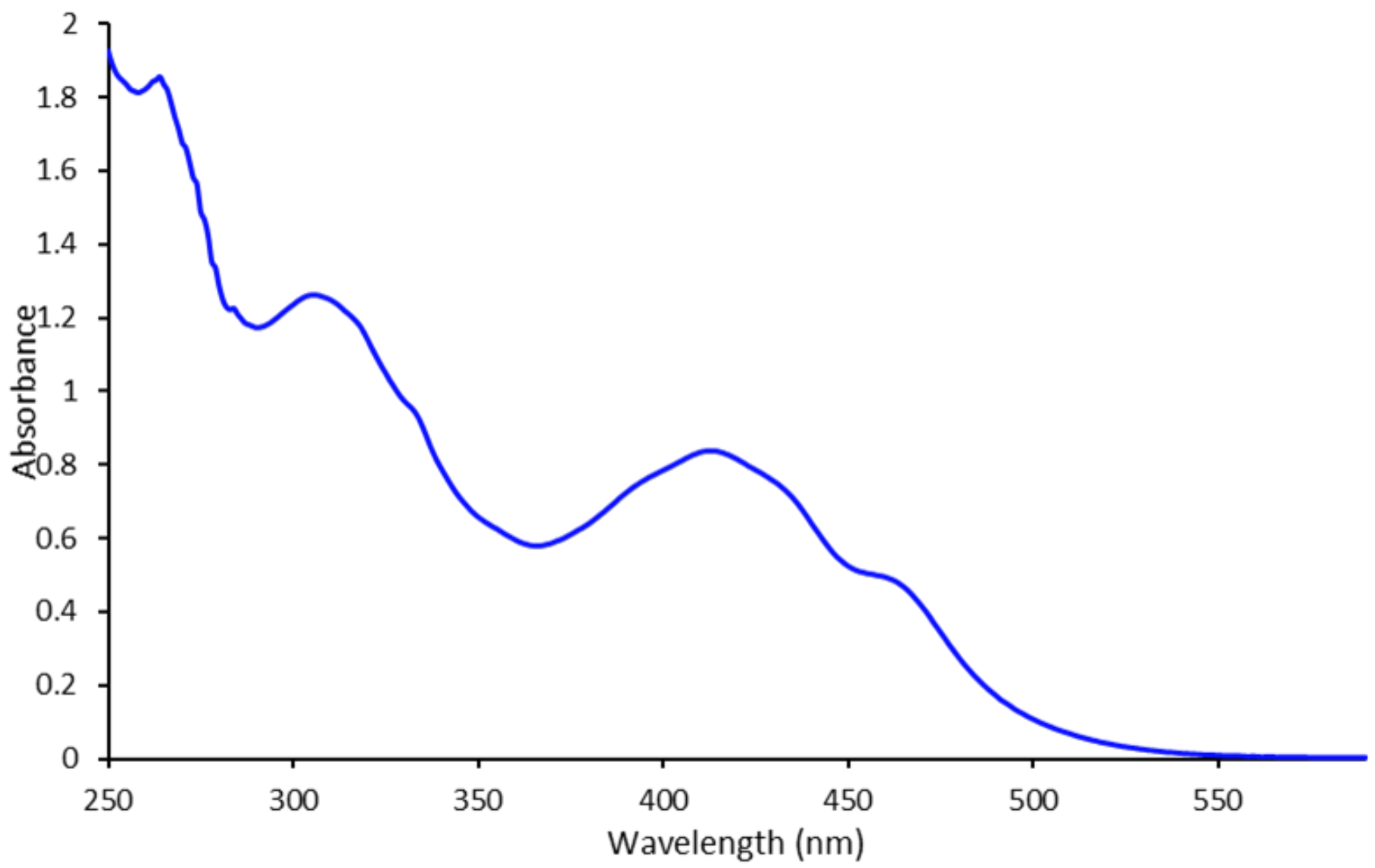

Figure S47: UV/Vis spectrum of **1** (THF, 4.4 × 10^{-5} M, 298 K).

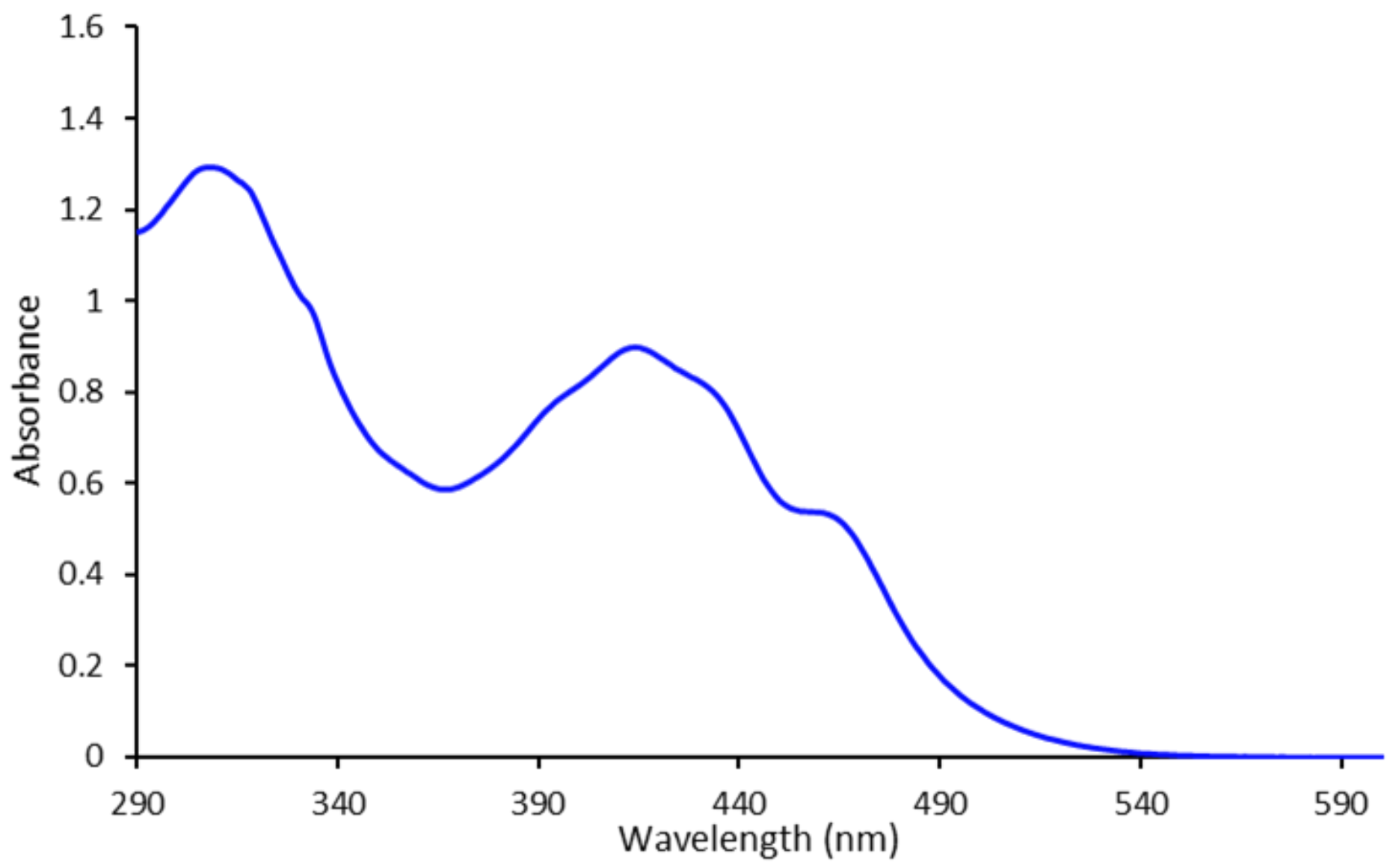

Figure S48: UV/Vis spectrum of **1** (Toluene, 4.4 × 10^{-5} M, 298 K).

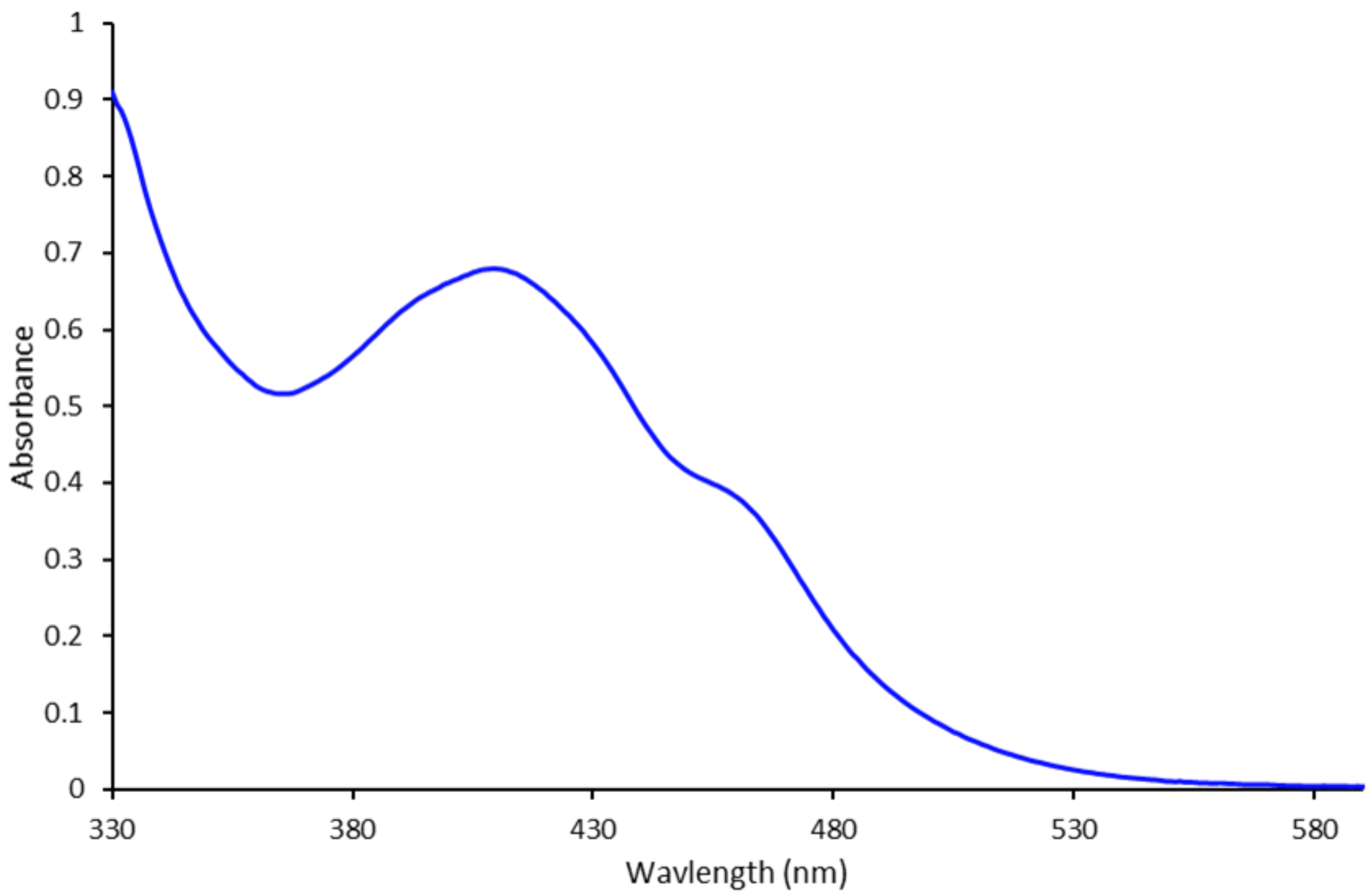

Figure S49: UV/Vis spectrum of **1** (Acetone, 4.4 × 10^{-5} M, 298 K).

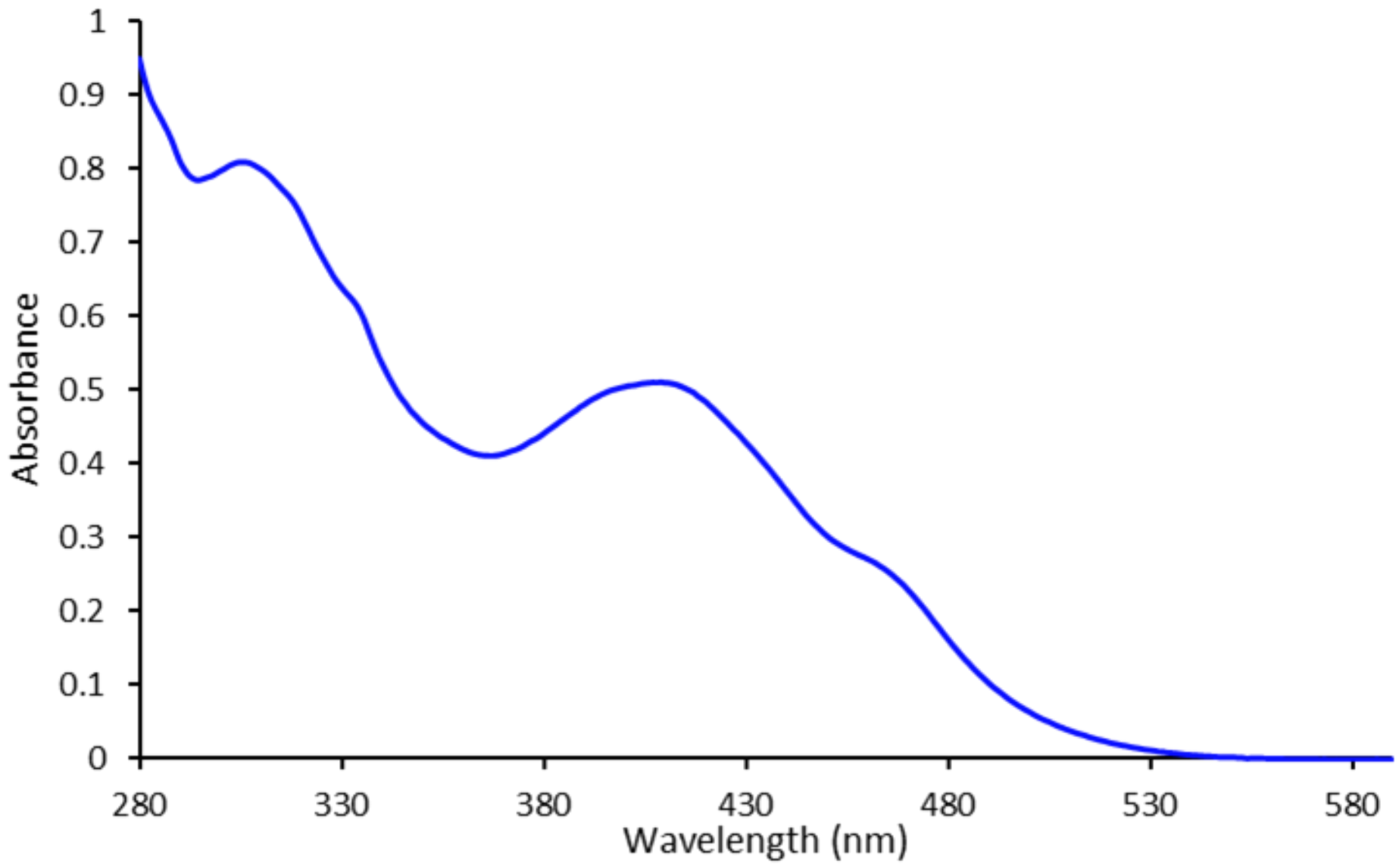

Figure S50: UV/Vis spectrum of **1** (DMSO, 4.4 × 10^{-5} M, 298 K).

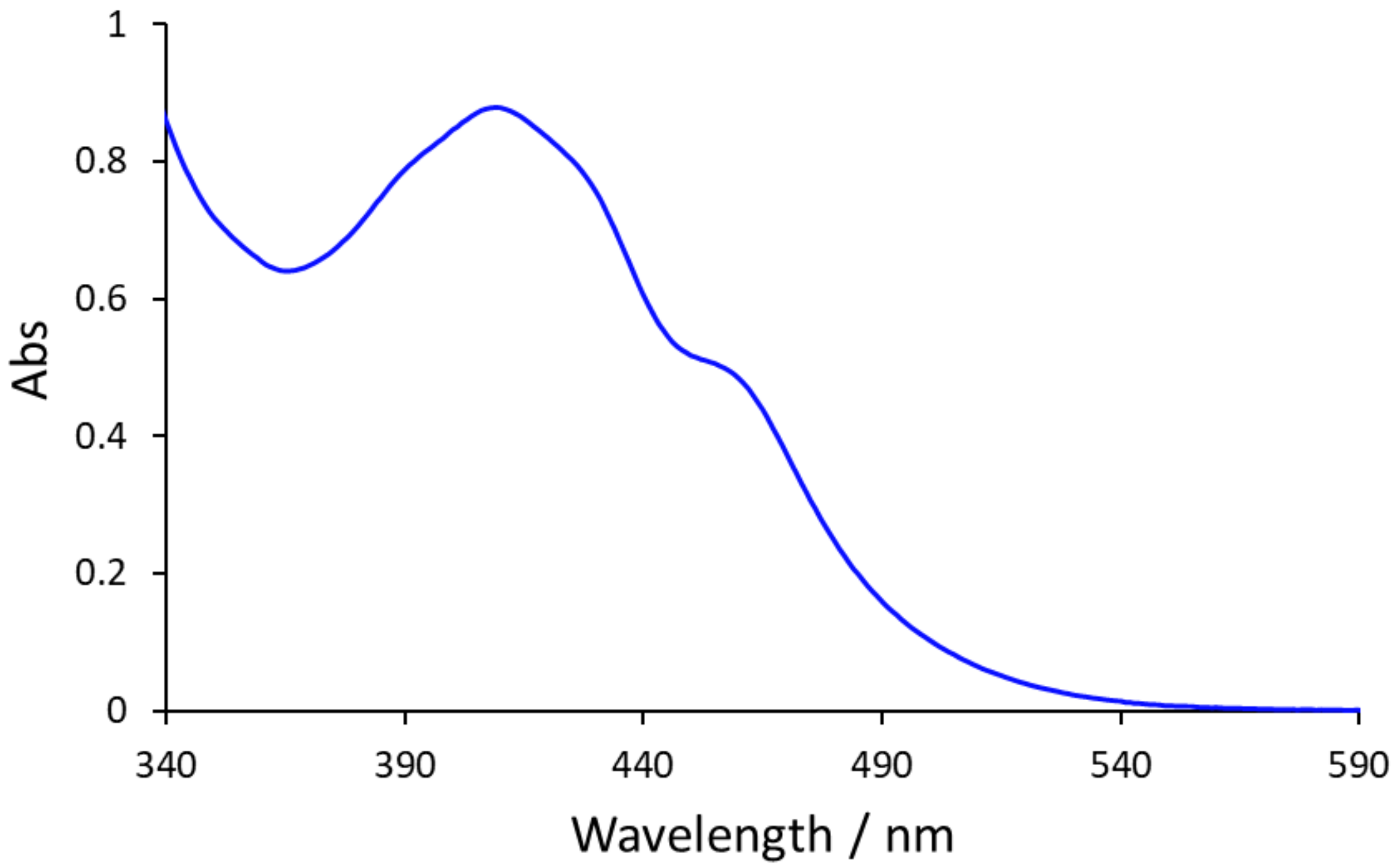

Figure S51: UV/Vis spectrum of **1** (Chloroform, 4.4 × 10^{-5} M, 298 K).

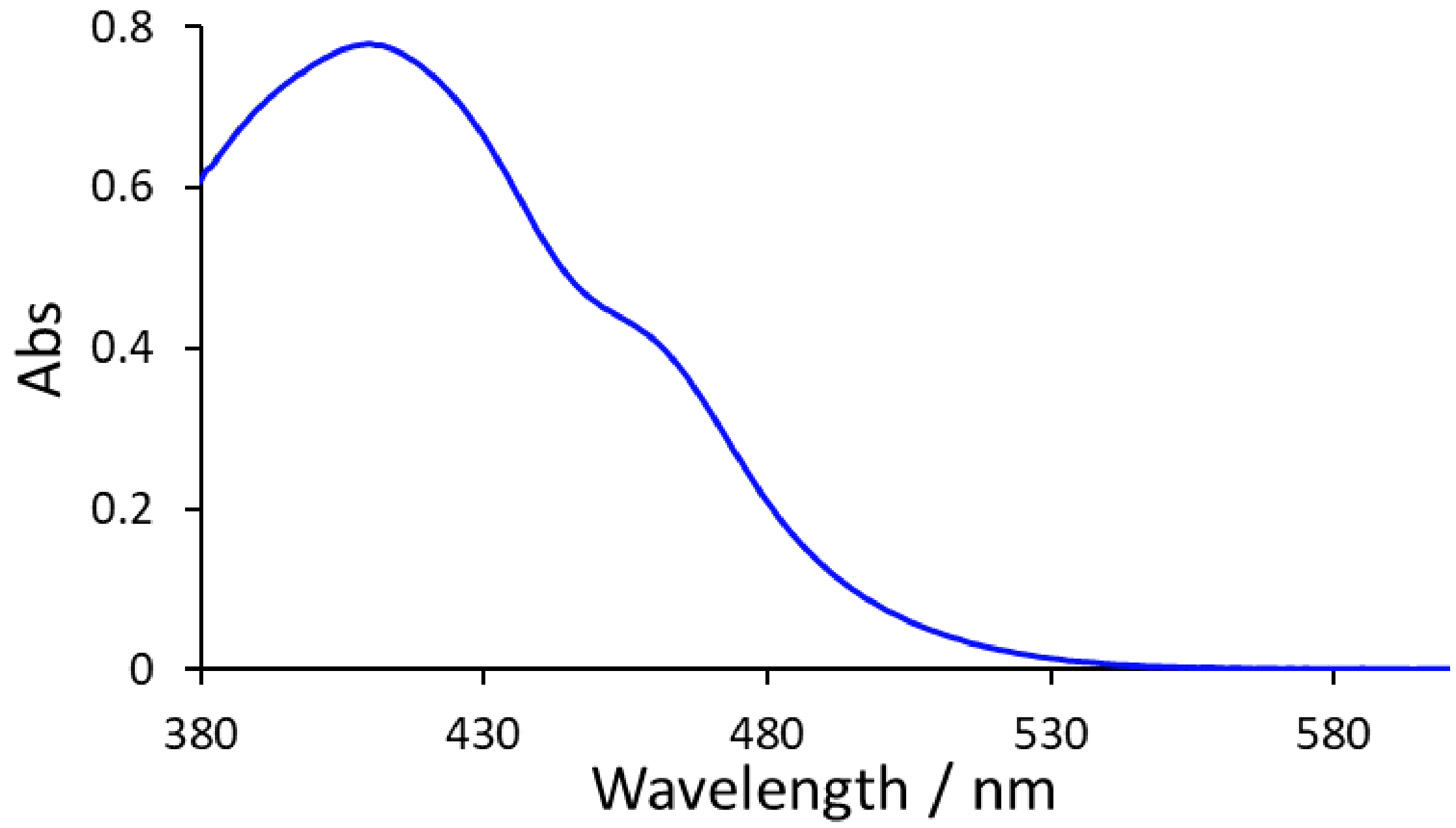

Figure S52: UV/Vis spectrum of **1** (Nitromethane, 4.4×10^{-5} M, 298 K).

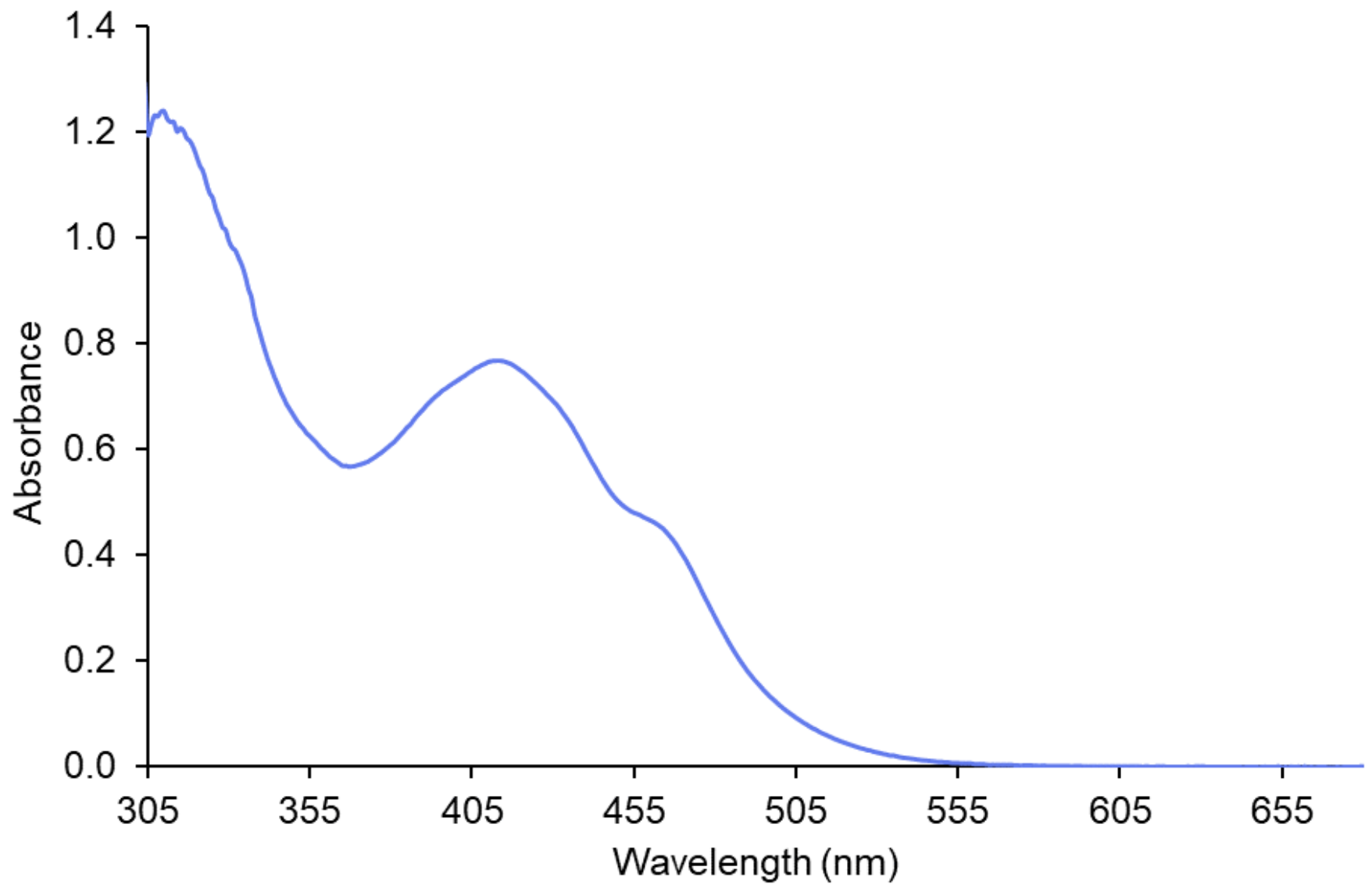

Figure S53: UV/Vis spectrum of **1** (pyridine, 4.4 × 10^{-5} M, 298 K).

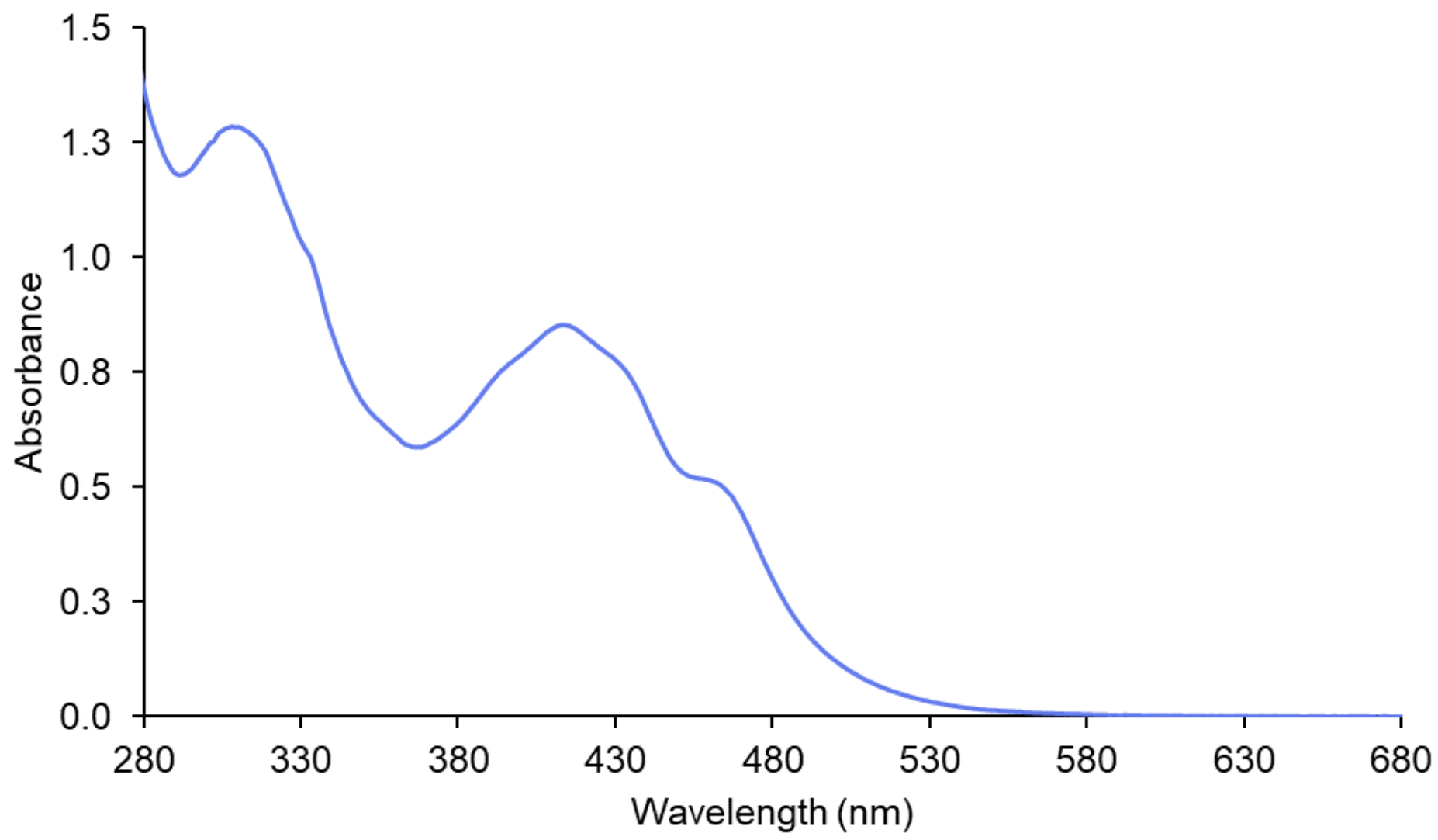

Figure S54: UV/Vis spectrum of **1** (benzene, 4.4 × 10^{-5} M, 298 K).

Solvent Polarity

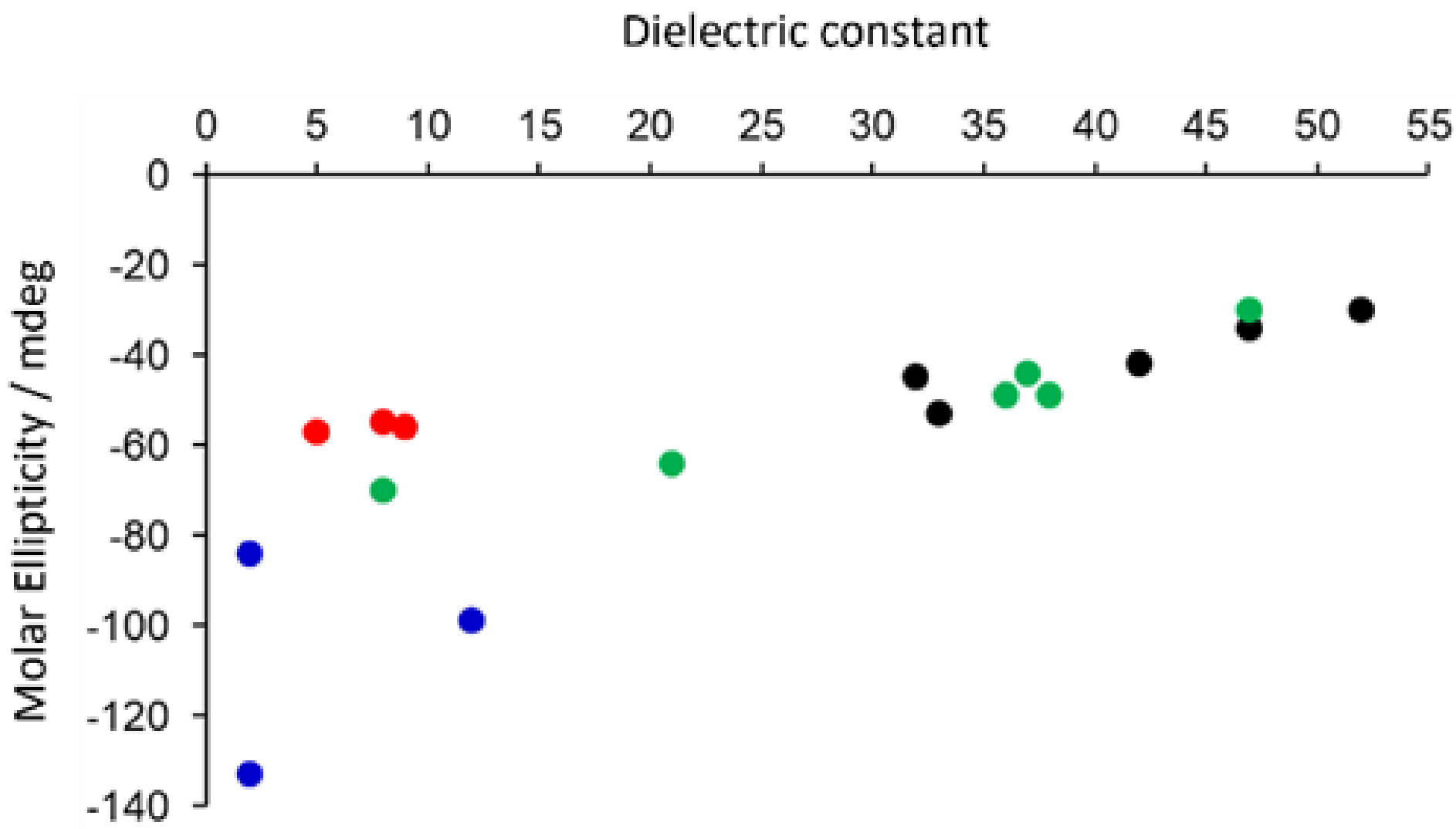

Figure S55: Graph showing a plot of molar ellipticity of the minima centred between 466-475 nm of **1** as observed by CD spectroscopy in the studied solvents against their dielectric constants. Aromatic solvents shown in blue, chlorinated solvents shown in red, polar aprotic solvents shown in green and polar protic solvents shown in black.

^{1}H NMR Spectroscopy Dilution studies

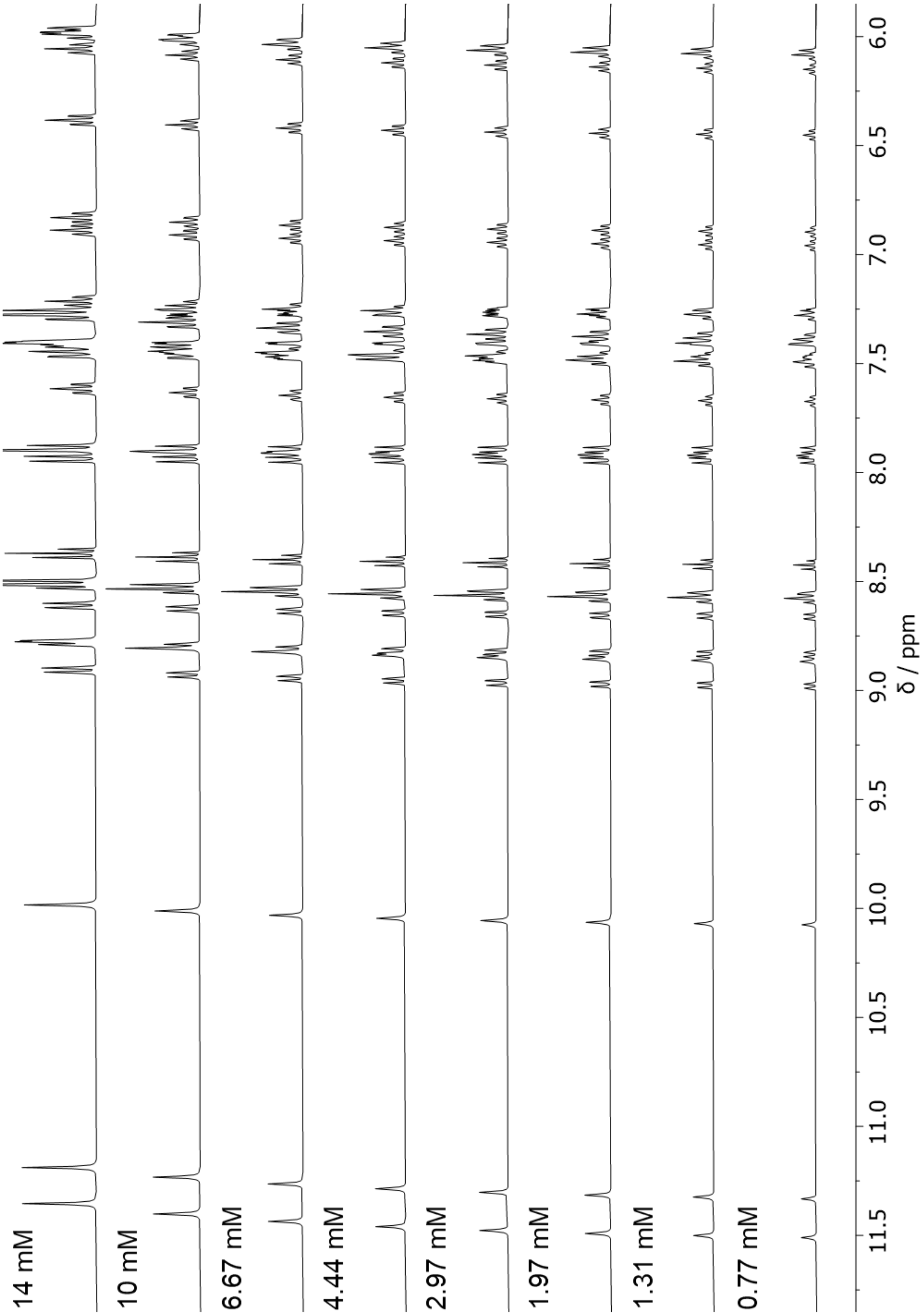

Figure S56: Dilution study showing partial ^{1}H NMR spectra of **1** (400 MHz, Acetone-d_6, 298 K).

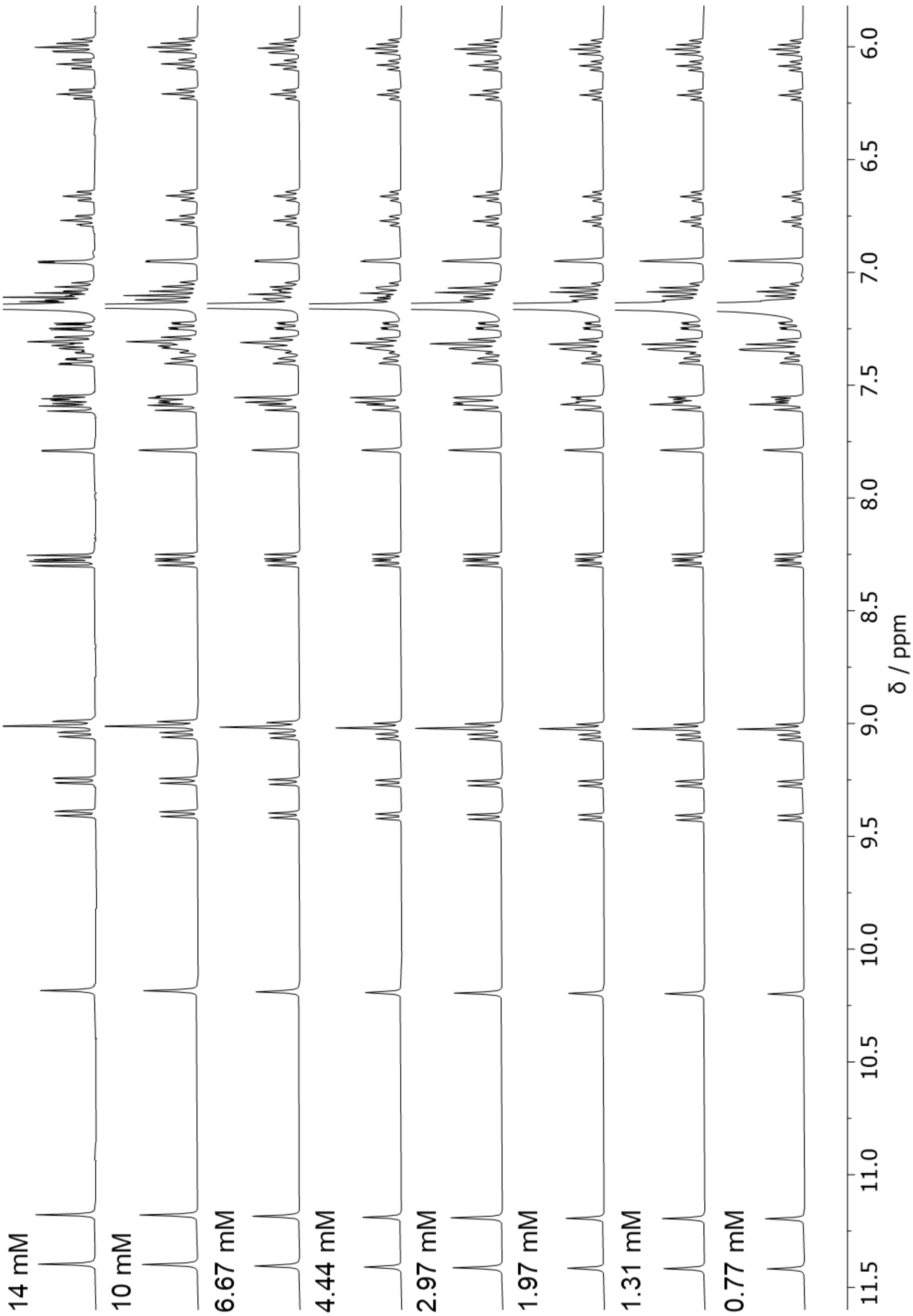

Figure S57: Dilution study showing partial ^{1}H NMR spectra of **1** (400 MHz, Benzene-d_6, 298 K).

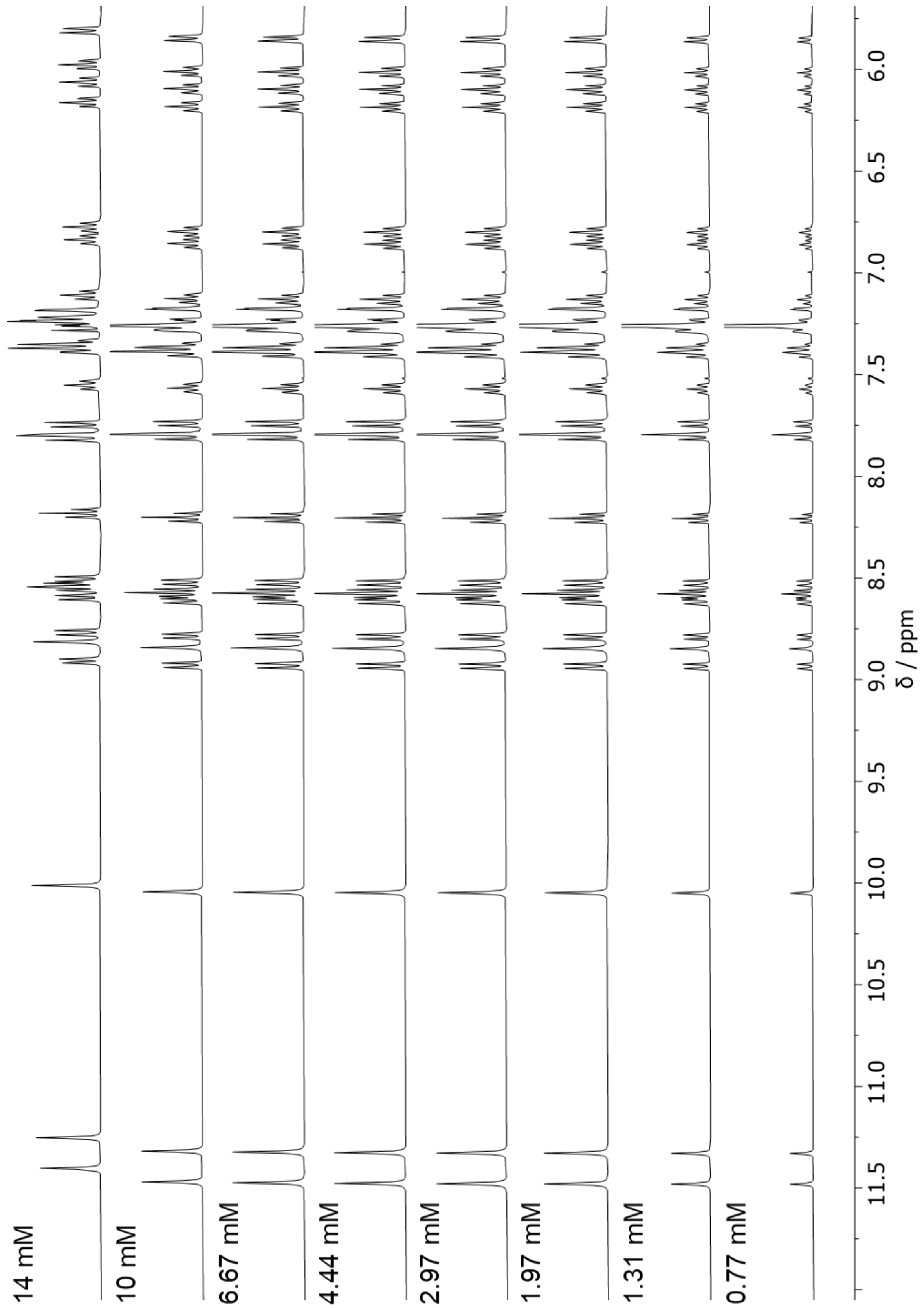

Figure S58. Dilution study showing partial ^{1}H NMR spectra of **1** (400 MHz, Chloroform-d_1, 298 K).

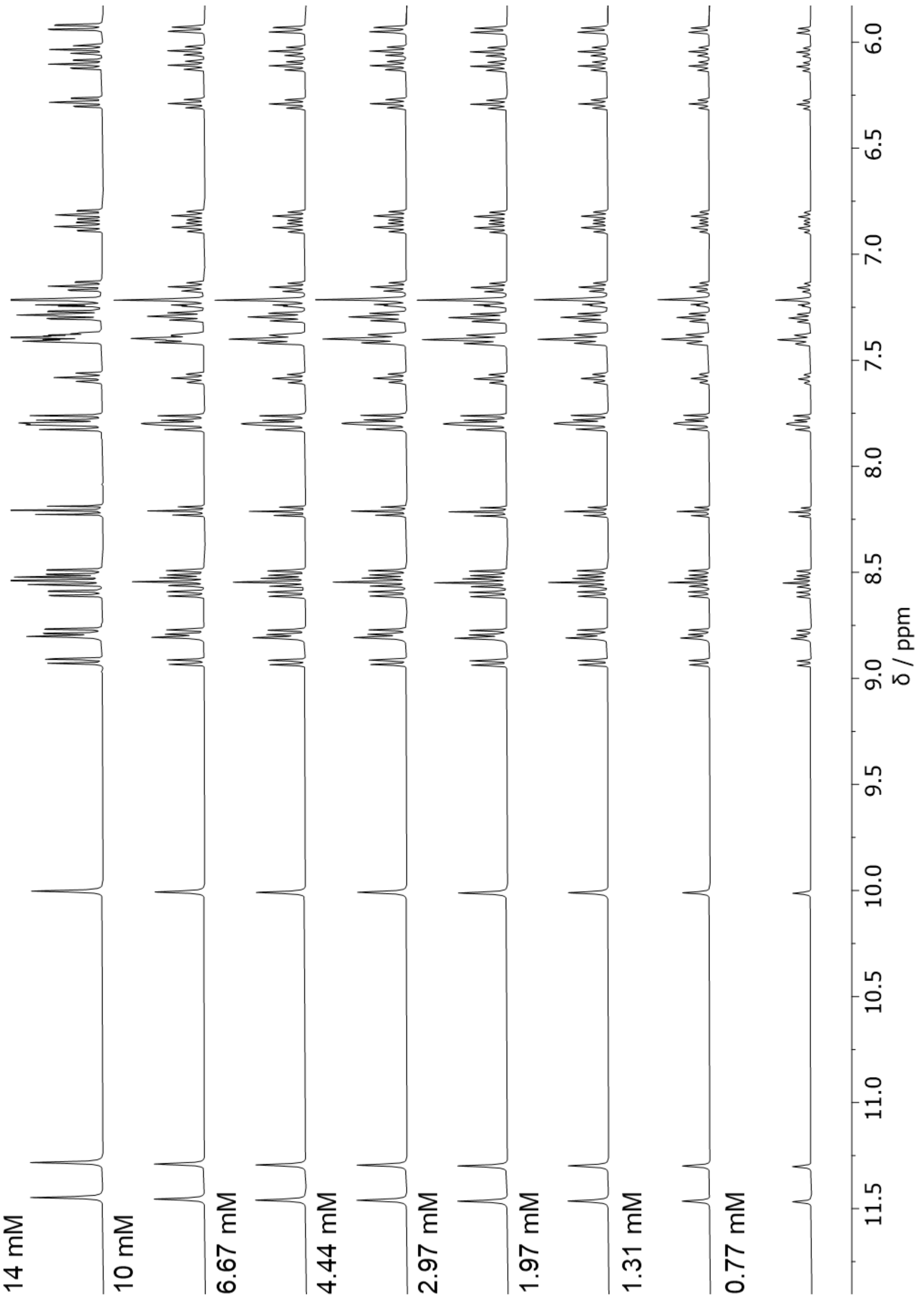

Figure S59: Dilution study showing partial ¹H NMR spectra of **1** (400 MHz, DCM-d_2, 298 K).

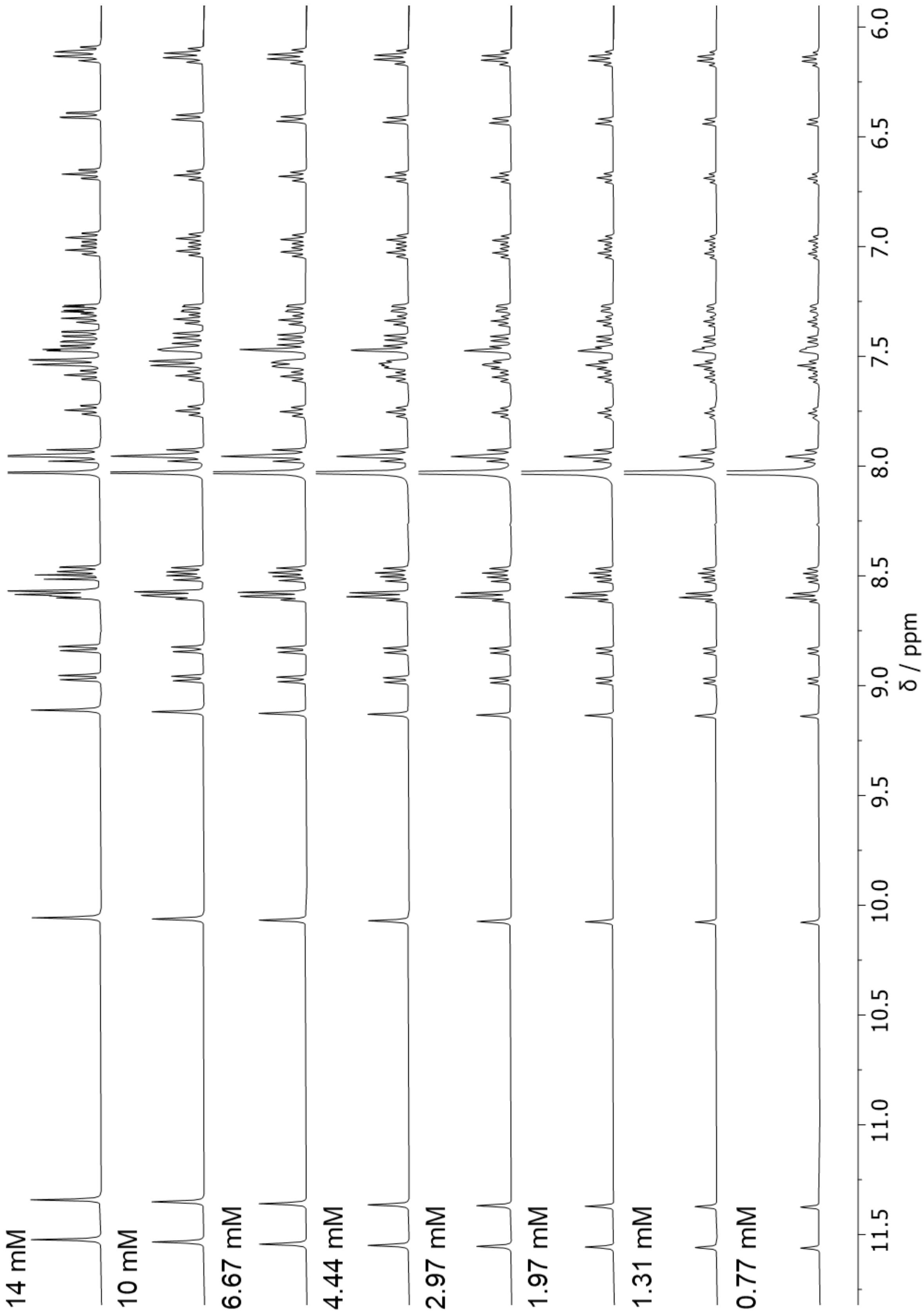

Figure S60. Dilution study showing partial ^{1}H NMR spectra of **1** (400 MHz, DMF-d_7, 298 K).

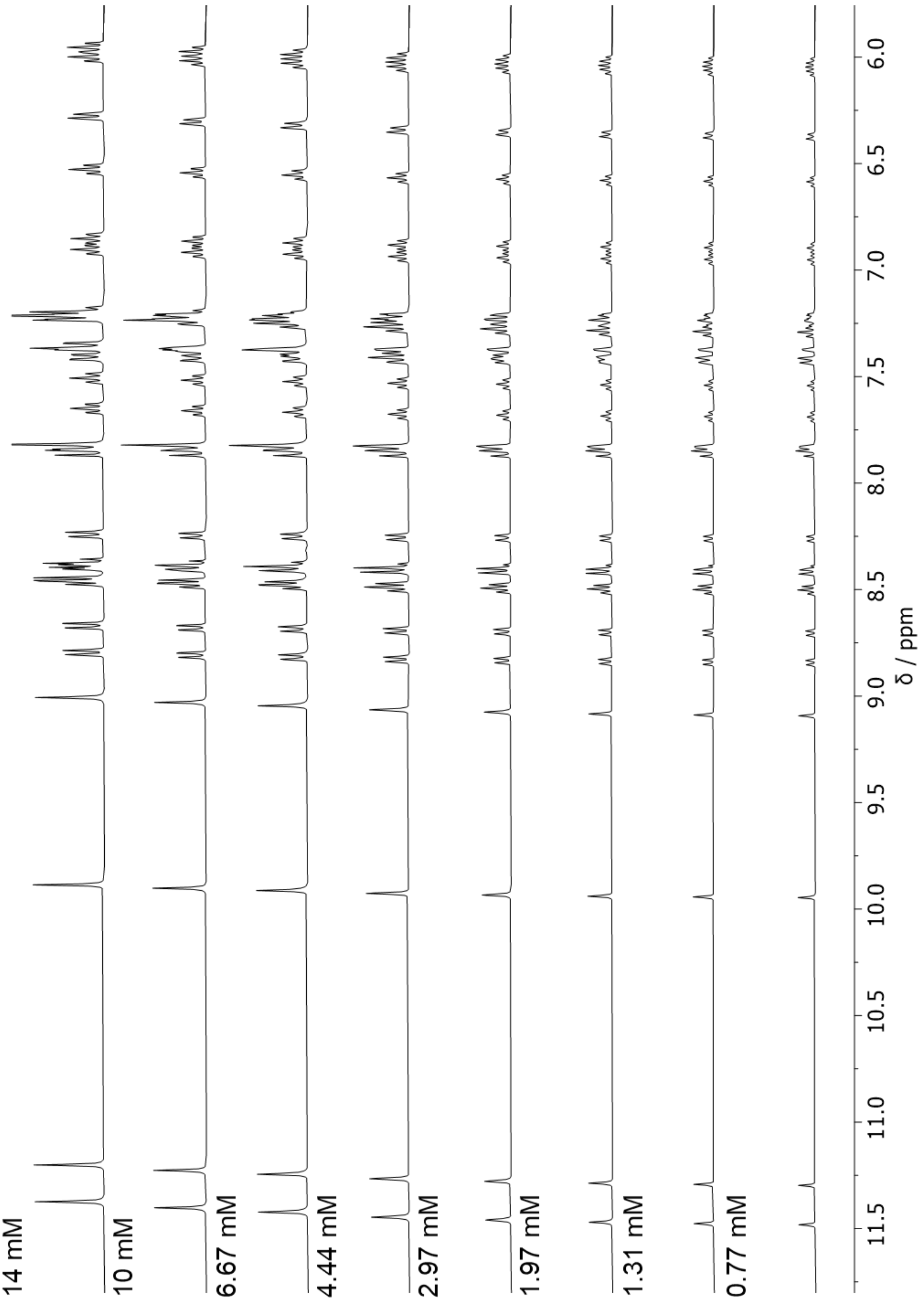

Figure S61. Dilution study showing partial ^{1}H NMR spectra of **1** (400 MHz, DMSO-d_6, 298 K).

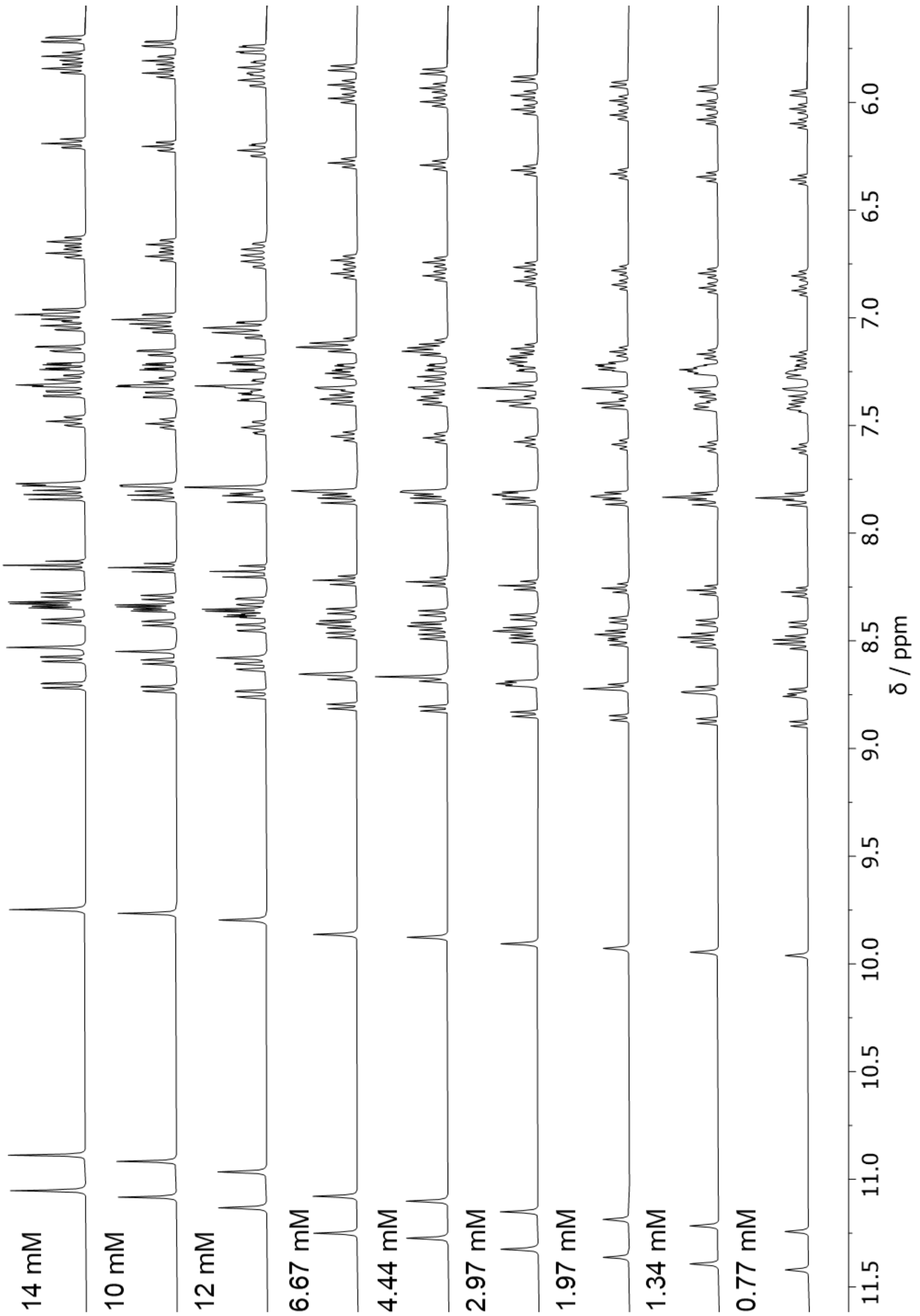

Figure S62. Dilution study showing partial ^{1}H NMR spectra of **1** (400 MHz, MeCN-d_3, 298 K).

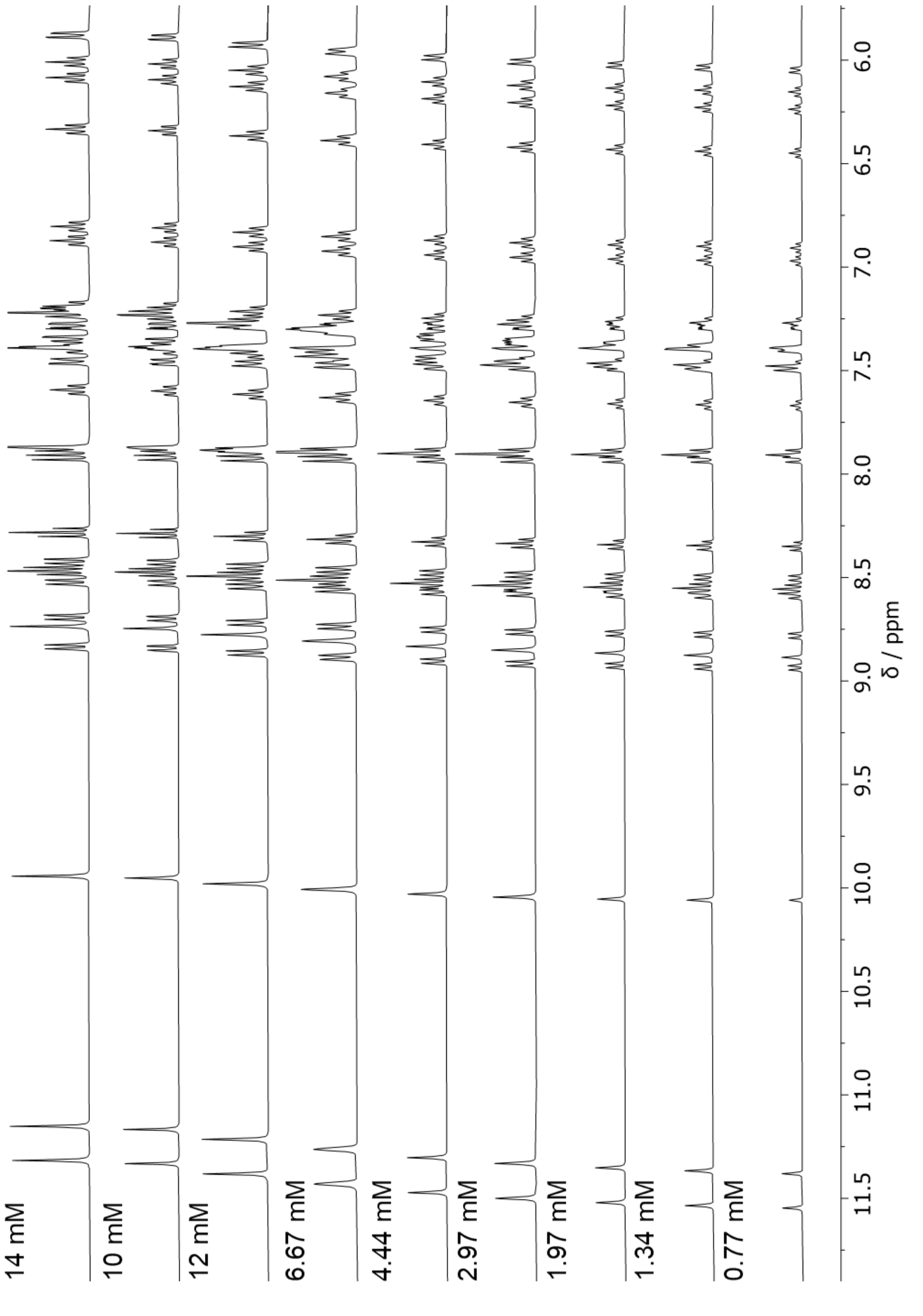

Figure S63. Dilution study showing partial ^{1}H NMR spectra of **1** (400 MHz, $MeNO_2$-d_3, 298 K).

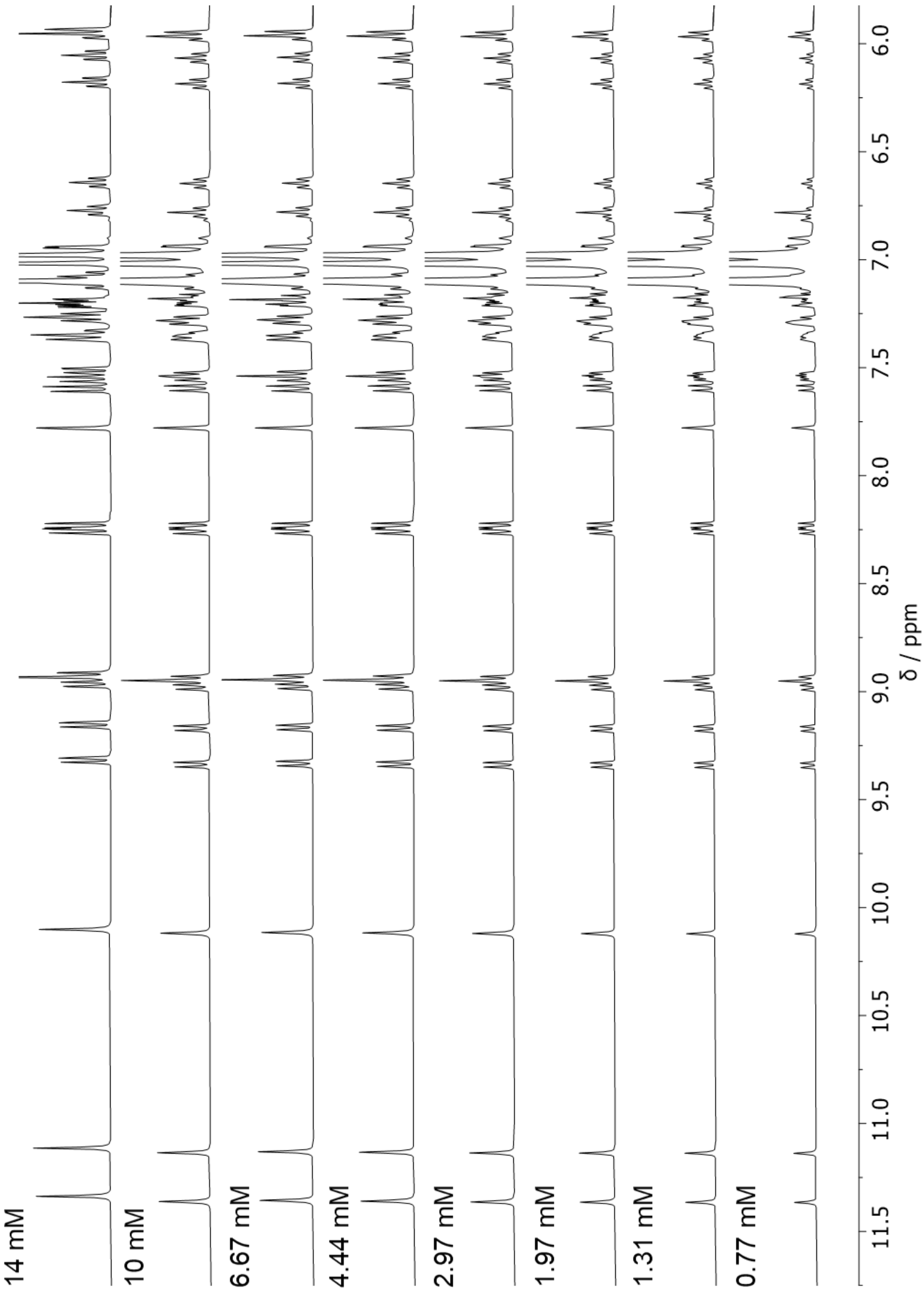

Figure S64. Dilution study showing partial ^{1}H NMR spectra of **1** (400 MHz, Toluene-d_8, 298 K).

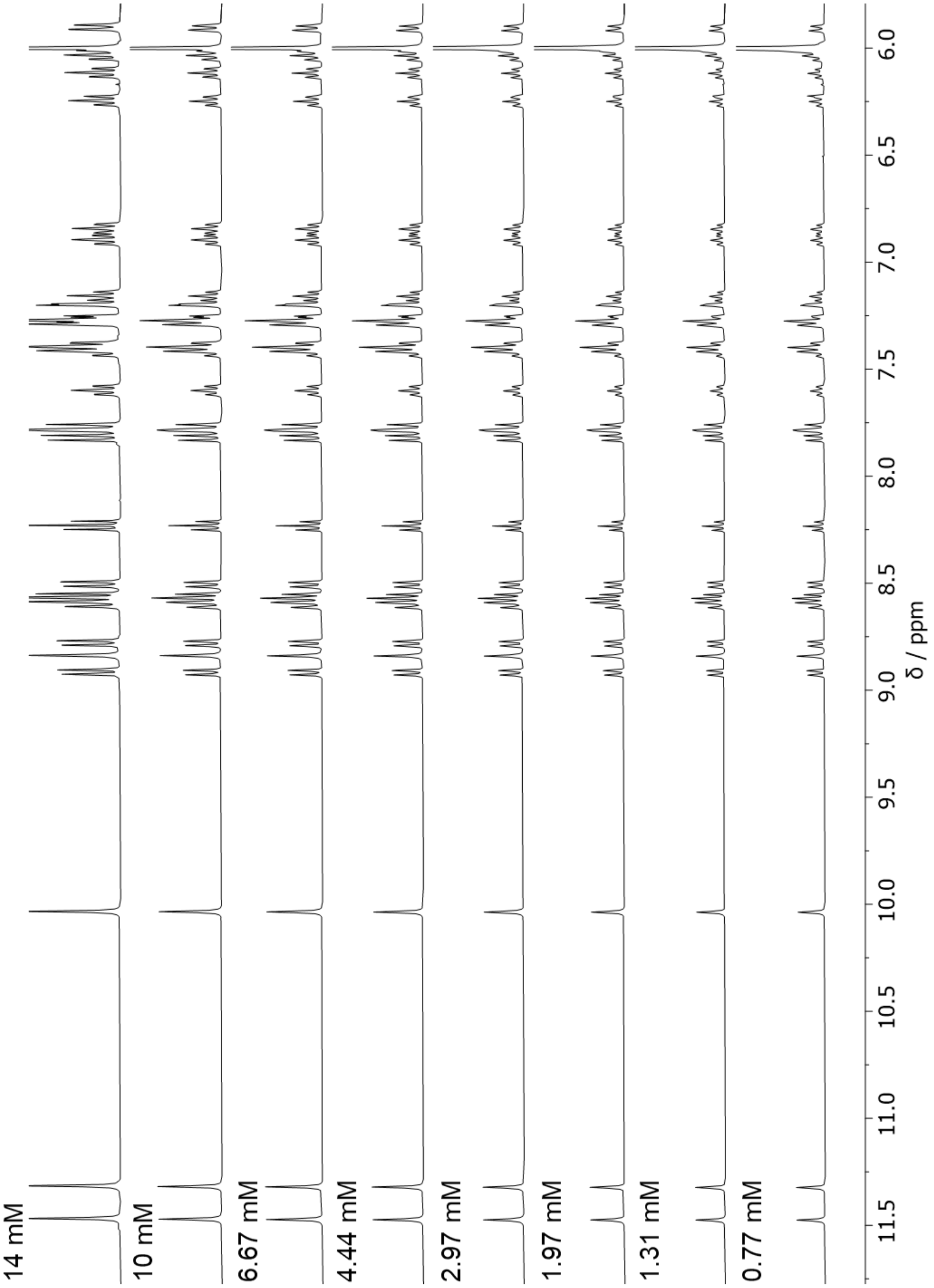

Figure S65. Dilution study showing partial ^{1}H NMR spectra of **1** (400 MHz, 1,1,2,2-Tetrachloroethane-d_2, 298 K).

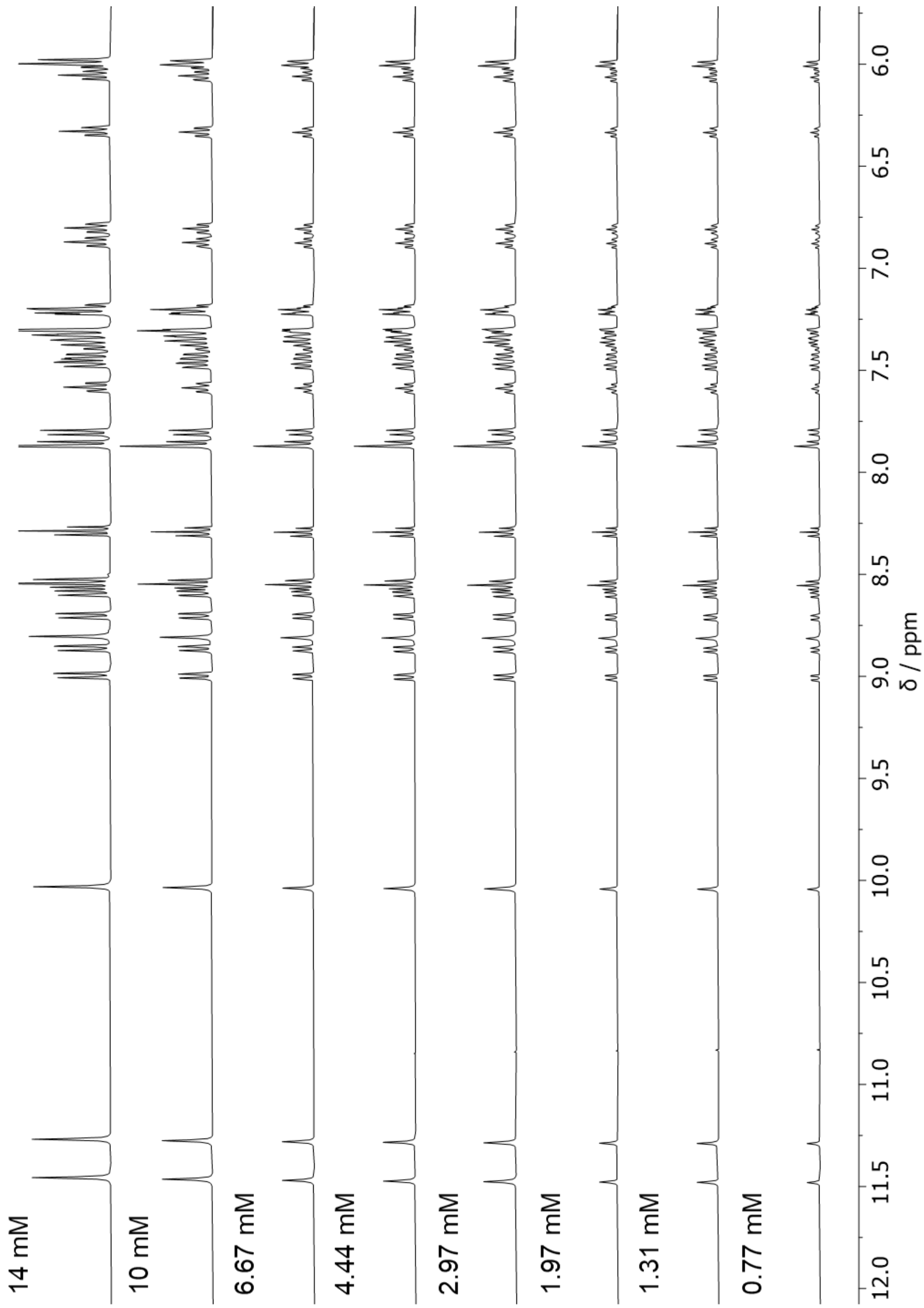

Figure S66. Dilution study showing partial ^{1}H NMR spectra of **1** (400 MHz, THF-d_8, 298 K).

^{1}H NMR Spectroscopy Dilution Experiments Summary:

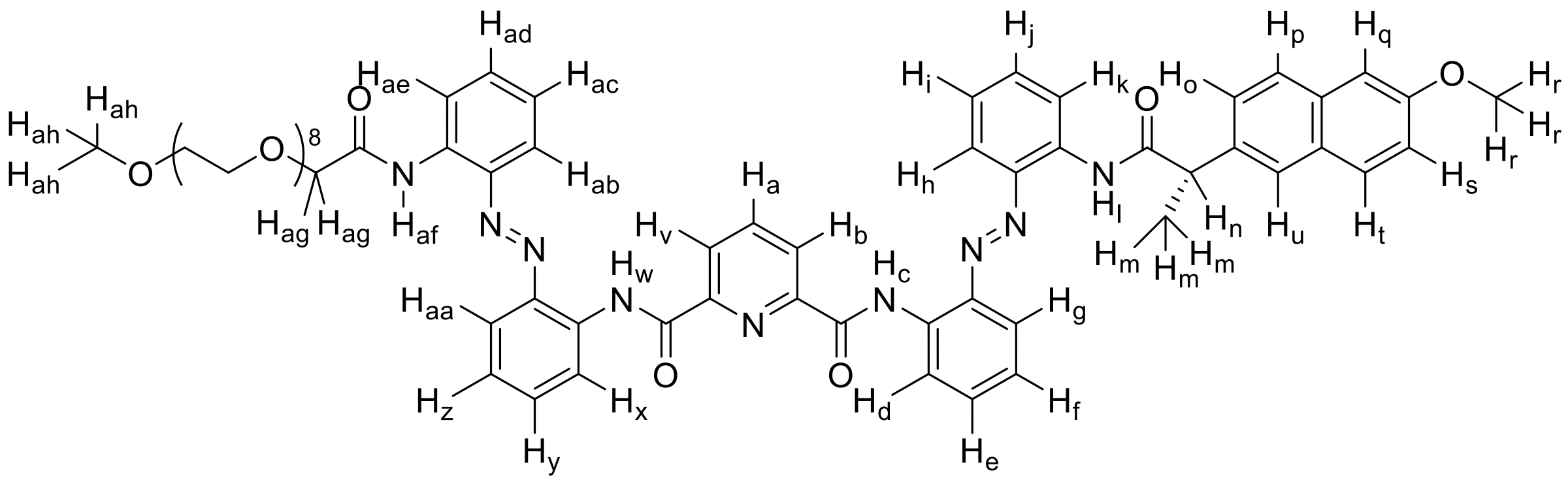

Solvent	δ/ppm at 14 mM		δ/ppm at 0.77 mM		Δ δ/ppm	
	H_w	H_c	H_w	H_c	H_w	H_c
Acetone-d_6	11.3544	11.1887	11.5105	11.3325	-0.1561	-0.1438
Benzene-d_6	11.3967	11.1779	11.4176	11.1957	-0.0209	-0.0178
CH_3CN-d_3	11.0130	10.8498	11.4208	11.2428	-0.3677	-0.3543
$CHCl_3$-d_1	11.3926	11.2427	11.4818	11.3303	-0.0892	-0.0876
CH_3NO_2-d_3	11.3171	11.1522	11.5469	11.381	-0.2298	-0.2280
DMF-d_7	11.5236	11.3418	11.5617	11.3752	-0.0381	-0.0334
DMSO-d_6	11.3740	11.2015	11.4825	11.2978	-0.1085	-0.0963
Pyridine-d_5	11.3042	11.1105	11.3210	11.1250	-0.0168	-0.0145
TCE-d_2	11.4679	11.3158	11.4758	11.3236	-0.0079	-0.0078
THF-d_8	11.4565	11.2686	11.4808	11.2899	-0.0243	-0.0213
Toluene-d_8	11.3370	11.1139	11.3656	11.1385	-0.0286	-0.0246

Bindfit data for **1** in Acetone-d_6

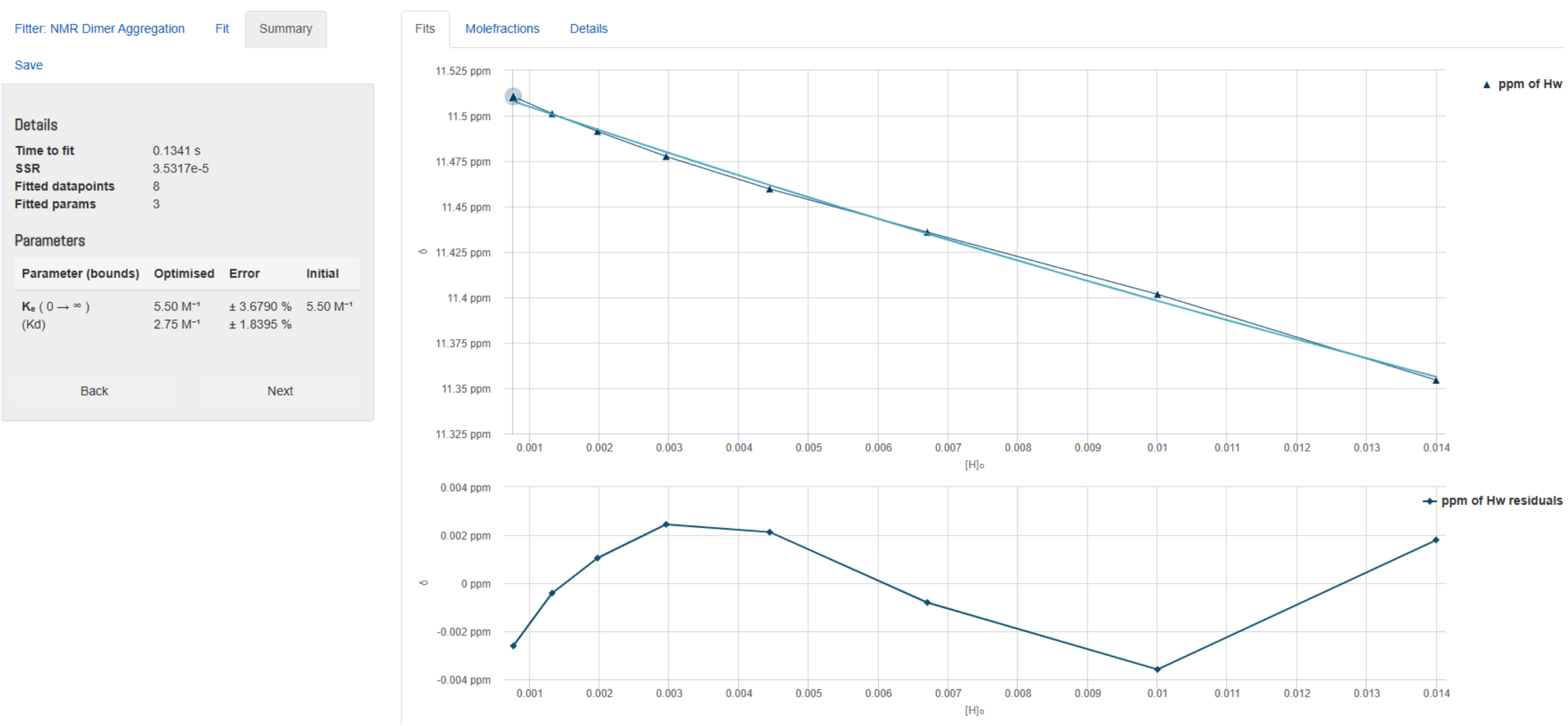

Figure 67: Bindfit for ^{1}H NMR spectroscopy dilution data of **1** in acetone-d_6 using a dimer aggregation model.

Bindfit data for **1** in CH_3CN-d_3

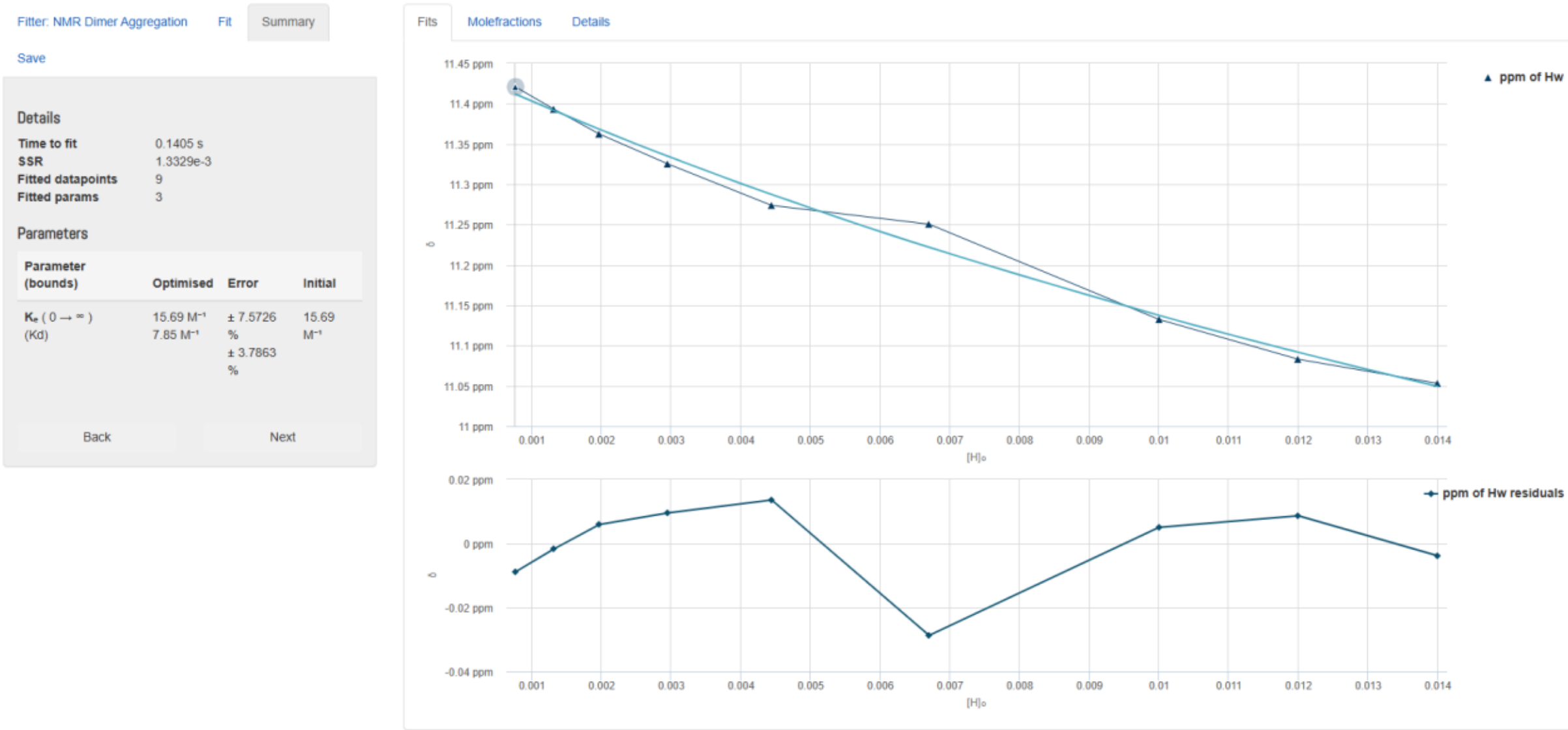

Figure 68: Bindfit for ^{1}H NMR spectroscopy dilution data of **1** in CH_3CN-d_3 using a dimer aggregation isotherm.

Bindfit data for **1** in CH_3NO_2-d_3

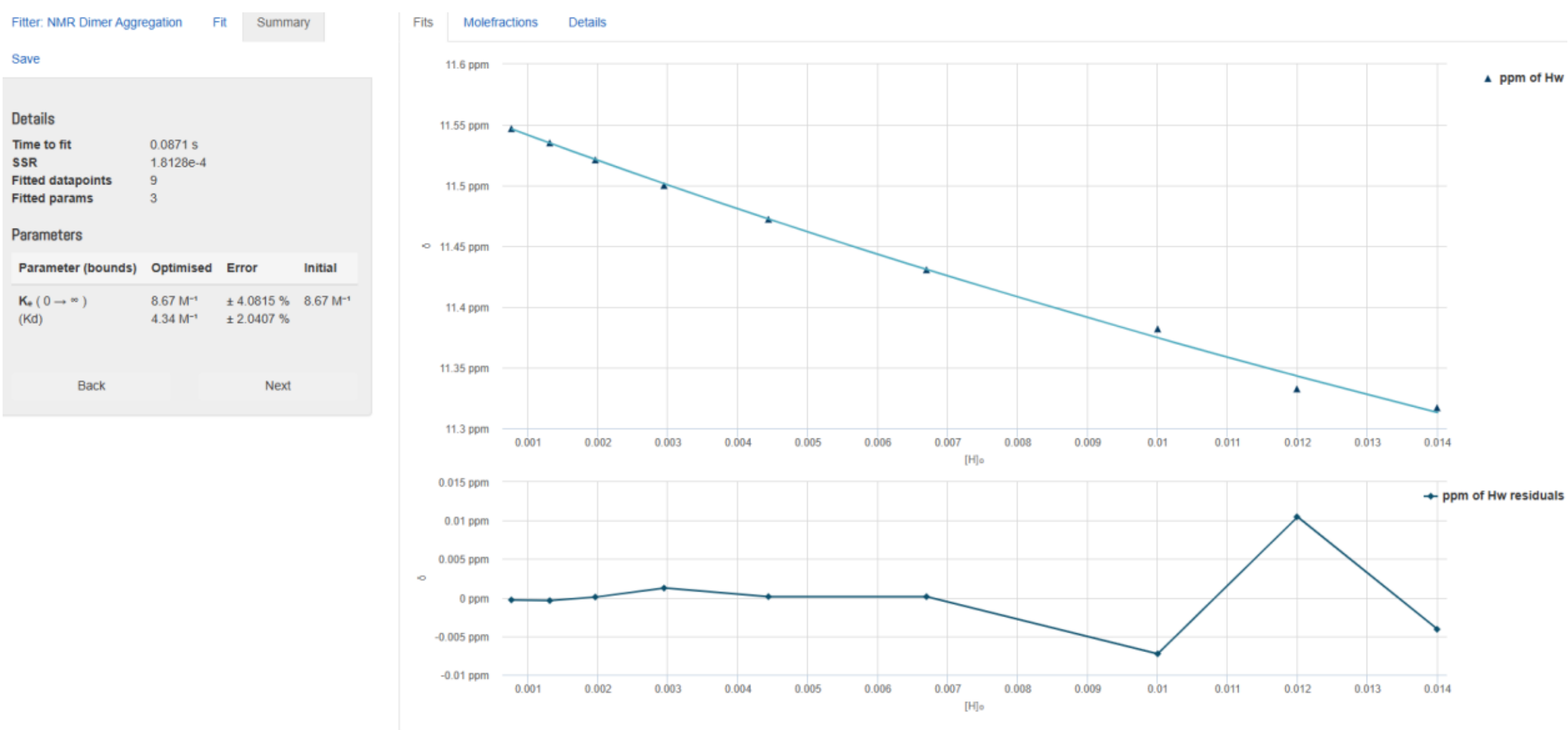

Figure 69: Bindfit for ^{1}H NMR spectroscopy dilution data of **1** in CH_3NO_2-d_3 using a dimer aggregation isotherm.

Bindfit data for **1** in DMSO-d_6

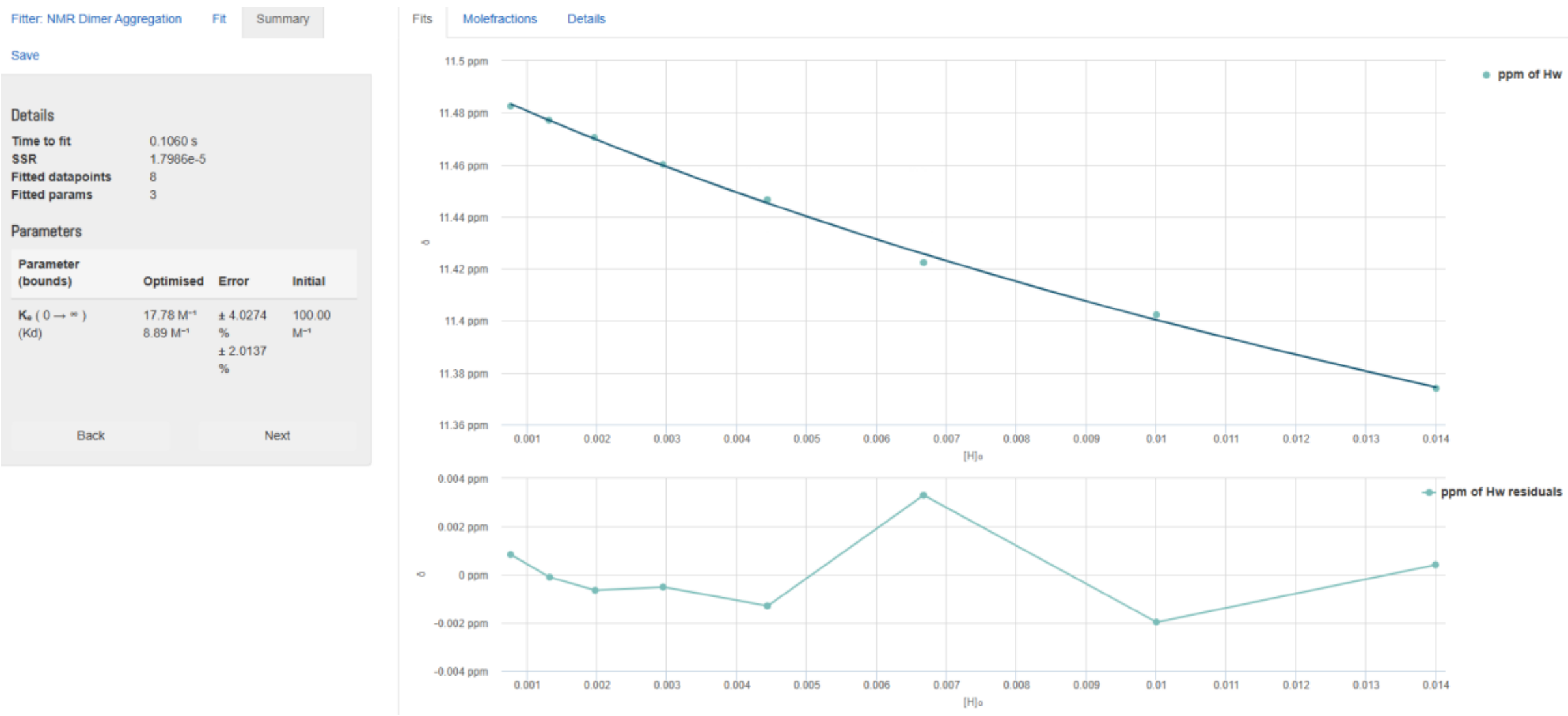

Figure 70: Bindfit for ^{1}H NMR spectroscopy dilution data of **1** in DMSO-d_6 using a dimer aggregation isotherm.

IR Spectroscopic Analysis

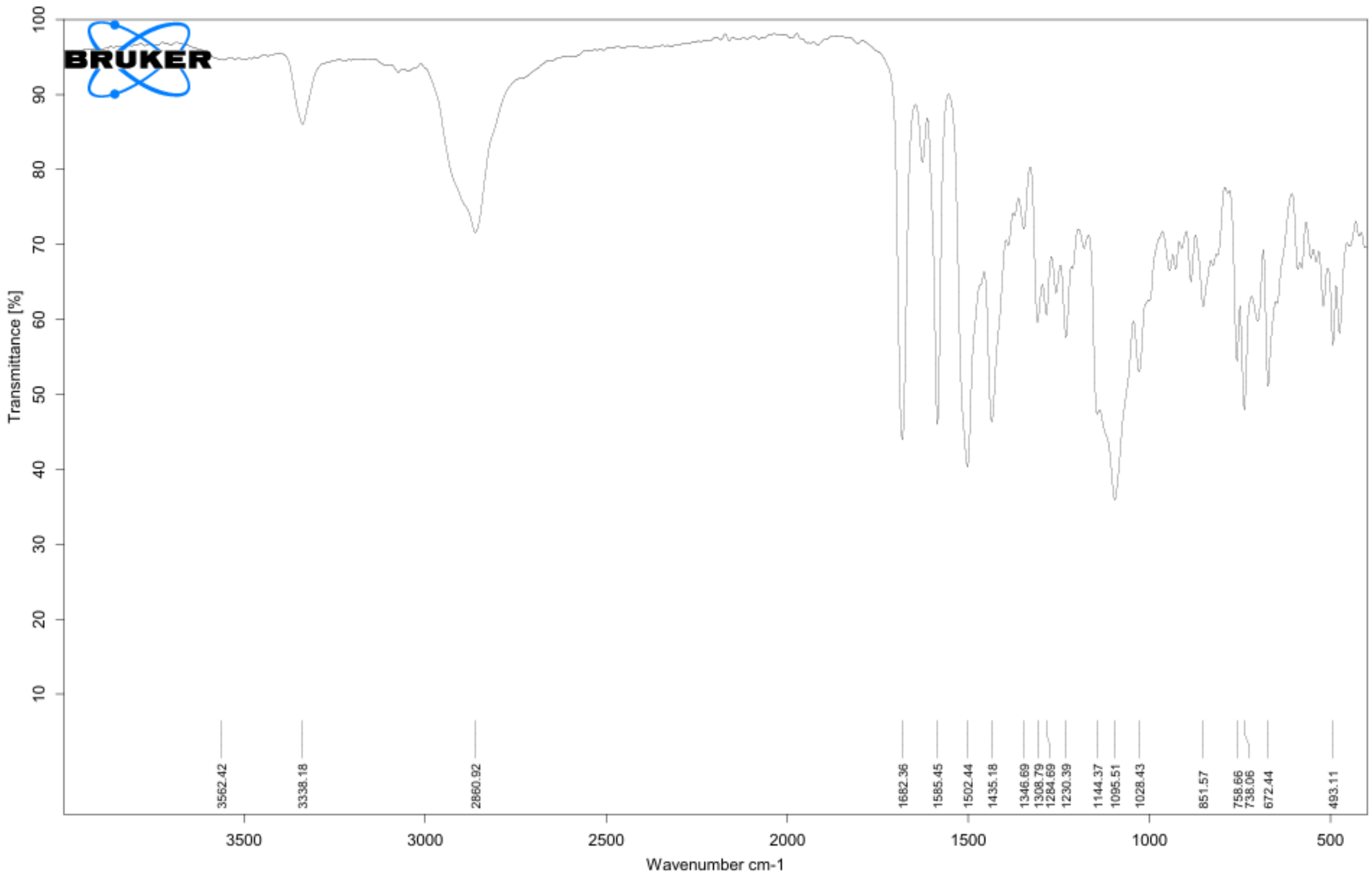

Figure S71: IR Spectrum of **1**.

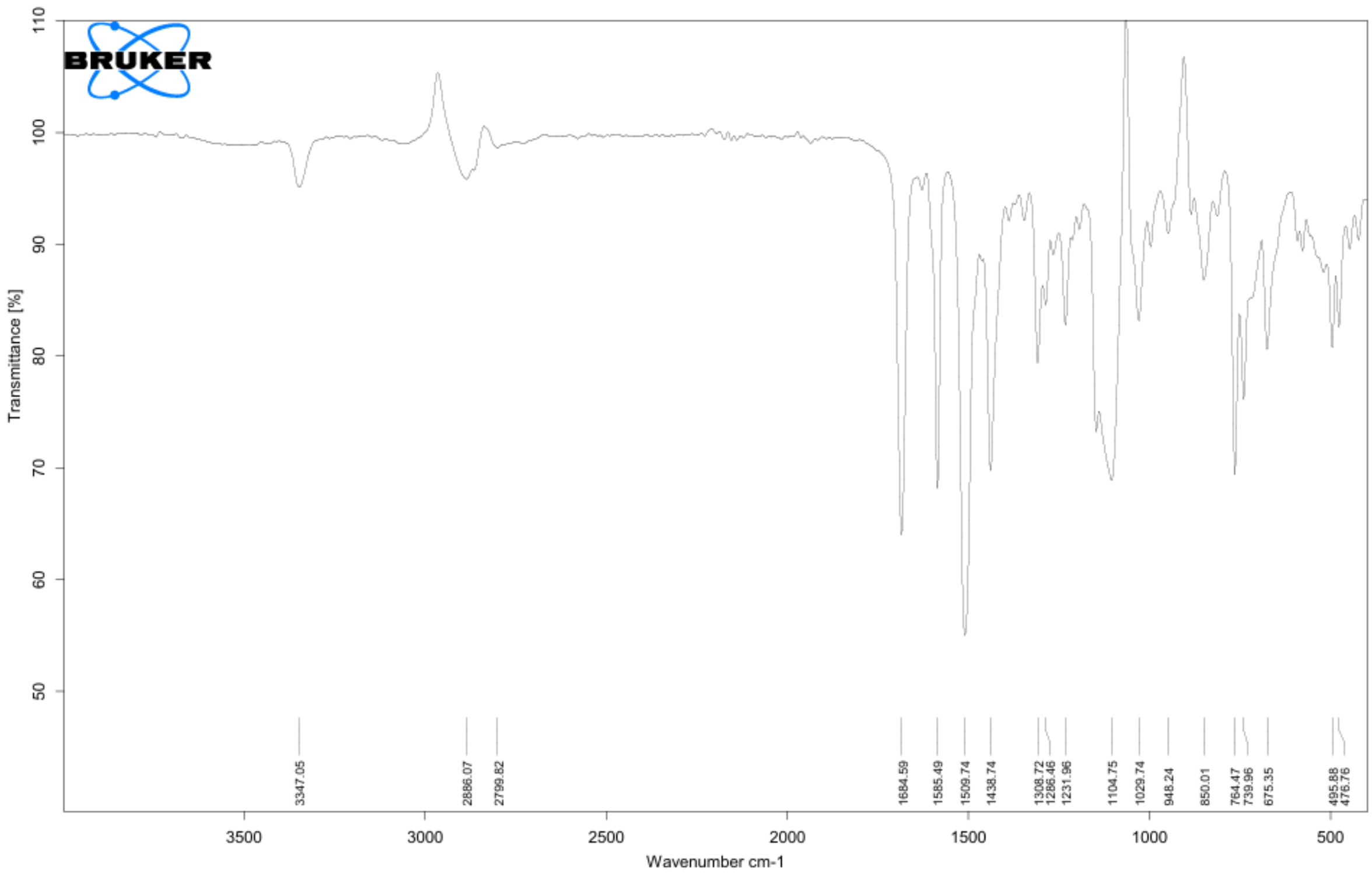

Figure S72 IR Spectrum of **1** dissolved in THF.

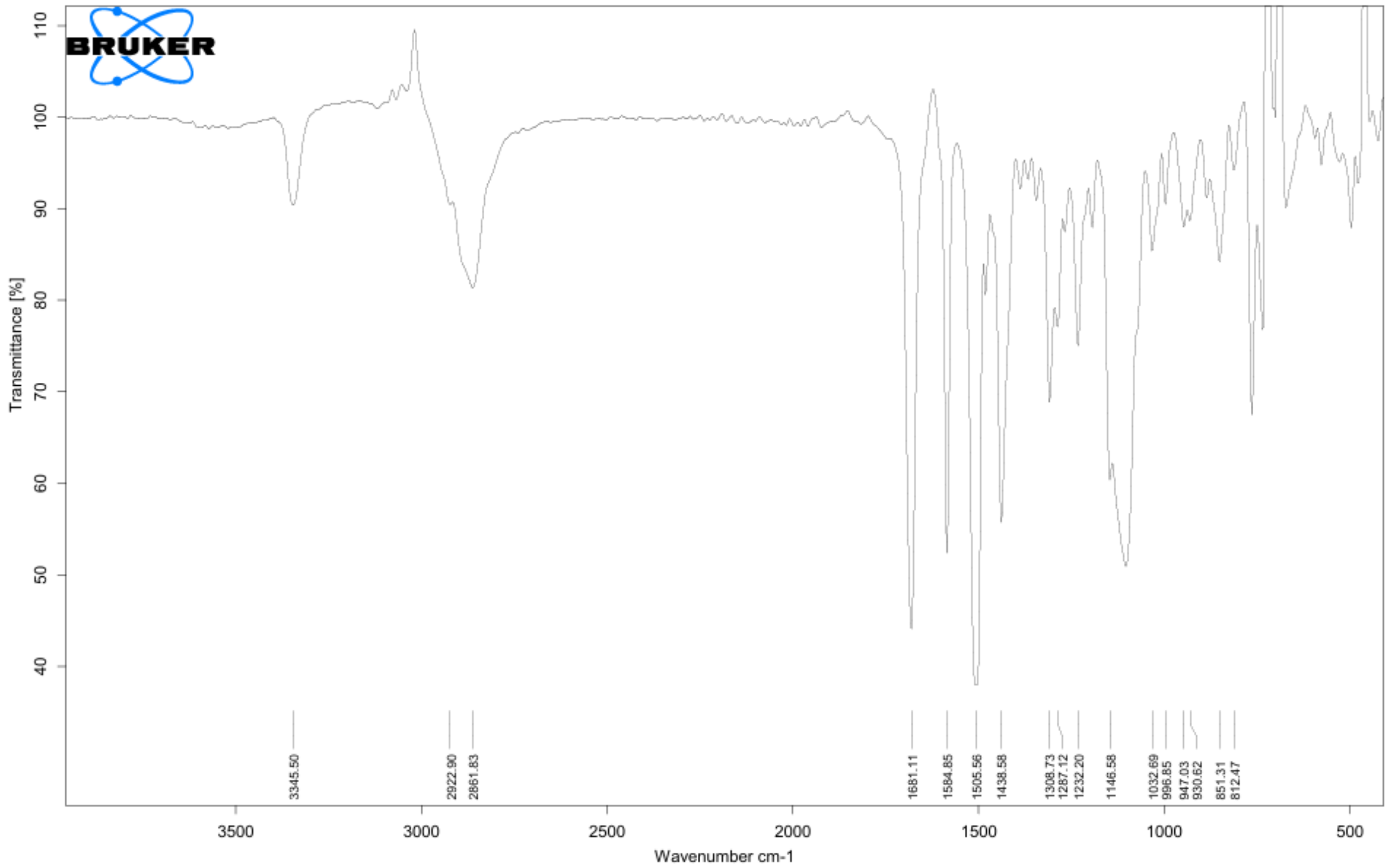

Figure S73: IR Spectrum of **1** dissolved in toluene.

1584

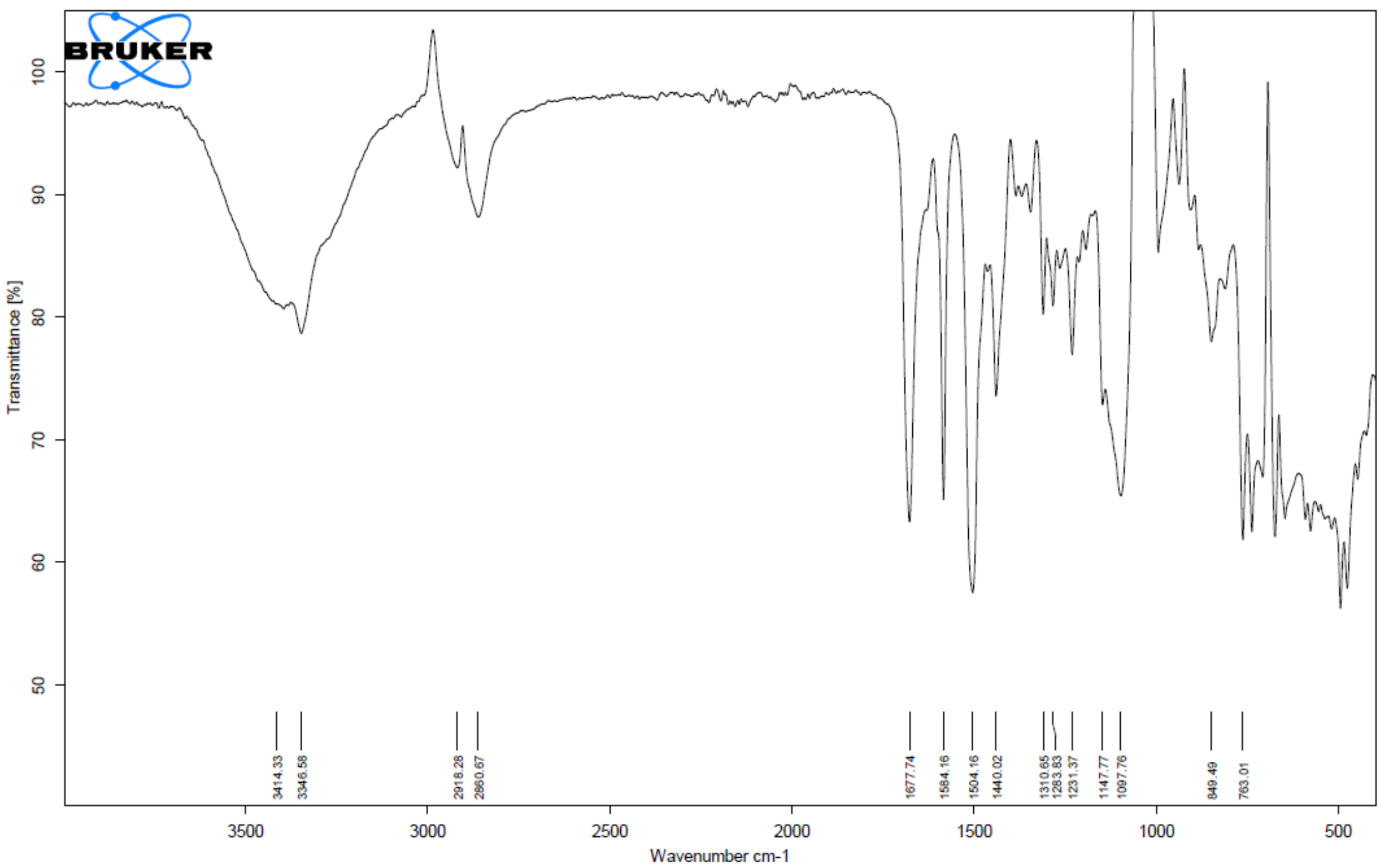

Figure S74: IR Spectrum of **1** dissolved in DMSO .

IR Spectroscopy Summary

Table S2. Summarising wavenumbers of IR bands of **1** taken in the solid state and different solvents.

Wavenumber/ cm^{-1}			
**1** Solid	**1** in THF	**1** in Toluene	**1** in DMSO
3338.18	3347.05	3345.50	3346.58[a]
2860.92	2886.07	2861.83	2860.67
1682.36	1684.59	1681.11	1667.74
1585.45	1585.49	1584.85	1584.16
1502.44	1509.74	1505.56	1504.16
1435.18	1438.74	1438.58	1440.02
1308.79	1308.72	1308.73	1310.65
1284.69	1286.46	1287.12	1283.83
1230.39	1231.96	1232.20	1231.37

[a]The absorption peaks above 3300 cm^{-1} are likely slightly obscured.

Computational Studies

Method. Calculations were run in Orca v6.0.1.[55] Input geometries for the foldamer core were taken from the crystal structure (**4**), with the alkyl chain added manually in Avogadro. The geometry was relaxed at B97-D4/6-31+G** level of theory with energy convergence threshold of 1E-6 Ha, displacements of 2E-3 Bohr, and max gradient errors of 3E-4 Ha.[43,44] The optimised geometry was used as input for exploring the effects of solvation using the polarised continuum model (PCM),[45] as implemented in Orca v6.0.1.

Optimised Geometry

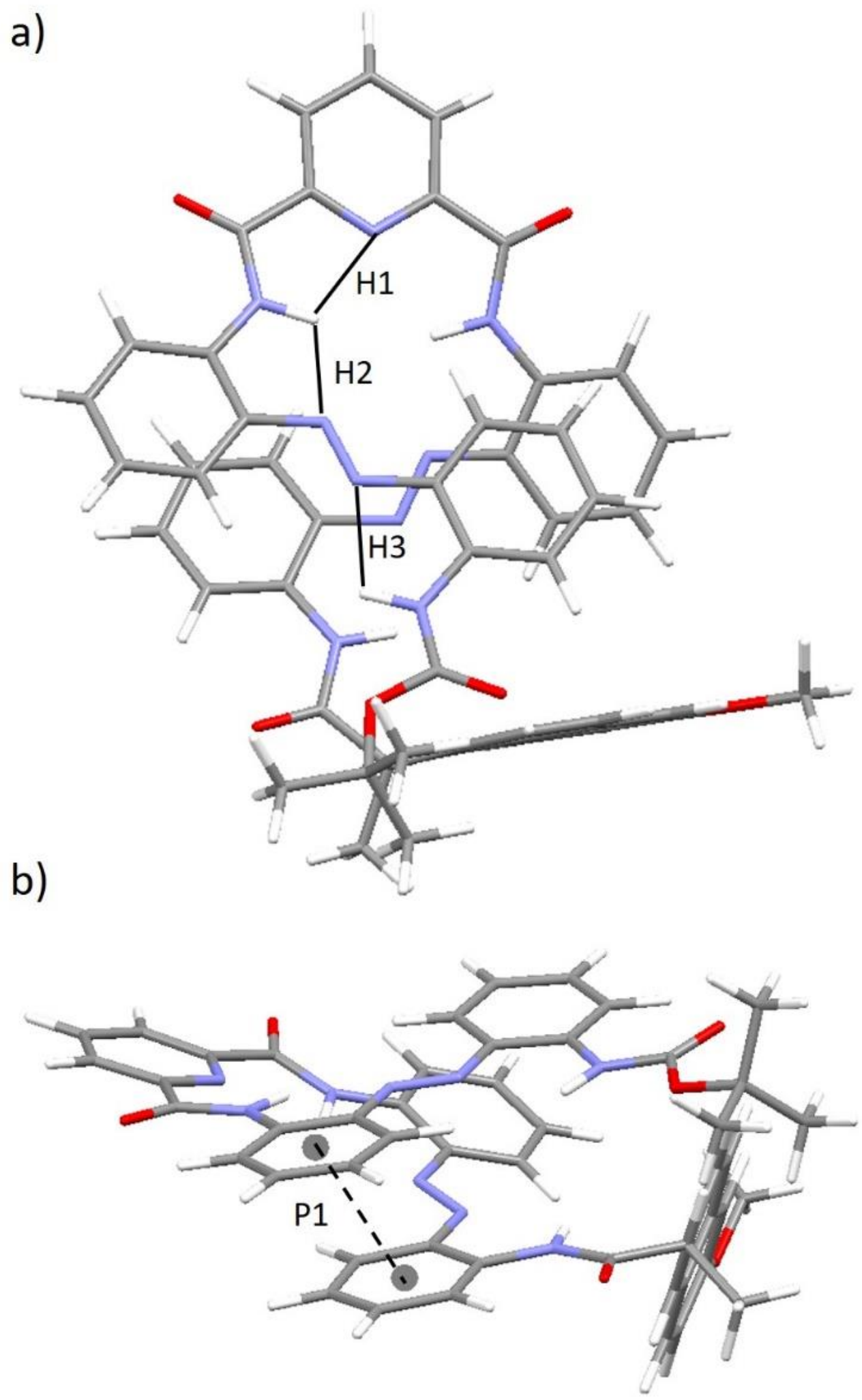

Figure S75. Optimised geometry of the foldamer core using DFT calculations. In a) the black lines highlight the calculated bond lengths for the three distinct intramolecular NH···N hydrogen bonds and NH···N close contacts involving the pyridine, azo and amide groups found in the foldamer core; b) the dashed black line highlights the centroid-centroid distance measured computationally to gain insights into the intramolecular π-π stacking interactions found between two aromatic rings in the foldamer core.

Table S3. Calculated bond lengths of different bonds in the gas phase optimisation of 4. Atom labels match those give above in Figure S75.

**Atom label**	**Bond Length / Å**
H1 **(H…N)**	2.135
H2 **(H…N)**	2.196
H3 **(H…N)**	2.147
P1 (centroid…centroid)	3.743

Polarised Continuum Model

Table S4 Calculated bond lengths of different intermolecular interactions when the geometry of **4** was relaxed in the presence of an implicitly added solvent, using the polarised continuum model for chloroform ($CHCl_3$), dimethylsulfoxide (DMSO), methanol (MeOH), tetrahydrofuran (THF), toluene and water (H_2O). Dielectric constants of each solvent are also shown. Atom labels match those give above in Figure S75.

	**Bond Length /Å**					
**Atom Labels**	**$CHCl_3$** ($\epsilon = 5$)	**DMSO** ($\epsilon = 47$)	**MeOH** ($\epsilon = 33$)	**THF** ($\epsilon = 8$)	**Toluene** ($\epsilon = 2$)	**H_2O** ($\epsilon = 80$)
H1 **(H…N)**	2.138	2.144	2.144	2.139	2.137	2.144
H2 **(H…N)**	2.176	2.172	2.173	2.175	2.185	2.172
H3 **(H…N)**	2.191	2.203	2.202	2.195	2.172	2.203
P1 (centroid…centroid)	3.720	3.720	3.720	3.719	3.723	3.720

Isomerisation + Internal Energy (Core molecule, without chain)

To assess the difference between the relative energies of the different isomers of the foldamer, the internal energy of the core of the molecule (i.e., the foldamer scaffold) has been calculated for **3** in the gas phase and using PCM as outlined above for toluene, chloroform, tetrahydrofuran, methanol, dimethylsulfoxide and water.

Table S5 Calculated internal energies for the (*S*)- and (*R*)-isomer of **3** in the gas phase and using PCM as outlined above for toluene, chloroform, tetrahydrofuran, methanol, dimethylsulfoxide and water.

**Solvent**	**Energy _ Isomer 1 (S)**	**Energy_Isomer 2 (R)**	$\Delta E(S-R)$ **/eV**
Gas	-2876.0709	-2876.0630	-0.2147
Toluene	-2876.0925	-2876.0841	-0.2269
$CHCl_3$	-2876.1018	-2876.0935	-0.2236
THF	-2876.1048	-2876.0966	-0.2221
MeOH	-2876.1099	-2876.1018	-0.2180
DMSO	-2876.1104	-2876.1023	-0.2193
Water	-2876.1108	-2876.1028	-0.2169

Influence of PEG Chain:

Addition of PEG chain: The PEG chain was attached to the optimised cores, obtained in the above computational studies. Notably, at DFT level it is not trivial to explore the full orientational space of the alkyl chain, with geometry optimisations driving the system to the local minimum state. Therefore it is anticipated that small variations in the initial chain geometry will lead to different final configurations of the alkyl chain, making it challenging to directly compare the energies between different structures. However, direct comparison the energetics of the gas-phase and solvated geometries can be undertaken without this issue

Notably, with the DFT optimisation, the chain remains in an extended conformation, (Figure S76, and therefore does not affect the foldamer core. To better understand how the chain interacts with the foldamer core, we performed a conformational screening at GFN-XTB level using the global geometry optimisation algorithm (GOAT) as implemented in ORCA 6.0, Figure S76 and Figure S77. In this simulation, the foldamer core was fixed in place at the DFT-optimised geometry, with the algorithm allowed to explore the conformational flexibility of the tail. As is evident from this analysis, the chain coils beneath the foldamer core to maximise enthalpic stabilisation of the chain, but does not otherwise contribute to interactions within the foldamer core itself. We therefore anticipate that one can compare the relative energies of the foldamer cores, in the absence of the chain, as a good approximation for the relative stabilities of the system.

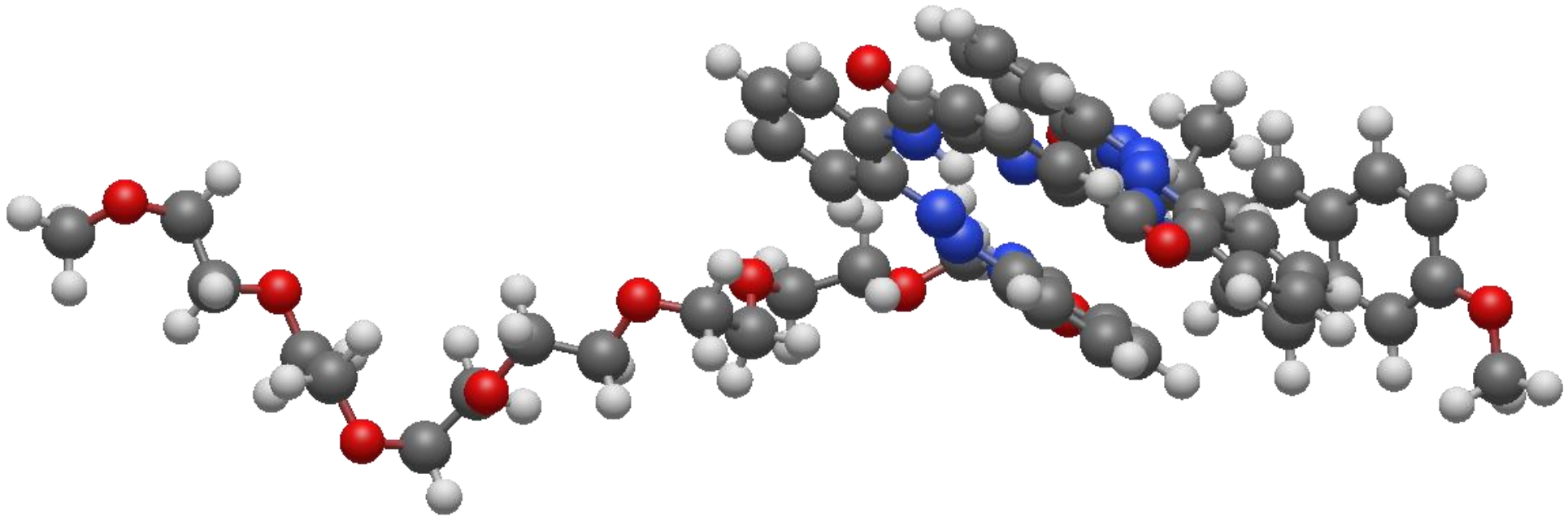

Fig S76. Optimised (DFT) gas-phase geometry of the foldamer core with full chain.

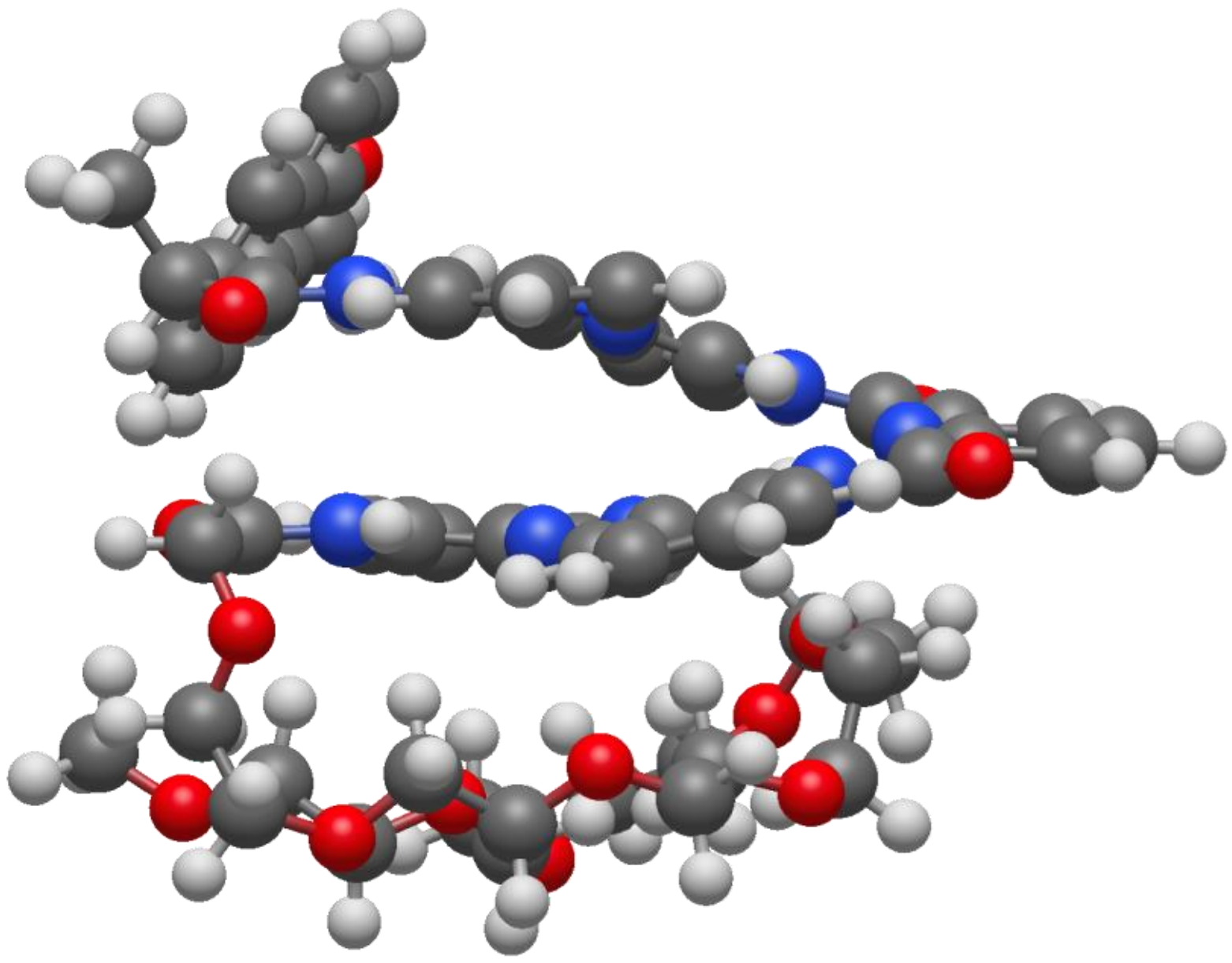

Fig S77. Global minimum geometry for the gas-phase foldamer obtained at GFN-XTB level of theory.

Appendix: ^{1}H and ^{13}C NMR Spectra

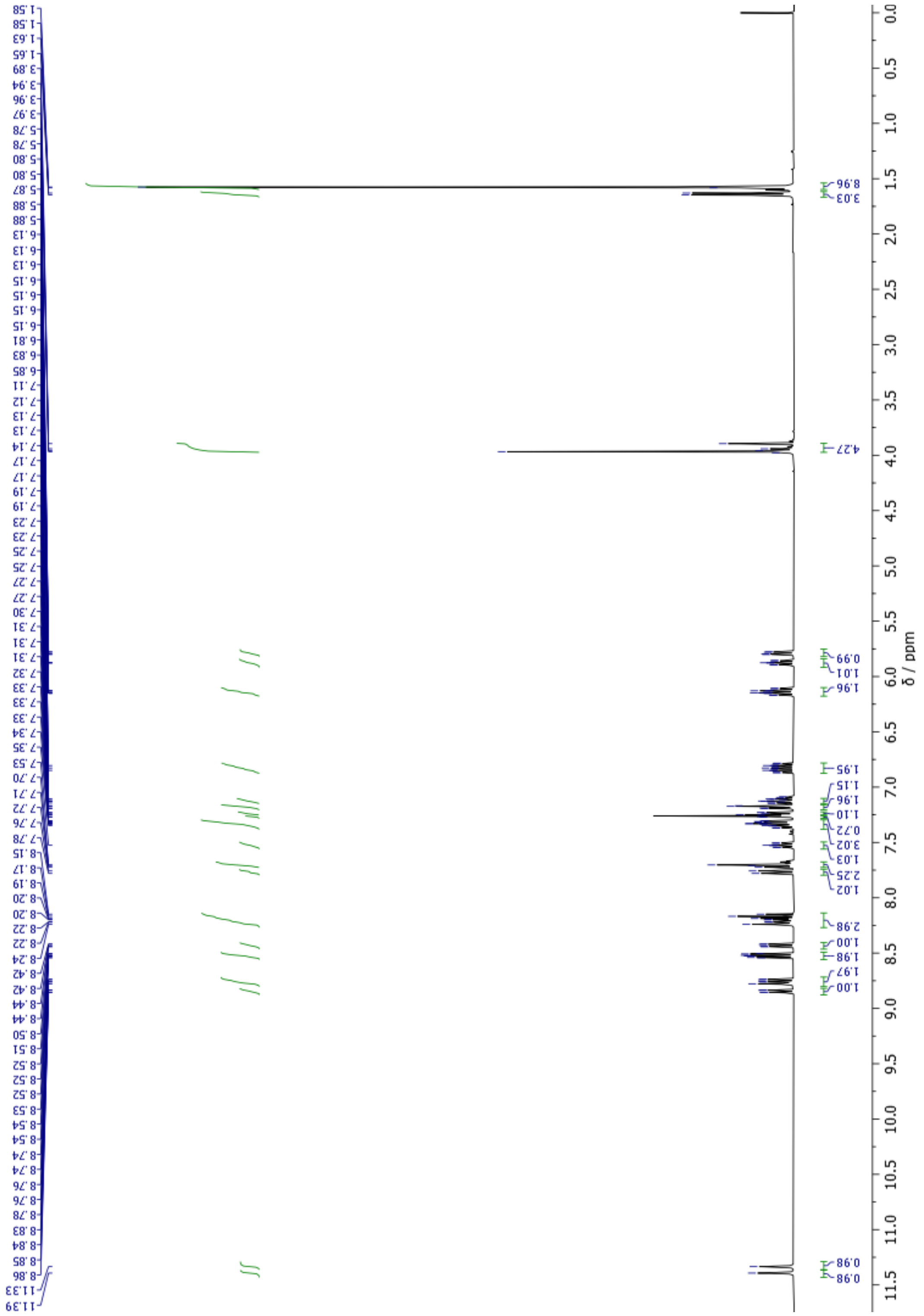

^{1}H NMR spectrum (400 MHz, $CDCl_3$, 298 K) of **4**

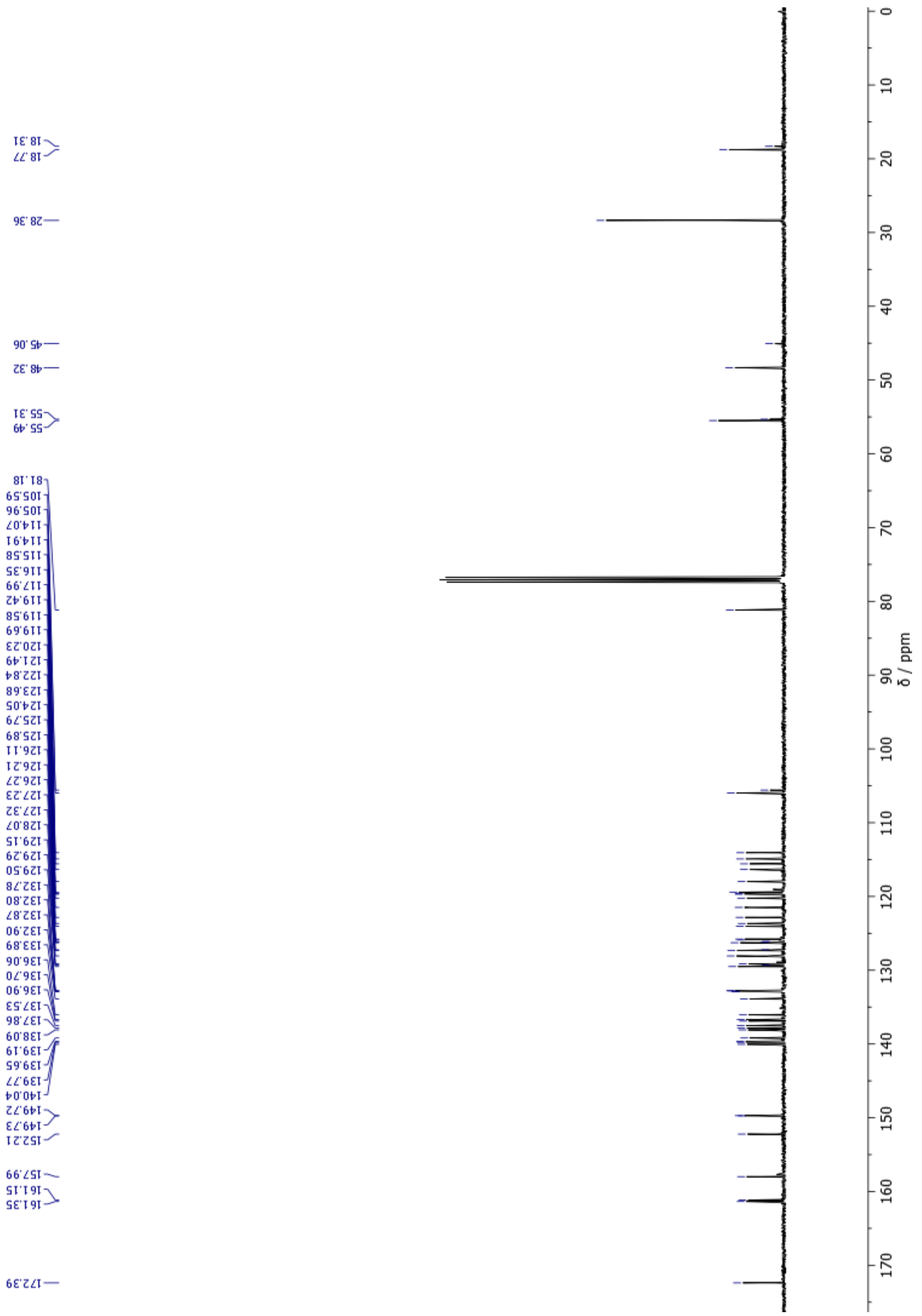

^{13}C NMR spectrum (101 MHz, $CDCl_3$, 298 K) of **4**

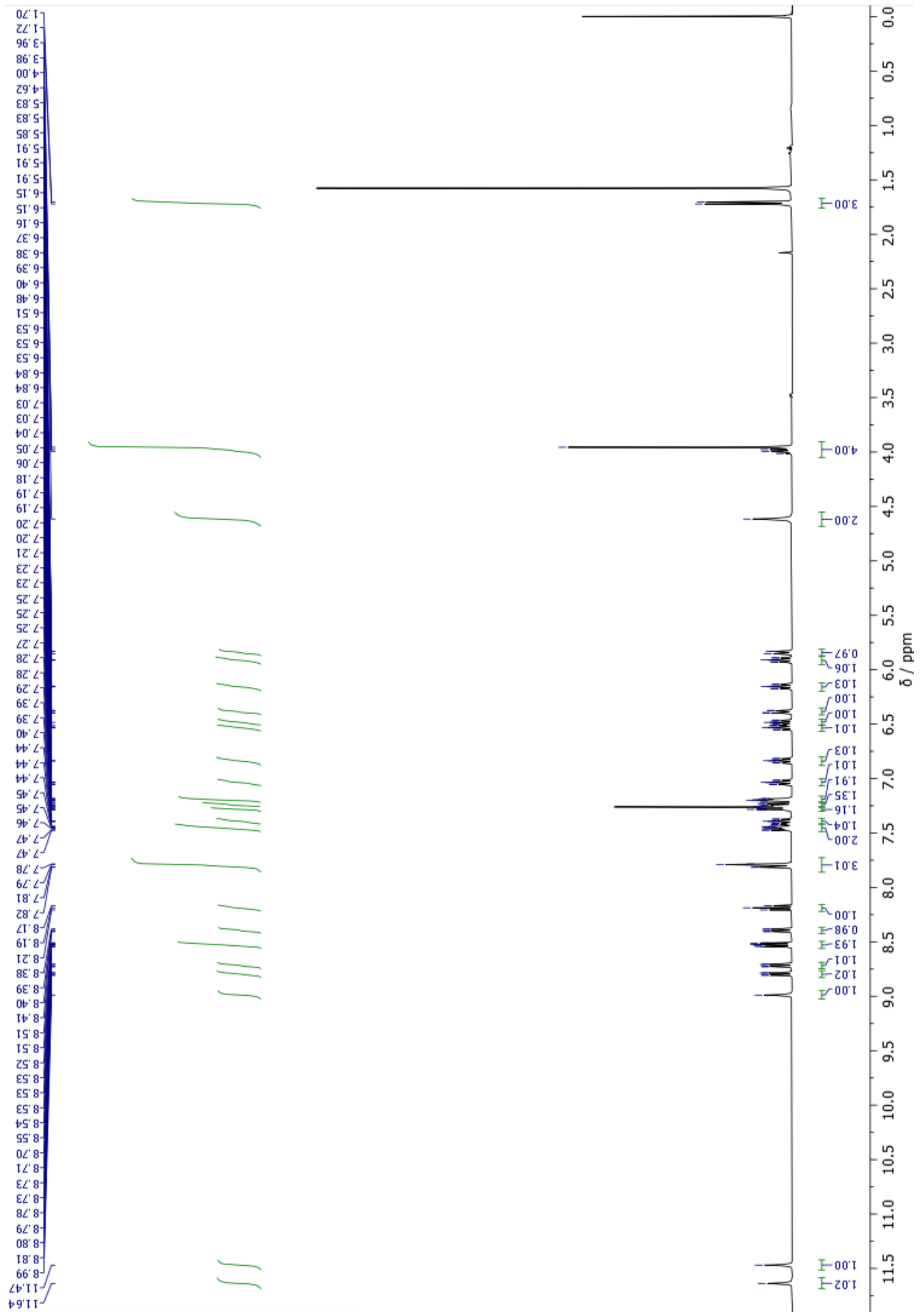

^{1}H NMR spectrum (400 MHz, $CDCl_3$, 298 K) of **5**

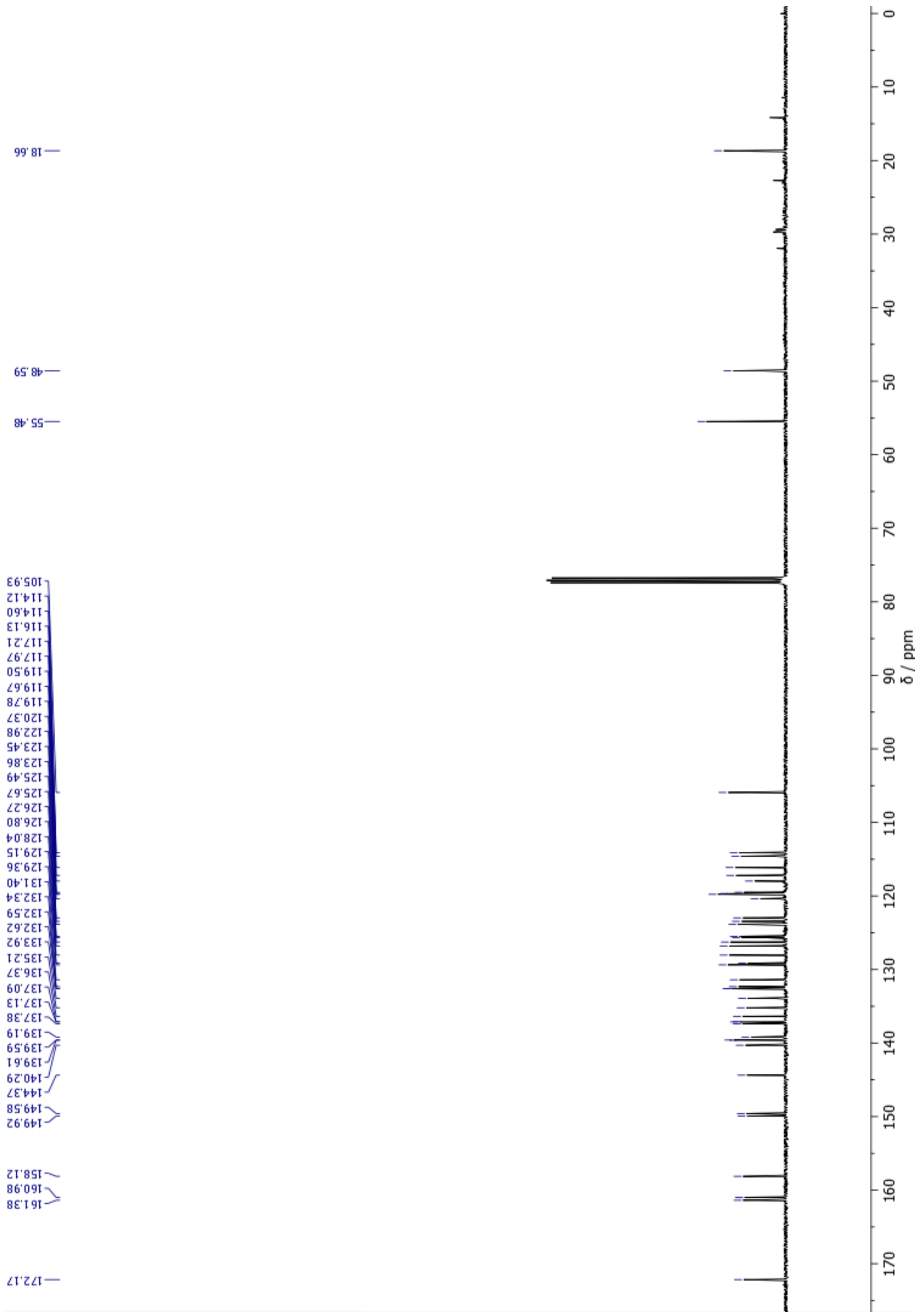

^{13}C NMR spectrum (101 MHz, $CDCl_3$, 298 K) of **5**

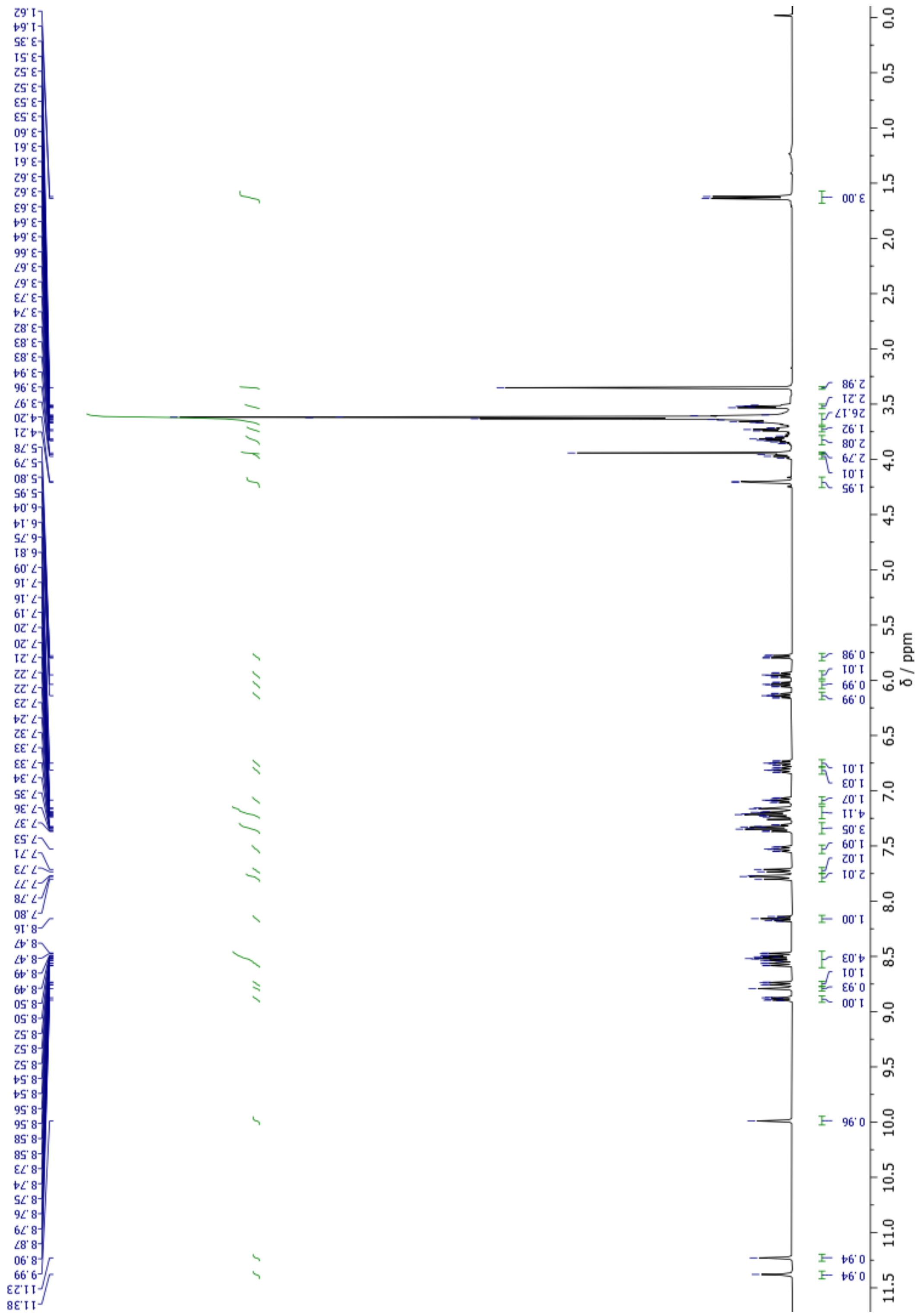

^{1}H NMR spectrum (400 MHz, $CDCl_3$, 298 K) of **1**

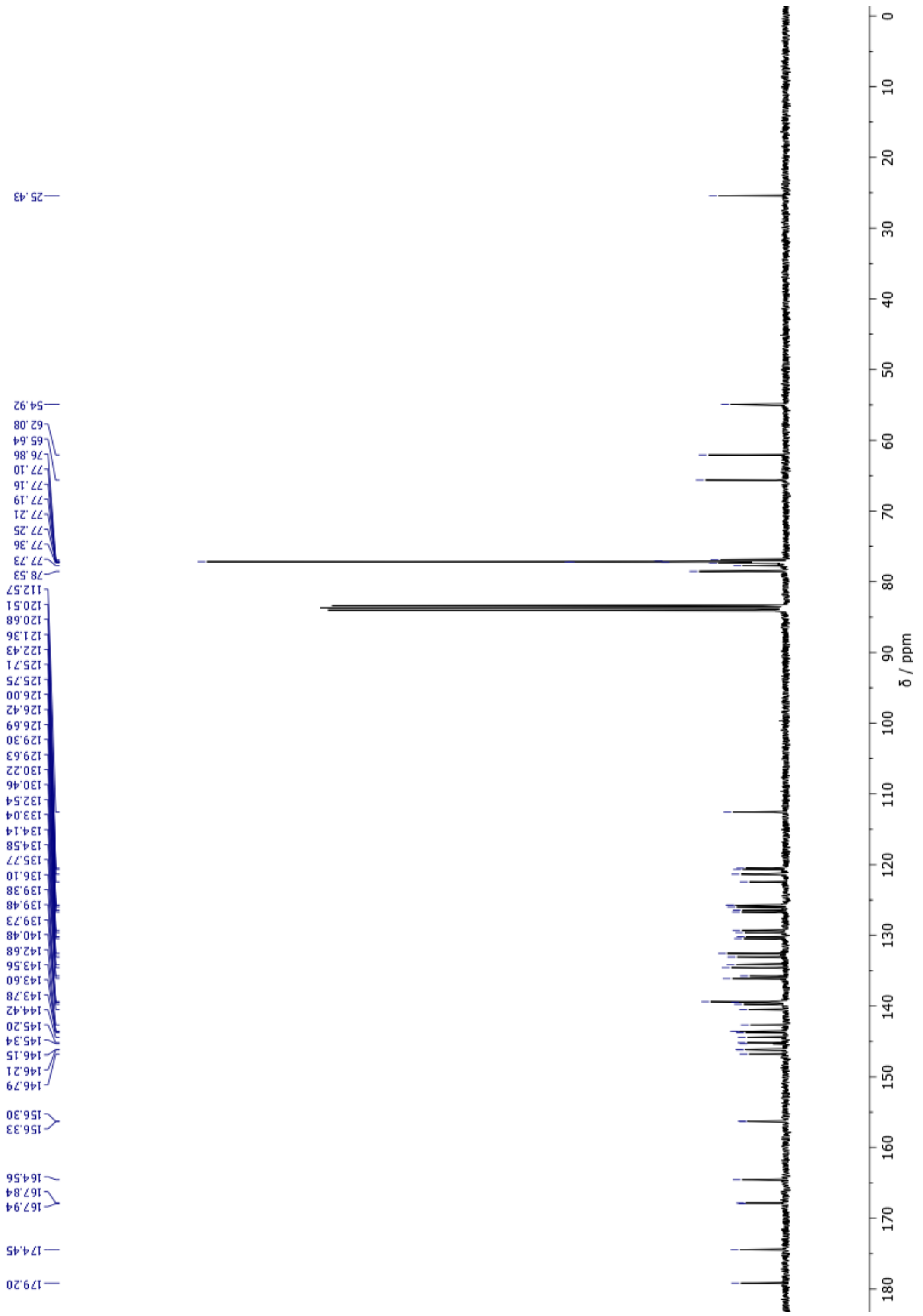

^{13}C NMR spectrum (101 MHz, $CDCl_3$, 298 K) of **1**

References

26. Y. Hamuro, S. J. Geib and A. D. Hamilton, *Angew. Chemie Int. Ed. English*, **1994**, 33, 446.

30. S. J. Pike, A. Heliot and C. C. Seaton, *CrystEngComm*., **2020**, 22, 5040.

31. A. R. Davis, S. Ozturk, C. C. Seaton, L. Male and S. J. Pike, *Chem. Eur. J.,* **2024**, *30,* e202402892.

43. E. Caldeweyher, S. Ehlert, A. Hansen, H. Neugebauer, S. Spicher, C. Bannwarth and S. Grimme, *J. Chem. Phys.,* 2019, **150**, 154122.

44. A. D. Becke, *J. Chem. Phys.* 1997, **107**, 8554.

45. V. Barone and M. Cossi, *J. Phys. Chem. A,* 1998, **102**, 1995.

47. O. V. Dolomanov, L. J. Bourhis, R. J. Gildea, J. A. K. Howard and H. Puschmann, *J. Appl. Cryst.*, **2009**, *42*, 339.

48. G. M. Sheldrick, *Acta Crystallogr. Sect. A: Found. Crystallogr.*, **2015**, *A71*, 3.

49. G. M. Sheldrick, *Acta Crystallogr. Sect. C: Found. Crystallogr.*, **2015**, *C71*, 3.

50. R. N. K. Deepka, R. Sankararmakrishnan, *Biophys. J.*, 2016, **110**, 1967.

51. Short contacts between the N atoms of the azo bonds and the H atom of the adjacent amine group are observed in all solid state structures of **4** and these could serve to further stabilise the helical structure of the scaffold. Similar short contacts have been reported by Parquette and co-workers in their related pyridinedicarboxamide/m-(phenylazo)azobenzene foldamers see C. Tie, J. C. Gallucci and J. R. Parquette, *J. Am. Chem. Soc.*, 2006, **128**, 1162.

52. S. Horowitz and R. C. Trievel, *J. Biol. Chem.*, 2012, **287**, 41576.

53. M. Brandl, M. S. Weiss, A. Jabs, J. Sühnel and R. Hilgenfeld, *J. Mol. Biol.,* 2001, **307**, 357.

54. M. Egli, V. Tereshko, G. N. Mushudov, R. Sanishvili, X. Liu and F. D. Lewis, *J. Am. Chem. Soc.*, 2003, **125**, 10842.

55. F. Neese, *WIRES Comput. Mol. Sci.,* 2012, **2**, 73.
